# Exploring the components and mechanisms of Shen-qi-wang-mo granule in the treatment of retinal vein occlusion by UPLC-Triple TOF MS/MS and network pharmacology

**DOI:** 10.1038/s41598-023-32472-0

**Published:** 2023-04-01

**Authors:** Yi Zhao, Cui Ma, Qinghua Qiu, Xucong Huang, Xijier Qiaolongbatu, Han Qu, Jiaqi Wu, Guorong Fan, Zhenghua Wu

**Affiliations:** 1 Department of Clinical Pharmacy, Shanghai General Hospital, Shanghai Jiao Tong University School of Medicine, Shanghai, 200080 People’s Republic of China; 2 School of Pharmacy, Shanghai Jiao Tong University, Shanghai, 200240 People’s Republic of China; 3 Department of Ophthalmology, Shanghai General Hospital, Shanghai Jiao Tong University School of Medicine, Shanghai, 200080 People’s Republic of China; 4 School of Pharmacy, Chongqing Medical University, Chongqing, 400016 People’s Republic of China

**Keywords:** Clinical pharmacology, Computational biology and bioinformatics, Eye diseases

## Abstract

This study aimed to explore the substance basis and mechanisms of Shen-qi-wang-mo Granule (SQWMG), a traditional Chinese medicine prescription that had been clinically utilized to treat retinal vein occlusion (RVO) for 38 years. Components in SQWMG were analyzed by UPLC-Triple-TOF/MS and a total of 63 components were identified with ganoderic acids (GA) being the largest proportion. Potential targets of active components were retrieved from SwissTargetPrediction. RVO-related targets were acquired from related disease databases. Core targets of SQWMG against RVO were acquired by overlapping the above targets. The 66 components (including 5 isomers) and 169 targets were obtained and concluded into a component-target network. Together with biological enrichment analysis of targets, it revealed the crucial role of the “PI3K-Akt signaling pathway”, “MAPK signaling pathway” and their downstream factor iNOS and TNF-α. The 20 key targets of SQWMG in treating RVO were acquired from the network and pathway analysis. The effects of SQWMG on targets and pathways were validated by molecular docking based on AutoDock Vina and qPCR experiment. The molecular docking showed great affinity for these components and targets, especially on ganoderic acids (GA) and alisols (AS), which were both triterpenoids and qPCR exhibited remarkably reduced inflammatory factor gene expression through regulation of these two pathways. Finally, the key components were also identified from rat serum after treatment of SQWMG.

## Introduction

Retinal vein occlusion (RVO) is the second most common retinal vascular disease after diabetic retinal disease and one of the most common causes of sudden painless unilateral vision loss^1^. RVO includes two main types: branch retinal vein occlusion (BRVO) and central retina vein occlusion (CRVO). The International Eye Disease Consortium reported the prevalence of RVO studies which shows that the prevalence of RVO was 0.52% for any RVO, 0.44% for BRVO, and 0.08% for CRVO. It suggested that roughly 16 million people in the world suffer from RVO and BRVO is about 5 times CRVO^2,3^. The main clinical manifestations of RVO are a sudden loss of vision (CRVO) or blurring in the visual field (BRVO) which is generally asymptotic and painless at the early stages but could aggravate the condition due to intraretinal hemorrhage, retinal edema, and other vascular disorders^4,5^.

Clinically, corticosteroids are commonly used in macular edema caused by RVO. Because vascular disorders and inflammation play a significant role in the pathogenesis of RVO, the anti-VEGF monoclonal antibody taking place of corticosteroids becomes crucial in drug therapy. Anti-VEGF drug Ranibizumab was approved by FDA in June 2010 for the treatment of macular edema secondary to BRVO^4^. With the development of VEGF antagonist injections for eyes, most patients have received surprising vision improvement. Nevertheless, its therapeutic outcomes are not always consistent with expectations. About half of the patients with BRVO and over half of those with CRVO still require anti-VEGF injections for 5 years or more after starting treatment and long-term outcomes are still unknown^6^. Even more, some patients with RVO followed monthly anti-VEGF injections for about 5 months but it did not eliminate macular oedema^6^. So there’s more effort to be put into drug research for RVO and the abundant traditional Chinese medicine (TCM) might show us a new way.

TCM ophthalmology believes that blood stasis, blood deficiency, phlegm, and blood stasis are the main syndromes of RVO. TCM took promoting blood circulation, removing blood stasis, regulating qi, and dredging collaterals as the treatment principle of RVO and achieved good clinical effects. TCM has the characteristics of multi-target, multi-pathway, and multi-mechanism. Shen-qi-wang-mo granule (SQWMG) is a component preparation of TCM for the treatment of fundus diseases in Shanghai General Hospital. The ophthalmology of the hospital is a national key clinical specialty. SQWMG has a remarkable effect on the clinical use of ophthalmology in the hospital and it has been used clinically to treat RVO for 38 years. A clinical study including 120 patients with RVO revealed that the improvement after treatment was statistically significant (P < 0.05) in the SQWMG-Pancreatic kininogenase combination compared to a single drug^7^. The prescription is composed of 13 herbs: Rehmannia Glutinosa Libosch and Rehmanniae Radix Praeparata (SDH), Fructus Ligustri Lucidi (NZZ), Poria Cocos (Schw.) Wolf. (FL), Codonopsis Radix (DS), Angelicae Sinensis Radix (DG), Alisma Orientale (Sam.) Juz. (ZX), Carthami Flos (HH), Lycii Fructus (GQZ), Radix Paeoniae Rubra and Paeoniae Radix Alba (SY), Plantaginis Herba (CQC), and Ganoderma Lucidum (LZ). This prescription has the effects of brightening the eyes, nourishing the liver and kidneys, and promoting blood circulation and diuresis. However, as a component preparation of TCM, its pharmacological basis was unknown and the mechanism of action remained unclear. To overcome the complex obstacles in clarifying the mechanism of TCM, “Network pharmacology” had been introduced and utilized with the development of the multi-omics study, system biology, and chemical biology. By following the route of “prescription-ingredient-component-target-pathway-disease” and constructing the “drug-component-targets” (CTD) network, it provided an efficient way to investigate the pharmacological effects and potential mechanisms of TCM systematically^8^. It had been successfully applied to explore the mechanism of profound TCM herbal formulae such as Shexiang Baoxin Pill (SBP), and Qingfei Xiaoyan Pill (QFXYP), and diseases including myocardial infarction, cancer, and even COVID-19^9,10^.

**Figure 1:**
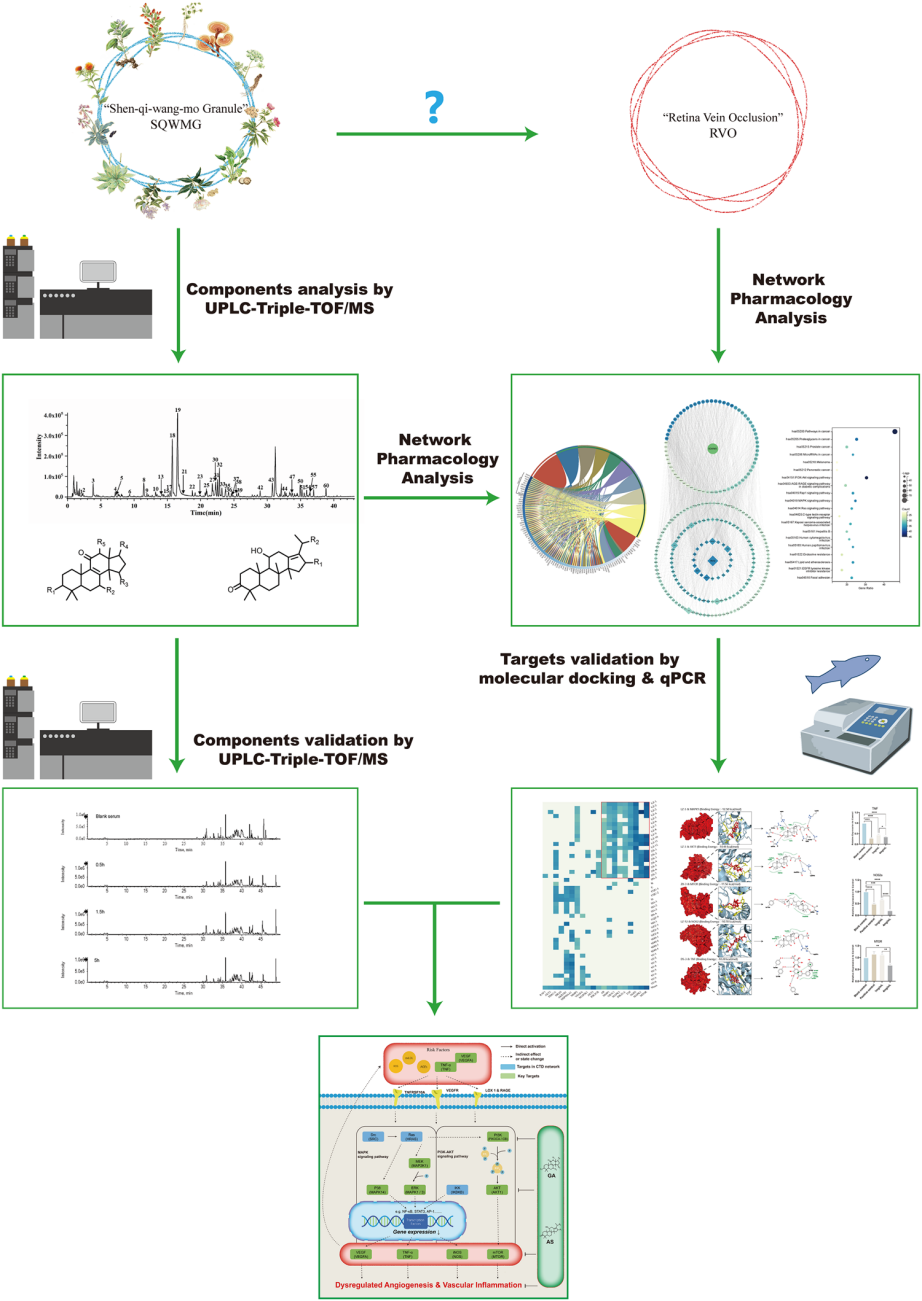
Graph abstract of the whole article.

In this study, as shown in Fig. 1, we combined UPLC-Triple-TOF/MS with network pharmacology to create a network for the active components and therapeutic mechanism of SQWMG. Then we employed molecular docking to verify the key targets. Next, in vivo experiment was carried out to validate the therapeutic effect of SQWMG utilizing zebrafish as a model organism^11^. The key components were also identified from rat serum. Together, this study followed the guidelines for network pharmacology evaluation and revealed the potential mechanism and substance basis of SQWMG in treating RVO and provides a certain basis for future exploration, also providing new ideas for developing new drugs^12^.

## Results

### The clinical application of SQWMG

SQWMG has been used clinically in Shanghai General Hospital for almost 40 years with obvious therapeutic effects recognized by patients and clinical trials^7^. In the past 6 months (2022/6/1–2022/11/31), the number of outpatient prescriptions for SQWMG in our hospital reached 781, of which 290 were patients with fundus diseases related to retinal vein occlusion. It is worth noting that nearly half of the prescriptions (387) were re-prescription that were repeated more than twice, indicating the efficacy of the drug and the recognition of patients (Supplementary Fig. 1). It is very valuable to explore the substance basis of SQWMG.

### Analysis of SQWMG components

**Figure 2:**
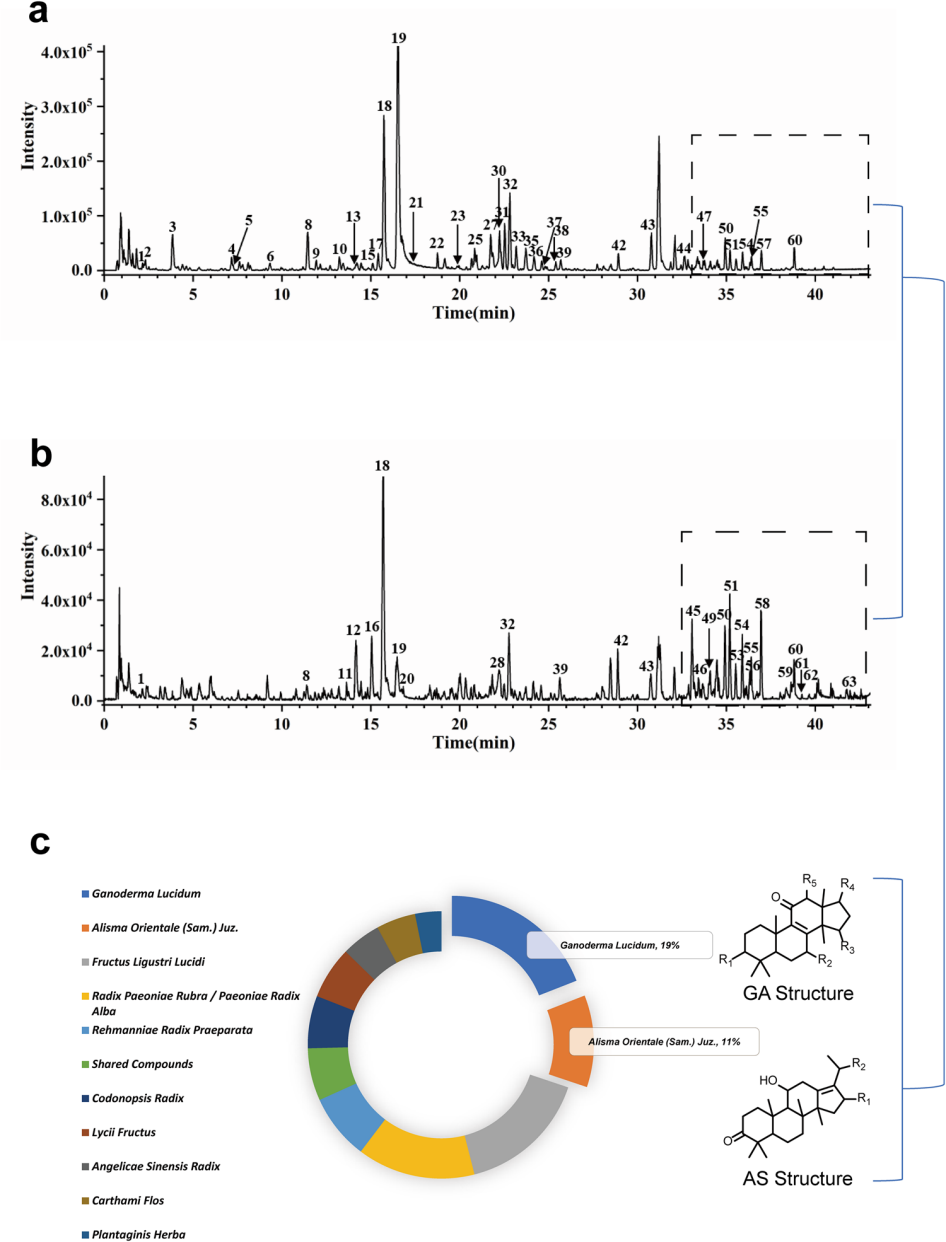
Components analysis of SQWMG. Base peak ion flow diagram of SQWMG samples under negative ion (**a**) and positive ion (**b**) by UPLC-Triple-TOF/MS. (**c**) The proportion of each ingredient and main structure of GA and AS.

The sample of SQWMG was analyzed on Ultra performance liquid chromatography-high resolution mass spectrometry (UPLC-Triple-TOF/MS) and 63 components (Table 1) were identified from multistage mass spectrum information combined with the database of high-resolution mass spectrometry of natural products and related literature. In addition, all the components with comprehensive information come from actual spectrometry of standard substances without any simulation or speculation which made the accuracy of the matching result credible. The components which could not be found in the database like GQZ-1~8 (Lycibarbarspermidine), were identified from a fragmental pattern or relative literature. All component information including name, retention time (tr), and MS/MS data were shown in Table 1 and the base peak intensity chromatogram (BPI) was shown in Fig. 2. The relative MS/MS spectrometry of all 63 components was shown in Supplementary Figure 2.

**Table 1:** Components identified from SQWMG.

No	t_r_/min	ID	Ion form	Theoretical m/z	Measured m/z	Error (ppm)	Element Composition	MS/MS	Name	Source
1	2.18	A	[M-H]^−^	243.0623	243.0622	− 0.2	C_9_H_12_N_2_O_6_	243.0641;200.0576;152.0373;122.0254;110.0257	Uridine	All
2	2.28	SDH-5	[M + FA-H]^−^	407.1195	407.1191	− 1	C_15_H_22_O_10_	361.1127;223.0585;199.0603;181.0490;169.0504;	Catalpol	Rehmanniae Radix Praeparata
3	3.86	SY-7	[M-H]^−^	169.0142	169.0146	2.1	C_7_H_6_O_5_	169.0146;125.0251;107.0146;97.0305;79.0190	Gallic acid	Radix Paeoniae Rubra / Paeoniae Radix Alba
4	7.17	SY-8	[M-H]^−^	493.1199	493.1211	2.4	C_19_H_26_O_15_	493.1208;313.0570;283.0460;169.0146;125.0249	1'-O-Galloylsucrose	Radix Paeoniae Rubra / Paeoniae Radix Alba
5	7.25	SDH-4	[M + FA-H]^−^	731.2251	731.2279	3.8	C_27_H_42_O_20_	685.2188;505.1621;341.1050;263.0757;221.0642;179.0550	Rehmannioside D	Rehmanniae Radix Praeparata
6	9.32	NZZ-8	[M-H]^−^	299.1136	299.1126	− 3.4	C_14_H_20_O_7_	137.0248;119.0483;113.0242;101.0258;89.0250	Salidroside	Fructus Ligustri Lucidi
7	10.2	DG-1	[M-H]^−^	353.0878	353.088	0.5	C_16_H_18_O_9_	353.0848;191.0561;179.0345;135.0460	Neochlorogenic acid	Angelicae Sinensis Radix
8	11.46	NZZ-10	[M-H]^−^	433.0988	433.0999	2.6	C_17_H_22_O_13_	433.0973;389.1072;221.0089;209.0453;177.0195;165.0557	Hydroligulucidumoside C (-C_2_H_4_)	Fructus Ligustri Lucidi
9	11.93	NZZ-7	[M-H]^−^	389.1089	389.1103	3.5	C_16_H_22_O_11_	389.1078;345.1183;209.0453;183.0650;165.0559;121.0655	Secoxyloganic acid	Fructus Ligustri Lucidi
10	13.24	HH-3	[M-H]^−^	611.1618	611.164	3.7	C_27_H_32_O_16_	611.1653;491.1223;473.1100;403.1058;325.0743;283.0628	Hydroxysafflor yellow A	Carthami Flos
11	13.65	GQZ-6/7/8	[M + H]^+^	798.3655	798.3667	1.5	C_37_H_55_N_3_O_16_	798.3611;636.3055;474.2588;384.1645;222.1110	Lycibarbarspermidine K/L/M or isomer	Lycii Fructus^14^
12	14.15	GQZ-4/5	[M + H]^+^	636.3127	636.3116	− 1.7	C_31_H_45_N_3_O_11_	636.3112;474.2617;384.1663;222.1128	Lycibarbarspermidine H/I or isomer	Lycii Fructus^14^
13	14.16	DG-2	[M-H]^−^	353.0878	353.0875	− 0.9	C_16_H_18_O_9_	353.0884;191.0568;179.0355;173.0464;135.0458	Chlorogenic acid	Angelicae Sinensis Radix
14	14.55	DG-3	[M-H]^−^	353.0878	353.0884	1.7	C_16_H_18_O_9_	353.0922;191.0571;179.0368;161.0248;127.0426	Cryptochlorogenic acid	Angelicae Sinensis Radix
15	15.1	DS-4	[M + FA-H]^−^	471.2083	471.2102	4	C_18_H_34_O_11_	425.2018;263.1492;161.0451	Hexyl 2-O-β-D-glucopyranosyl-β-D-glucopyranoside	Codonopsis Radix
16	15.11	GQZ-1/2/3	[M + H]^+^	796.3499	796.3503	0.6	C_37_H_53_N_3_O_16_	796.3531;634.2986;472.2631;382.1479;220.0972	Lycibarbarspermidine E/F/G or isomer	Lycii Fructus^14^
17	15.43	SY-9	[M + FA-H]^−^	687.2142	687.215	1.2	C_29_H_38_O_16_	641.2076;611.1987;593.1870;489.1601;165.0550;121.0295	β-Gentiobiosyl paeoniflorin	Radix Paeoniae Rubra / Paeoniae Radix Alba
18	15.75	SY-6	[M + FA-H]^−^	525.1614	525.1627	2.5	C_23_H_28_O_11_	525.1638;479.1568;357.1204;283.0829;121.0298	Albiflorin	Radix Paeoniae Rubra / Paeoniae Radix Alba
19	16.52	SY-1	[M + FA-H]^−^	525.1614	525.1605	− 1.6	C_23_H_28_O_11_	479.1682;449.1471;327.1083;165.0557;121.0298	Paeoniflorin	Radix Paeoniae Rubra / Paeoniae Radix Alba
20	16.84	GQZ-9	[M + H]^+^	472.2442	472.243	− 2.6	C_25_H_33_N_3_O_6_	472.2433;310.2116;220.0962;163.0380	N1-Caffeoyl-N3-dihydrocaffeoylspermidine	Lycii Fructus
21	17.58	HH-2	[M-H]^−^	611.1618	611.1646	4.6	C_27_H_32_O_16_	611.1622;521.1370;449.1085;313.0695;287.0556	Hydroxysafflor yellow B	Carthami Flos
22	18.76	SDH-1	[M-H]^−^	785.251	785.2508	− 0.2	C_35_H_46_O_20_	785.2533;623.2210;477.1599;161.0239	Echinacoside	Rehmanniae Radix Praeparata
23	19.96	DS-2	[M + FA-H]^−^	603.2294	603.2309	2.4	C_26_H_38_O_13_	603.2288;557.2231;467.1771;323.0968;221.0659;179.0552	Lobetyolinin	Codonopsis Radix
24	20.68	SDH-3	[M-H]^−^	799.2666	799.2676	1.2	C_36_H_48_O_20_	799.2674;623.2197;605.2070;461.1643;193.0504;	Jionoside A1	Rehmanniae Radix Praeparata
25	20.86	NZZ-9	[M-H]^−^	569.1512	569.1531	3.3	C_25_H_30_O_15_	569.1491;525.1574;389.0865;363.1067;331.0804;209.0456	Oleuropeinic acid	Fructus Ligustri Lucidi
26	20.95	CQC-1	[M-H]^−^	639.1931	639.1935	0.7	C_29_H_36_O_16_	639.1922;477.1602;315.1063;161.0240	Plantamoside	Plantaginis Herba
27	21.75	SY-3	[M-H]^−^	631.1668	631.1652	− 2.6	C_30_H_32_O_15_	631.1672;613.1589;491.1229;399.0956;313.0570;271.0467	Galloylpaeoniflorin	Radix Paeoniae Rubra / Paeoniae Radix Alba
28	22.11	SY-5	[M + H]^+^	481.1704	481.1703	− 0.3	C_23_H_28_O_11_	301.1070;197.0805;179.0719;151.0761;133.0646;105.0330	Mudanpioside I	Radix Paeoniae Rubra / Paeoniae Radix Alba
29	22.18	DS-1	[M + FA-H]^−^	441.1766	441.1766	0	C_20_H_28_O_8_	305.1202;215.1089;185.0963;159.0803;143.0702;119.0344	Lobetyolin	Codonopsis Radix
30	22.25	C	[M-H]^−^	623.1981	623.1987	0.9	C_29_H_36_O_15_	623.2002;461.1661;315.1094;161.0252	Acteoside	Rehmanniae Radix Praeparata/Plantaginis Herba
31	22.53	CQC-2	[M-H]^−^	639.1931	639.1929	− 0.2	C_29_H_36_O_16_	639.1948;477.1650;315.1093;161.254	Plantainoside D	Plantaginis Herba
32	22.82	NZZ-2	[[M-H]^−^	685.2349	685.2384	5.1	C_31_H_42_O_17_	685.2324;523.1817;453.1405;421.1496;299.1132;223.0609	Specnuezhenide	Fructus Ligustri Lucidi
33	23.18	D	[M-H]^−^	623.1981	623.201	4.6	C_29_H_36_O_15_	623.2005;461.1672;315.1095;161.0247	Isoacteoside	Rehmanniae Radix Praeparata/Plantaginis Herba
34	23.44	HH-1	[M-H]^−^	593.1512	593.1538	4.4	C_27_H_30_O_15_	593.1498;285.0399;255.0297;227.0362	Kaempferol-3-O-rutinoside	Carthami Flos
35	23.76	NZZ-1	[M + FA-H]^−^	731.2404	731.2406	0.3	C_31_H_42_O_17_	685.2376;523.1853;453.1417;299.1141;223.0618;179.0563	Isonuezhenide	Fructus Ligustri Lucidi
36	24.2	SY-2	[M-H]^−^	631.1668	631.1674	0.9	C_30_H_32_O_15_	631.1680;399.0990;313.0539;271.0440;169.0148;151.0033	4'-O-Galloylpaeoniflorin	Radix Paeoniae Rubra / Paeoniae Radix Alba
37	24.62	NZZ-3	[M-H]^−^	685.2349	685.2361	1.7	C_31_H_42_O_17_	685.2339;523.1796;453.1413;421.1500;299.1124;223.0619	Nuezhenide	Fructus Ligustri Lucidi
38	25.42	NZZ-5	[M-H]^−^	539.177	539.1796	4.8	C_25_H_32_O_13_	539.1774;377.1232;307.0830;275.0926;149.2053;119.0353	Oleuropein	Fructus Ligustri Lucidi
39	25.68	DS-3	[M-H]^−^	445.0776	445.0773	− 0.8	C_21_H_18_O_11_	269.0432;251.0330;241.0498;223.0397;195.0444;113.0242	Baicalin	Codonopsis Radix
40	27.74	SDH-2	[M-H]^−^	651.2294	651.2324	4.5	C_31_H_40_O_15_	651.2278;475.1856;193.0526;175.0402;160.0159	Martynoside	Rehmanniae Radix Praeparata
41	28.1	NZZ-6	[M-H]^−^	519.1508	519.1528	3.9	C_25_H_28_O_12_	519.1573;227.0556;189.0570;183.0659;161.0603;147.0462	6'-O-Cinnamoyl-8-epikingisidic acid	Fructus Ligustri Lucidi
42	28.95	NZZ-4	[M + FA-H]^−^	1117.362	1117.369	6.4	C_48_H_64_O_27_	1071.3605;909.3102;771.2384;685.2371;523.1843;453.1415	G13	Fructus Ligustri Lucidi
43	30.8	SY-4	[M + FA-H]^−^	629.1876	629.1904	4.5	C_30_H_32_O_12_	583.1837;553.1719;431.1339;165.0557;121.0298	Benzoylpaeoniflorin	Radix Paeoniae Rubra / Paeoniae Radix Alba
44	32.66	B	[M-H]^−^	329.2333	329.2327	− 2	C_18_H_34_O_5_	329.2325;229.1447;211.1335;171.1025;139.1127	9,12,13-Trihydroxy-10-octadecenoic acid	All
45	33.12	LZ-11	[M + FA-H]^−^	505.2807	505.2819	2.4	C_27_H_40_O_6_	459.2764;441.2744;397.2820;303.1974;249.1505	3β,7β,15β-trihydroxy-11-oxo-lanosta-8-en-24 → 20 lactone	Ganoderma
46	33.45	LZ-4	[M + H]^+^	533.3109	533.3126	3.2	C_30_H_44_O_8_	515.2991;497.2904;479.2790;451.2850;367.2279;349.2173	Ganoderic acid G	Ganoderma
47	33.72	LZ-1	[M + H]^+^	517.3003	515.3027	4.6	C_30_H_42_O_7_	515.3001;479.2827;461.2642;359.2218;325.1460	Ganoderic acid V1	Ganoderma
48	34.02	ZX-6	[M + H]^+^	505.3524	505.3539	3	C_30_H_48_O_6_	505.3532;487.3427;469.3307;451.3209;415.2847;397.2728	16-oxoalisol A	Alisma Orientale (Sam.) Juz
49	34.1	LZ-3	[M + H-H_2_O]^+^	499.3054	499.3058	0.8	C_30_H_44_O_7_	499.3057;481.2966;463.2819;445.2716;351.1951;337.1788	Ganoderic acid B	Ganoderma
50	34.94	LZ-2	[M-H]^−^	515.3014	515.302	1.1	C_30_H_44_O_7_	515.3036;497.2928;453.3052;435.2946;300.1727;285.1507	Ganoderic acid A	Ganoderma
51	35.22	LZ-10	[M-H]^−^	571.2913	571.2906	− 1.2	C_32_H_44_O_9_	553.2812;511.2705;481.2207;467.2807;437.2328;	Ganoderic acid H	Ganoderma
52	35.25	LZ-8	[M-H]^−^	457.2596	457.2596	0.1	C_27_H_38_O_6_	457.2596;439.2498;395.2562;287.1681;247.1344;209.1186	Lucidenic acid A	Ganoderma
53	35.56	LZ-9	[M-H]^−^	511.2701	511.2712	2.1	C_30_H_40_O_7_	493.2587;478.2352;449.2687;329.1720;301.1770;149.0607	Ganoderenic acid D	Ganoderma
54	35.93	LZ-6	[M-H]^−^	513.2858	513.2873	3	C_30_H_42_O_7_	495.2753;451.2853;301.1810;283.1692;247.1333;149.0606	Ganoderic acid D	Ganoderma
55	36.42	LZ-12	[M-H]^−^	511.2701	511.2702	0.1	C_30_H_40_O_7_	511.2728;493.2604;449.2693;434.2458;419.2212;149.0612	23-hydroxy-3,7,11,15-tetraoxo-lanost-8,24e-diene-oic acid	Ganoderma
56	36.49	LZ-7	[M + H]^+^	501.3211	501.3224	2.7	C_30_H_44_O_6_	501.3180;483.3119;465.3010;447.2883;353.2476	Ganoderic acid BETA	Ganoderma
57	36.97	LZ-5	[M-H]^−^	569.2756	569.2777	3.7	C_32_H_42_O_9_	551.2663;509.2554;479.2038;465.2636;435.2142;299.1623	Ganoderic acid F	Ganoderma
58	36.98	ZX-2	[M + H]^+^	487.3418	487.344	4.5	C_30_H_46_O_5_	487.3451;469.3355;451.3236;397.2774;353.2513	Alisol C	Alisma Orientale (Sam.) Juz
59	38.64	ZX-4	[M + H]^+^	529.3524	529.3531	1.4	C_32_H_48_O_6_	529.3527;511.3390;469.3291;451.3201;433.3092;415.2834	Alisol C 23-acetate	Alisma Orientale (Sam.) Juz
60	38.84	ZX-5	[M + FA-H]^−^	535.364	535.364	− 0.1	C_30_H_50_O_5_	535.3675;489.3662;471.3513;395.2952;339.2695	Alisol A	Alisma Orientale (Sam.) Juz
61	39.26	ZX-1	[M + H-H_2_O]^+^	515.3731	515.3732	0.2	C_32_H_52_O_6_	515.3686;497.3648;437.3461;383.2945;365.2868;339.2662	Alisol A 23-acetate	Alisma Orientale (Sam.) Juz
62	39.99	ZX-7	[M + H-H_2_O]^+^	515.3731	515.3753	4.3	C_32_H_52_O_6_	515.3739;497.3642;437.3446;383.2946;365.2835;339.2684	Alisol A 24-acetate	Alisma Orientale (Sam.) Juz
63	41.96	ZX-3	[M + H]^+^	515.3731	515.373	− 0.2	C_32_H_50_O_5_	515.3776;437.3443;419.3320;357.2803;339.2694;	Alisol B 23-acetate	Alisma Orientale (Sam.) Juz

The proportion of each herb's ingredients was shown in Fig. 2c. Among these 63 components, 12 components of Ganoderma Lucidum origin made up the largest proportion. These Ganoderma-derived components also have considerable structural consistency: all twelve species were various ganoderic acids (GA) and their derivatives which all belonged to triterpenoids, suggesting to us that ganoderic acids might play an important role in SQWMG. Not only Ganoderma-derived components but also the components of Alisma Orientale (Sam.) Juz. sources showed a high degree of structural consistency. All seven components from Alisma Orientale (Sam.) Juz. were typical Alisol (AS) and its derivatives which, surprisingly, were also triterpenoids as shown in Fig. 2c. Triterpenoids, especially naturally sourced triterpenoids, had been studied for their profound therapeutic activities including anti-inflammatory, antiulcerogenic, antimicrobial, and antiviral activity^13^.

### Potential targets of components

**Figure 3:**
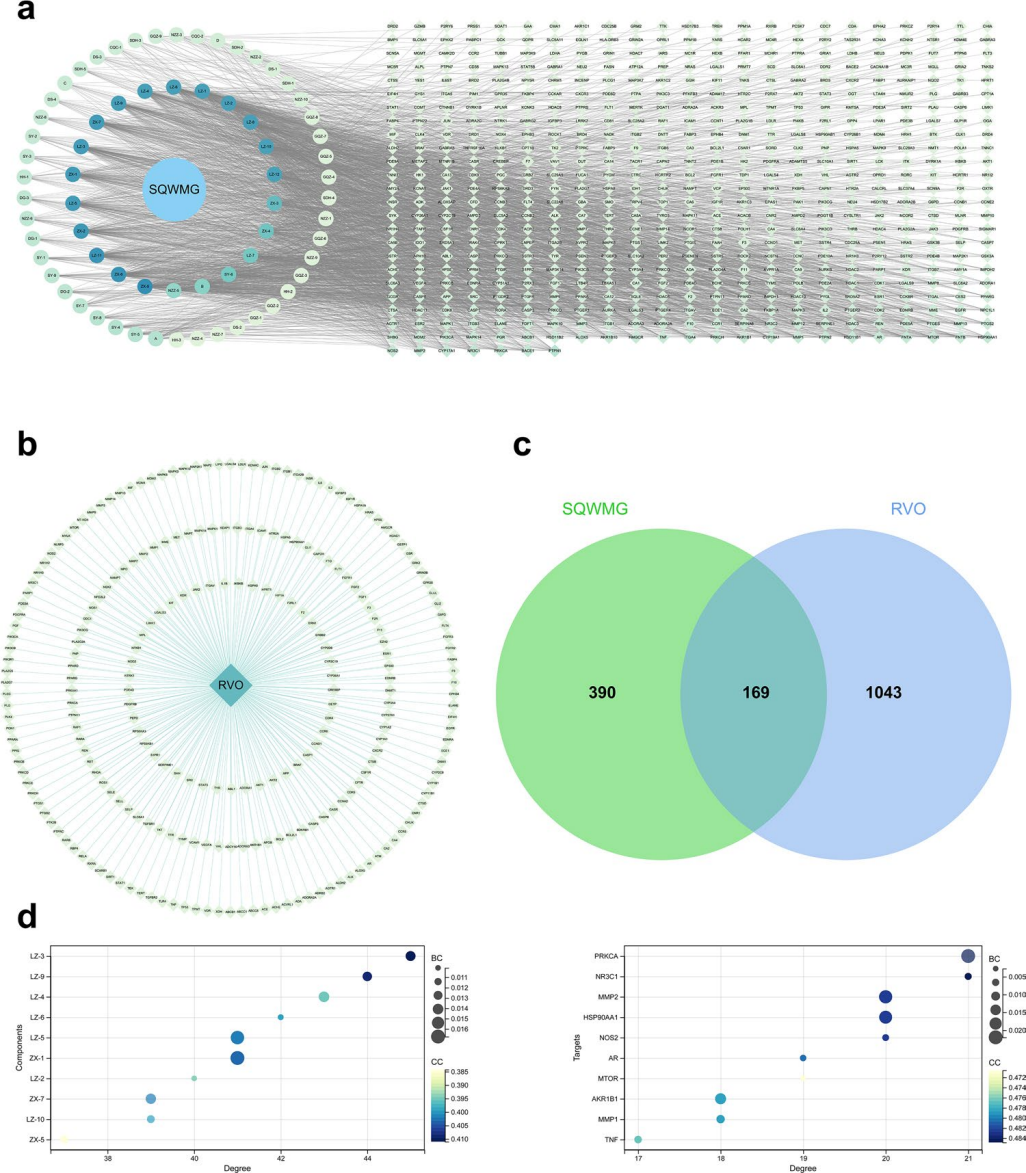
The network analysis of potential targets. (**a**) Components-target network of SQWMG. (**b**) Disease-target network of RVO. (**c**) Venn diagram of related targets of RVO and SQWMG, (**d**) The most connective nodes in the CTD network. The color of the nodes in (**a**) and (**b**) represents the degree value.

Through SwissTargetPrediction, potential drug targets were searched and obtained by selecting the effective results (probability score > 0). A total of 559 targets were included in the next step (Supplementary Table 1), and a drug-components-target network was constructed for a brief analysis (Figure 3a). In network analysis, degree values represented how connective the nodes were. Components with higher degree value meant they might have affected more targets. Deeper and further studies are needed, however, to decide whether it’s the key component or not in treating RVO and how it works.

**Figure 4:**
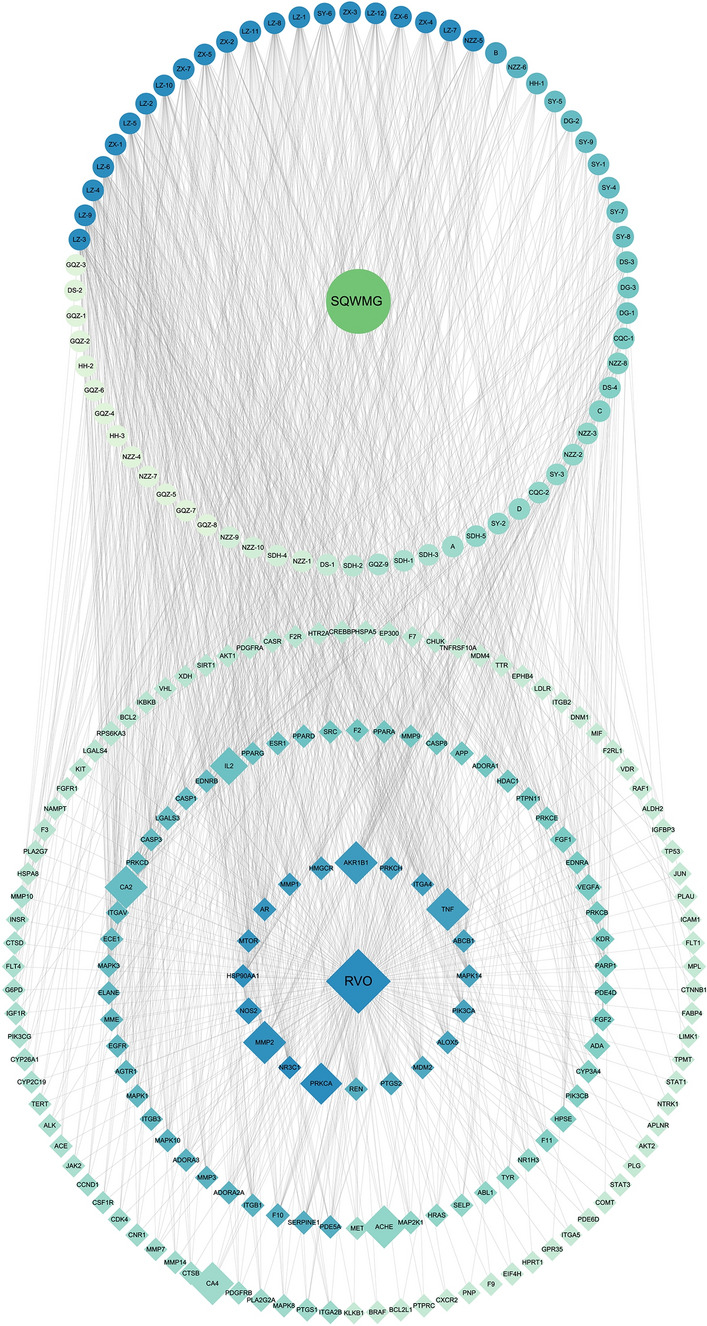
The CTD network merging from the components-target network and disease-target network, contains 169 potential targets from 66 components. The color of the nodes represents the degree value and the probability of each target was shown as node size.

A disease-related target analysis was then conducted based on GeneCards, DisGeNET, and OMIM databases. In GeneCards, RVO-related gene targets show 2015 results, and 1007 targets with higher relevance scores were included. Another 76 genes were obtained in DisGeNET and 212 genes from OMIM. Final RVO-related targets contained 1212 genes by crossing results from GeneCards, DisGeNET, and OMIM. All target information was shown in Supplementary Table 1. A disease-Target network was then constructed (Figure 3b). The drug-disease overlapping targets Venn diagram was created using VENNY 2.1 software, and 169 overlapping targets were identified (Figure 3c). Finally, merging the drug-components-target network and disease-target network in Cytoscape, a component-target-disease (CTD) network was obtained including all targets related to RVO and SQWMG. Through the final CTD network, the potential targets narrowed to 169 genes with 68 components (including 5 isomers of GZQ) involved as shown in Fig. 4 and Supplementary Table 2. The most connective targets and components in the CTD network are shown in Fig. 3d. The top 10 components were all from Ganoderma lucidum and Alisma Orientale (Sam.) Juz. and, of course, were all triterpenoids. This indicated they affected the most targets and, once again illustrated the crucial role of GA and AS in the treatment of RVO. Top targets with the highest degree included *PRKCA* (protein kinase C alpha, PKC), *NR3C1* (Glucocorticoid Receptor), *MMP2* (Matrix Metalloproteinase-2), *HSP90AA1* (Heat Shock 90kDa Protein 1, Alpha) and *NOS2* (Nitric Oxide Synthase 2, Inducible/iNOS). The inhibition of PKC was believed to be effective in treating VEGF-induced retinal vascular dysfunction^15,16^. *NR3C1* encodes glucocorticoid receptors, upon which glucocorticoids exert most of their effects. Glucocorticoid had a strong anti-inflammatory function and was also a traditional treatment for RVO patients before anti-VEGF therapy^4^. MMP2 was a zinc-dependent protease that plays an important role in many diseases, recent research had revealed its connection to vein thrombosis and RVO^17,18^. INOS is a key enzyme associated with inflammation, retinal ischemia, and pathologic vascular proliferation and had a relation with the activation of the MAPK signal pathway^19–21^. The result of the CTD network offered a primary insight into the mechanism of SQWMG with multi-targets and multi-components.

### Biological network analysis

**Figure 5:**
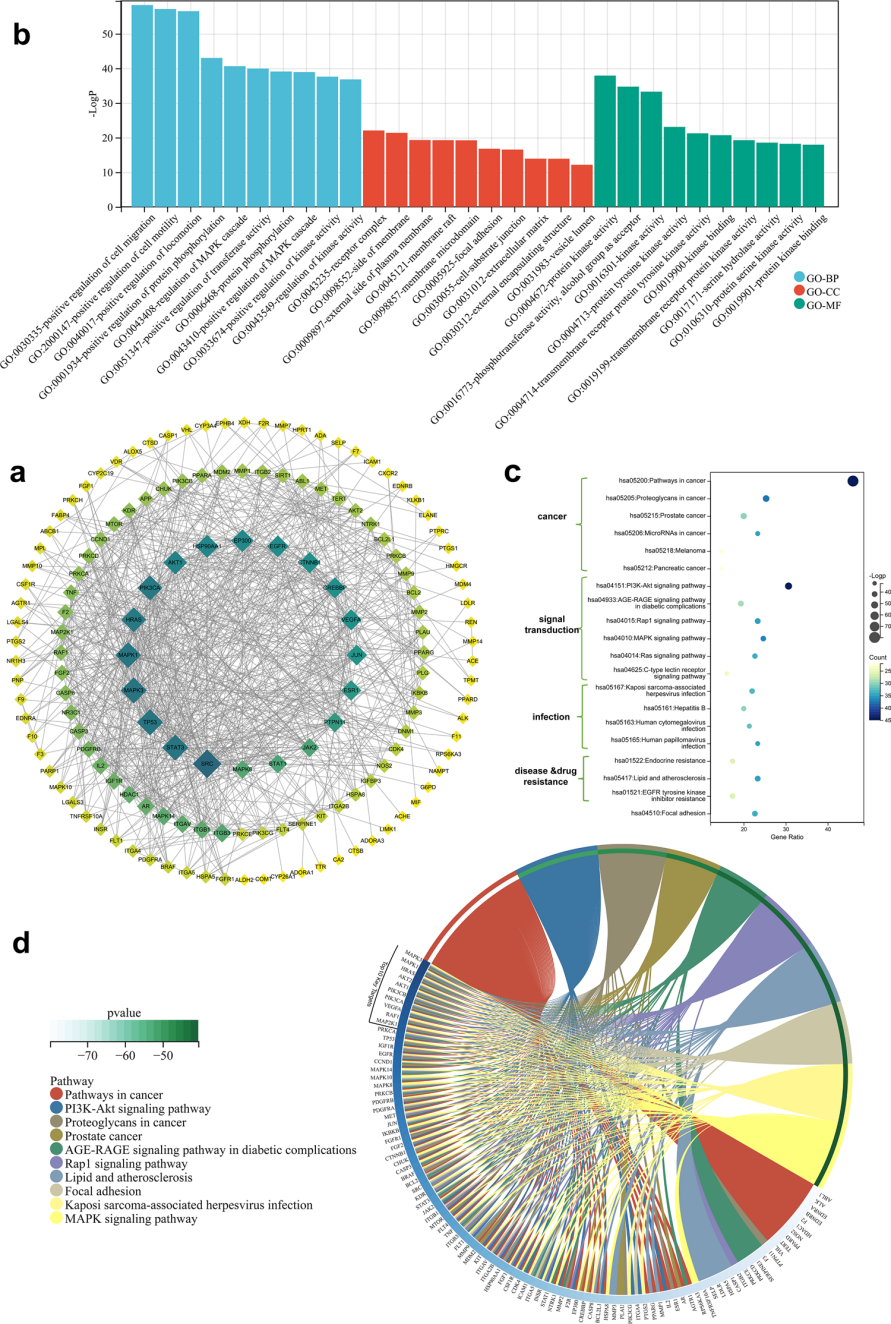
Biological analysis of potential targets. (**a**) PPI network of SQWMG-RVO shared targets. (**b**) GO enrichment analysis. (**c**) Network of top 20 KEGG pathways and targets. (**d**) Circle chart of the top 10 KEGG terms. Colors in the network represent nodes’ degrees and importance.

All 169 potential targets in the CTD network were then introduced to the String database (https://string-db.org/) to perform a protein-protein interaction (PPI) analysis. As the interaction score was set to “highest confidence” (0.9), results showed 19 targets isolated, meaning they possibly hadn’t laid on an effective pathway in treating RVO (Supplementary Table 3). As shown in Fig. 5a, the rest 150 targets showed complex interaction with other potential targets indicating some of them may situate in key pathways. Targets with the strongest interaction with other targets included Proto-oncogene tyrosine-protein kinase Src (*SRC*, D = 49), Mitogen-activated protein kinase 3 (*MAPK3*, D = 42), Signal transducer and activator of transcription 3 (*STAT3*, D = 42), P53 (*TP53*, D = 42) and Mitogen-activated protein kinase 1 (*MAPK1*, D = 41). Then the Gene Ontology (GO) enrichment analysis was conducted by Metascape and the threshold was selected as P < 0.01. The final results of the top 20 terms in GO-BP, GO-CC, and GO-MF were shown in Fig. 5b and Supplementary Table 4. In GO-BP enrichment, positive regulation of cell migration (GO:0030335, LogP = − 58.26) and migration-related terms were at the top place, indicating these genes involved in the PPI network might work by influencing cell movement. Other terms included kinase activity and MAPK signaling activity indicating SQWMG might function through MAPK kinase in vascular regulation. The top results of GO-CC and GO-MF are about membrane component (GO:0043235-receptor complex, GO:0045121-membrane raft, GO:0098857-membrane microdomain, etc) and kinase activity (GO:0004672-protein kinase activity, GO:0016301-kinase activity, etc) which backed the receptor and kinase function.

To further identify the potential pathways of these targets and validate the PPI network, Kyoto encyclopedia of genes and genomes (KEGG) enrichment analysis was performed to evaluate relevant pathways based on Metascape. A total of 150 targets the in PPI network were found to be involved in 199 pathways (P < 0.01) (Supplementary Table 4) and the top 20 pathways of enrichment were associated with signal transduction (6 pathways), cancer disease (6 pathways), viral infection (4 pathways), disease & drug resistance (3 pathways) and focal adhesion (1 pathway) as shown in Fig. 5c. Signaling pathways included Rap1, Ras, MAPK, PI3K-Akt, AGE-RAGE, and C-type lectin. These signaling pathways are closely related to injury, apoptosis, inflammation, vascular regulation, and so on. Even though “pathways in cancer” was the statistically most crucial pathway (Gene Ratio: 46%, LogP = − 79.81), it resulted from numerous research profiles about cancer and had little relation with the pathogenesis of RVO, so it wouldn’t be the focus of the following analysis. Excluded pathways related to cancer, however, were two highly related and important pathways: “PI3K-Akt signaling pathway” (Gene Ratio: 30.67%, LogP = − 51.57) and “MAPK signaling pathway” (Gene Ratio: 24.67%, LogP = − 40.57). The KEGG enrichment circle (Figure 5d) showed the targets’ distribution in the top 10 pathways which demonstrated the most important targets. The top 10 targets were also closely related to these two pathways. These pathways depended heavily on the protein kinase activity which was consistent with the results of GO analysis (Figure 5b).

### Target validation

#### Molecular docking analysis

**Figure 6:**
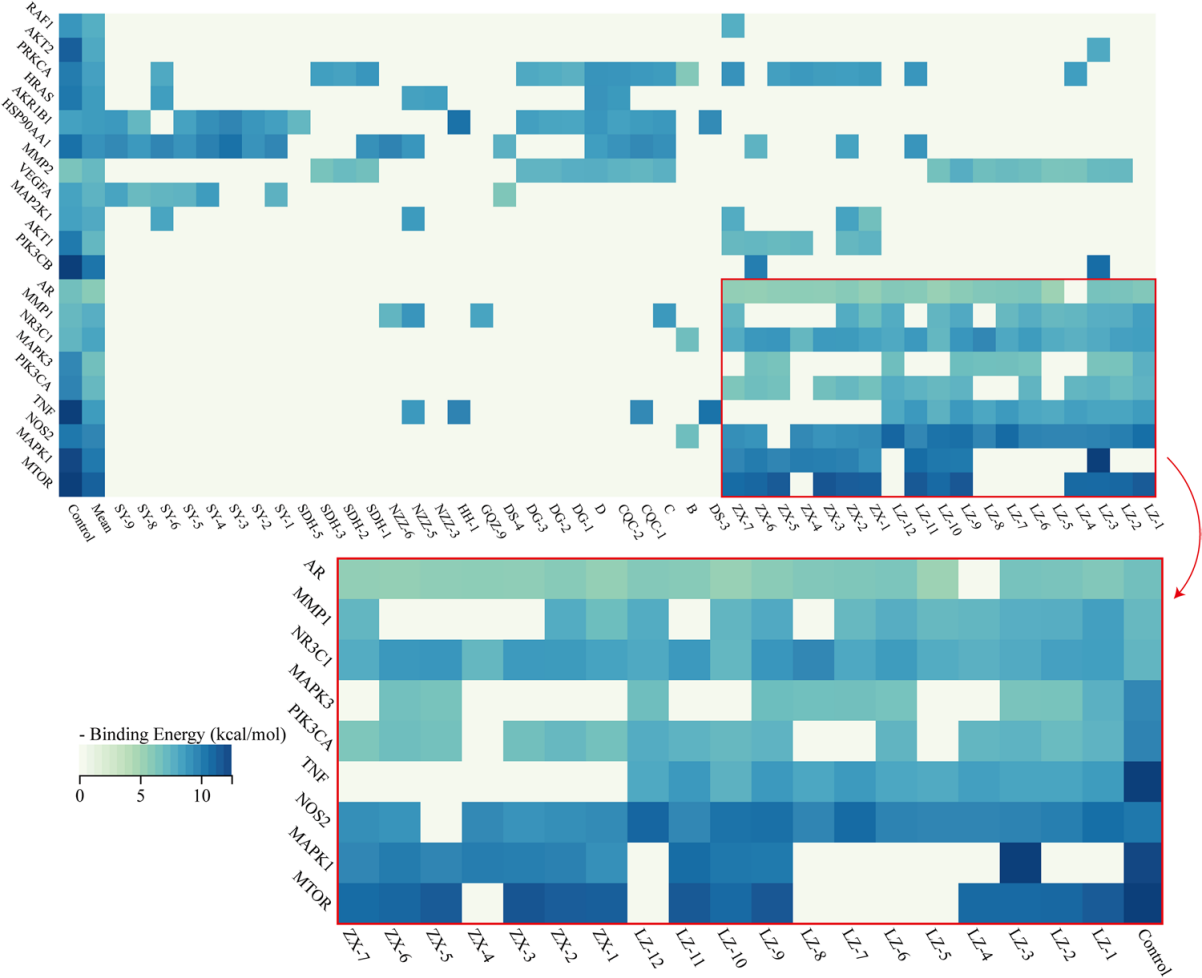
Heatmap of docking result for all 20 targets. GA and AS exhibited great affinity.

The results of molecular docking of components with targets were a predicted interaction between a ligand and a biological macromolecule at the molecular level^22^. Considering the significance of MAPK signaling pathways and PI3K/Akt signaling pathways exhibited in the biological enrichment analysis, we chose the top 10 key protein targets in KEGG pathways (Fig. 5d) and the top 10 targets in the CTD network (Fig. 3d) to conduct molecular docking. The results were presented by binding energy shown in Fig. 6 and Supplementary Table 5. The lower the binding energy, the better the affinity is for the substrate (component) binding to the protein (Target) in that conformation.

All drug components connected to these targets were selected to perform docking together with positive ligands for comparison and drug components exhibited great binding potential with almost all binding energies below – 7 kcal/mol. For comparison, the binding energy of designed ligands was ranging from − 6.14 kcal/mol to − 19.7 kcal/mol as shown in Fig. 6 and Supplementary Table 5. Triterpenoids-derived components, GA and AS, accounted for the majority of docking results. Some of the docking conformations even had binding energy below − 10 kcal/mol with polar interactions for example, in *MTOR*, *NOS2*, *TNF*, and *MAPK1*. These targets were key targets in MAPK and PI3K/Akt/mTOR signaling pathways and the results were solid evidence for KEGG analysis.

**Figure 7:**
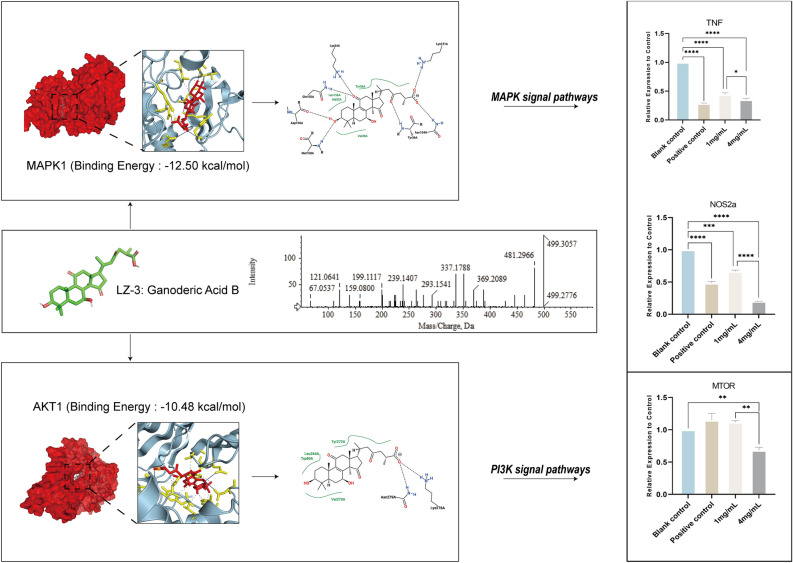
Interaction analysis of key docking result. Interaction mode and specific conformation of docking results with the highest affinity in LZ-3, and extracted ion chromatograms of these components in the ESI + mode by UPLC-Triple-TOF/MS and qPCR analysis of related downstream inflammatory factors. Statistical significance: green * indicates significant differences in the experimental groups relative to the negative control (1.0), and black * indicates significant differences within the experimental groups (n = 2).

Among all components, LZ-3 (Ganoderic acid B), exhibiting the highest affinity in these targets, was shown in Fig. 7 together with its MS/MS data spectrum and its interactions with the proteins. The parent ion of LZ-3 was identified as [M+H-H_2_O]^+^ (C_30_H_44_O_7_, m/z 499.3057) by precise m/z and daughter ion 481.2966, 445.2716, and 351.1951^23^. This result also suggested that the hydroxyl group of LZ-3 underwent protonation and subsequent H_2_O loss during ESI in-source collision-induced dissociation (CID)^24^. The combination of LZ-3 and MAPK1 reached a binding energy of − 12.5 kcal/mol with multiple polar and hydrophobic interactions to stabilize the binding and it also fitted well with AKT1. Both targets were important in MAPK and PI3K/Akt/mTOR signaling pathways and inhibition of these targets may elucidate a strong anti-inflammatory effect by reducing gene expression of inflammatory factors. For inflammatory factors, TNF and NOS2, DS-3, and LZ-12 exhibited great affinity as shown in Supplementary Figure 3.

The result of molecular docking again showed the mechanism of SQWMG was a multi-component and multi-target process. The high affinity of inflammatory factors like iNOS, TNF-α, and MAPK/PI3K- related targets like MAPK1 and AKT1 with GA and AS being key components, was consistent with the results obtained in the previous analysis in this article.

#### Key targets analysis through qPCR

Following the results from molecular docking analysis, we next validated the effects of SQWMG on the expression of MAPK- and PI3K- related downstream factors in vivo using qPCR assay. Dysregulated angiogenesis started by VEGFA involved MAPK and PI3K/Akt signaling pathways causing an abnormal increase of inflammatory factors including iNOS and TNF-α^21,25^. As can be seen in Fig. 7, inflammatory factors were markedly decreased in SQWMG treatment as well as mTOR expression in PI3K/Akt/mTOR signaling pathways, indicating a strong inhibition of vascular inflammation and vascular dysfunction. The results were consistent with the results of previous molecular docking analysis, indicating the effect of SQWMG. Comparing the 1 mg/ml dose with the 4 mg/ml dose, it can also be seen that its effect on target gene expression was generally dose-dependent. Because SQWMG has a multi-pathway and multi-target effect, and downstream inflammatory factors can affect upstream proteins through feedback effects, we also analyzed the gene expression of upstream proteins in MAPK- and PI3K- signaling pathways. The results demonstrated that SQWMG had significant effects on gene expression levels at multiple targets as shown in Supplementary Table 6 and Supplementary Figure 4.

### Analysis of active components in serum

To investigate whether the main components found above can be absorbed to exert pharmacological activity, we analyzed the serum of rats after treatment of SQWMG by UPLC-Triple-TOF/MS. To make the experimental results closer to the situation of clinical use, the animal dose was converted according to the clinical dosage. A total of 12 components were identified from rat serum at different time points after administration as shown in Table 2. All 12 components were included in the analysis of SQWMG components and the following network pharmacology analysis. The component that showed important effects in the network analysis and molecular docking, LZ-3, was also successfully absorbed into the bloodstream for action, which provided convincing support for the previous analysis. The base peak intensity chromatogram (BPI) for all blood samples at three time points was also provided in Supplementary Figure 5.

**Table 2:** Components identified from rats’ serum after treatment of SQWMG.

No.	tr/min	ID	Ion form	Measured m/z	Theoretical m/z	Error (ppm)	Element composition	Name	MS/MS	0.5 h	1.5 h	5 h
S1	2.29	SDH-5	[M + FA-H]^−^	407.1195	407.1195	0	C_15_H_22_O_10_	Catalpol	199.0587;169.0499;151.0402;137.0204	√	√	√
S2	9.28	NZZ-8	[M-H]^−^	299.1121	299.1136	− 5.1	C_14_H_20_O_7_	Salidroside	119.0491;113.0247;101.0248;89.0244;71.0142	√	√	√
S3	11.59	NZZ-10	[M-H]^−^	433.0972	433.0988	− 1.3	C_17_H_22_O_13_	Hydroligulucidumoside C (–C_2_H_4_)	433.0973;389.1072;221.0089;209.0453;177.0195;165.0557	√	√	√
S4	13.36	HH-3	[M-H]^−^	611.1614	611.1618	− 0.6	C_27_H_32_O_16_	Hydroxysafflor yellow A	611.1653;491.1223;473.1100;403.1058;325.0743;283.0628	√	√	√
S5	15.5	SY-9	[M + FA-H]^−^	687.2187	687.2142	6.6	C_29_H_38_O_16_	β-Gentiobiosylpaeoniflorin	641.2076;611.1987;593.1870;489.1601;165.0550;121.0295	√	√	√
S6	15.85	SY-6	[M + FA-H]^−^	525.1594	525.1614	− 3.7	C_23_H_28_O_11_	Albiflorin	525.1733;479.1519;283.0789;121.0290	√	√	√
S7	16.61	SY-1	[M + FA-H]^−^	525.1623	525.1614	1.8	C_23_H_28_O_11_	Paeoniflorin	479.1573;449.1442;327.1062;165.0550;121.0298	√	√	√
S8	22.33	SY-5	[M + FA-H]^−^	525.1613	525.1614	− 0.1	C_23_H_28_O_11_	Mudanpioside I	301.1070;197.0805;179.0719;151.0761;133.0646;105.0330	√	√	√
S9	34.02	ZX-6	[M + H]^+^	505.3503	505.3524	− 4.1	C_30_H_48_O_6_	16-oxoalisol A	505.3532;487.3427;469.3307;451.3209;415.2847;397.2728	√	√	√
S10	34.06	LZ-3	[M + H-H_2_O]^+^	499.308	499.3054	5.2	C_30_H_44_O_7_	Ganoderic acid B	499.3024;481.3136;351.2040;265.1590	√	√	√
S11	34.93	LZ-2	[M-H]^−^	515.3021	515.3014	1.3	C_30_H_44_O_7_	Ganoderic acid A	515.3036;497.2928;453.3052;435.2946;300.1727;285.1507	√	√	√
S12	38.85	ZX-5	[M + FA-H]^−^	535.3651	535.364	2	C_30_H_50_O_5_	Alisol A	535.3675;489.3662;471.3513;395.2952;339.2695	√	√	√

## Discussion

As a TCM prescription used clinically to treat RVO for 38 years, SQWMG had a complex pharmacological network. In this study, we aimed to explore the underlying pharmacological mechanism and substance basis of SQWMG in treating RVO by combining components analysis and network pharmacology.

The aqueous extract of SQWMG and each ingredient was analyzed by UPLC-Triple-TOF/MS and 63 components (Table 1) were identified, of which Ganoderma-derived components (GA) and Alisma Orientale (Sam.) Juz. derived components (AS) made up the largest proportion. Previous studies had found that GA had a marked effect on the inhibition of MAPK-/PI3K-/NF-κB- signaling pathways and inflammatory factors, indicating a potential therapeutic effect on these targets of SQWMG^26–30^. Then we employed network pharmacology, investigating the potential targets by target prediction following the network pharmacology evaluation method guidance^12^. A combination of RVO-related targets and SQWMG-related targets formed a complex CTD network on which some targets exhibited greater importance such as PKC, glucocorticoid receptor, and iNOS. These targets are closely related to RVO and gave a glimpse of the mechanism but were still isolated. Biological analysis was then introduced to find the inner relation of these targets.

**Figure 8:**
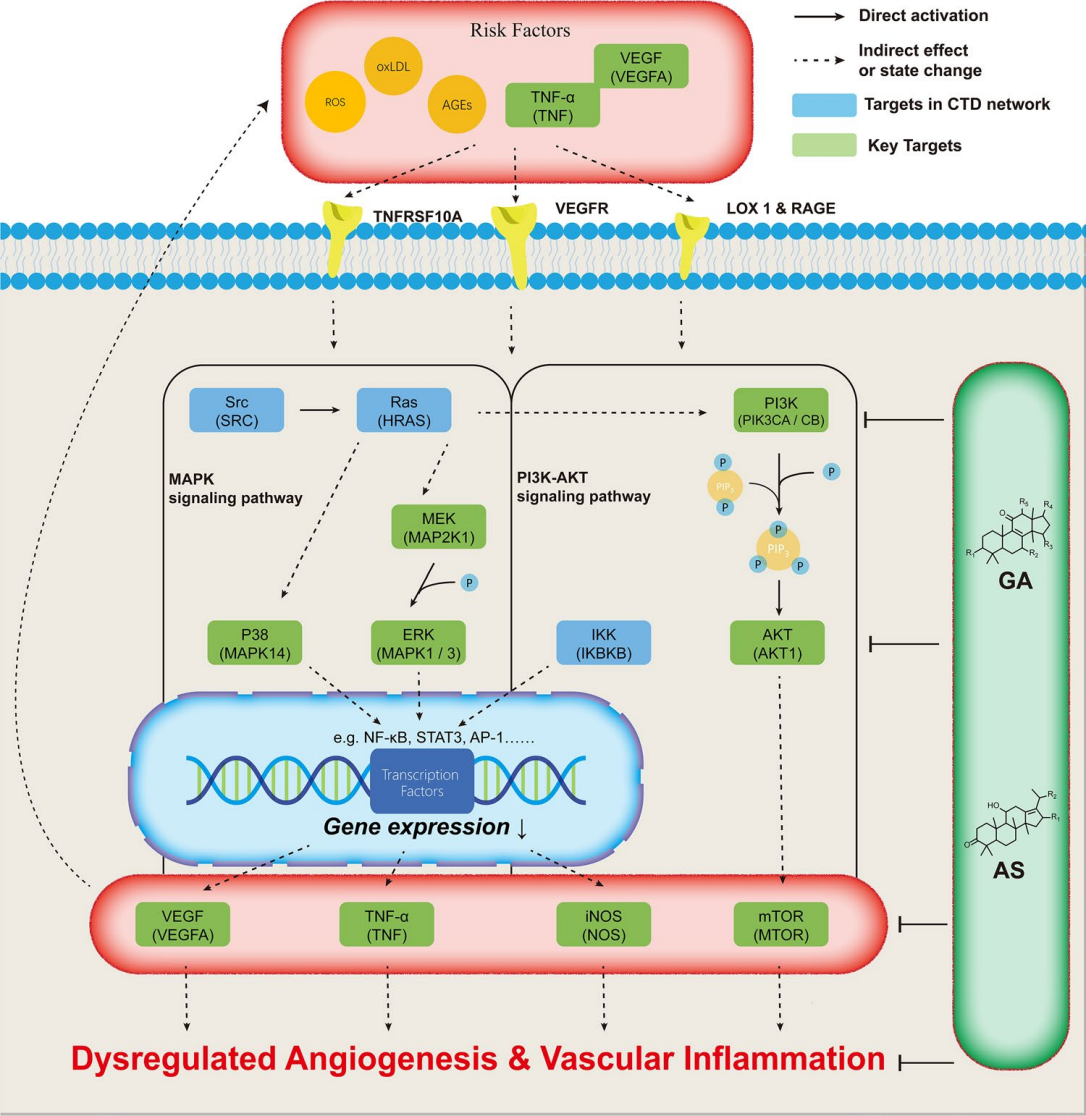
The final mechanism illustration of SQWMG. Combined network pharmacology, molecular docking analysis, and components analysis of the effect of SQWMG on the “MAPK signaling pathway” and “PI3K-Akt signaling pathway” related to RVO.

The result of GO analysis and KEGG pathways analysis revealed the significance of the “PI3K-Akt signaling pathway” and “MAPK signaling pathway”. Following the relation between RVO and vascular dysfunction, we focused on these two pathways and found they shared a big part of the same targets, and the key targets shared by these two pathways also took more than half of the place in the top 10 targets in KEGG pathway analysis (Figure 5d). “PI3K-Akt signaling pathway” and “MAPK signaling pathway” played an important role in the regulation of vascular apoptosis, proliferation, inflammation, and response to environmental change, for example, oxygen and lipid ^31^. Central to the pathogenesis of RVO is the VEFG and related increased dysfunctional proliferation, migration, and inflammation which contained these two signaling pathways^32^. Previous studies had found these two tyrosine kinase pathways underwent upregulation during the pathogenesis of RVO and inhibition of these pathways resulted in a decrease of oxidative and inflammatory stress showing a good effect^32–34^. PI3K-Akt signaling pathways played a multi-functional role in vascular disorder and response to risk factors including VEGF, LDL, and AGEs. In vascular smooth muscle cells, PI3K/Akt activation induced smooth muscle cell proliferation which contributes to a vascular narrowing that could aggravate retinal vein occlusion^35^. Research had also revealed that inhibition of PI3K/Akt-IKK (IKBKB, No. 63 in PPI) could greatly attenuate TNF-α and iNOS^36–38^. PI3k/Akt/mTOR was also proved to be involved in VEGF-induced retinal vascular diseases including RVO and inhibition of mTOR showed great therapeutic effect^39^. When it came to the MAPK signaling pathway, a more complex network was involved. The MAPK cascades included four major MAPKs: the extracellular signal-related kinases (ERK 1/MAPK3, ERK2/MAPK1), the c-Jun N-terminal kinases (MAPK8), the p38 kinase (MAPK14), and the big MAP kinase 1 (ERK 5) which were directly regulated by oxidative stress^40^. The MAPK signaling pathway involved a strong pro-inflammatory effect and production of adhesion molecules by downstream regulation. Once ERK1/2 and p38 were activated by VEGF, TNF-α and endothelial damage, it started signal transduction which eventually phosphorylated the NF-kB p65 subunit and then caused a strong pro-inflammatory effect through upregulation of IL-6, TNF-α, and iNOS as shown in Fig. 8^31,41,42^. PI3K pathway also had close cross-talk with the MAPK pathway and contributed to this pro-inflammatory effect^38,43^. Many studies had found the effect of VEGF and the production of inflammatory factors were dependent on these two pathways, and the main components GA and AS could inhibit this process, thus SQWMG might regulate dysregulated angiogenesis and vascular inflammation by inhibition of these pathways, and eventually remarkably decrease of gene expression of inflammatory factors.

The molecular docking and animal experiment conducted on zebrafish verified this mechanism and involved targets. 20 key protein targets involved in MAPK and PI3K pathways were tested and the results were surprisingly great (Figure 6, Supplementary Table 5). For key targets in MAPK and PI3K signaling pathways including MAPK1 (ERK2), AKT1, TNF-α, iNOS, and mTOR, results showed binding energy below − 10 kcal/mol, revealing their extraordinary affinity to proteins. It also can be seen that components exhibiting extraordinary affinities are almost all GA and AS. AS and GA also had been investigated for their inhibitory effect on PI3K- and MAPK- related targets^26,27,44,45^*.* This result from molecular docking, together with the fact that GA and AS accounted for the largest proportion of the components, suggests that GA and AS are the main components that account for the therapeutic effects of SQWMG. The component that exhibited the highest affinity, LZ-3, was also identified from rat serum after administration of SQWMG, indicating it indeed could be absorbed into the blood to act with these target proteins.

The therapeutic effects of SQWMG on MAPK/PI3K signaling pathways and vascular inflammation were validated on Zebrafish. Zebrafish had a similar requirement on bioactive material as the mammalian angiogenic pathway and its vascular function could be regulated by MAPK signaling according to previous studies^11,46,47^. The results of qPCR validated SQWMG’s effect on these two pathways with repressed gene expression in key targets (Figure 7, Supplementary Figure 4), especially on downstream inflammatory factors. The inhibitory effect on VEGF was amplified through a cascade of signaling and ultimately acted on downstream inflammatory factors. The gene expression levels of iNOS and TNF-α decreased by more than half under the treatment of SQWMG, which was a strong validation of the analysis of the mechanism of SQWMG by network pharmacology and biological enrichment analysis.

Activation of VEGFR by VEGF subsequently started activation of the MAPK pathway as well as activation of PI3K-Akt pathways eventually causing pro-inflammatory factors, indicating the function of VEGF as the target of the existing anti-VEGF treatment. These processes contained classic MAPK pathways and PI3K-Akt pathways with key proteins concluded in the PPI network or CTD network and could be suppressed by the main components, GA and AS in SQWMG. The result from the network and biological enrichment analysis was verified in molecular docking and qPCR, and the main components were validated by analysis of rat serum, revealing the pharmacological mechanism and substance basis of SQWMG as shown in Fig. 8. Although this study had primarily demonstrated the components and mechanism of SQWMG, several limitations need to be acknowledged. The targets and components in the CTD network were enormous and we focused on those that showed great properties in biological enrichment. Therefore, the isolated targets were not included in further analysis though they may also play crucial roles. Another limitation is the lack of a disease animal model to perform more accurate validation which shall be improved in the following research.

## Conclusion

In summary, we employed UPLC-Triple-TOF/MS and network pharmacology investigation in this study to analyze the substance basis and the complex pharmacological activity of SQWMG on RVO treatment. UPLC-Triple-TOF/MS identified 63 components with GA and AS being the main components. A final CTD network containing 169 genes and 68 components (plus 5 isomers) showed the characteristics of multi-target, multi-pathway, and multi-mechanism. Further analysis of the involved targets revealed the close relation between RVO and vascular risk factors including VEGFA and the crucial role of MAPK and PI3K-Akt signaling pathways through their impact on vascular inflammation. Finally, the results of network pharmacology were validated by molecular docking and qPCR experiment. Based on our findings from previous and current studies, we clarified that SQWMG could alleviate the inflammation and subsequently improve retinal blood supply and vascular obstruction through the regulation of MAPK and PI3K-Akt signaling pathways and downstream iNOS and TNF-α. Main components, including ganoderic acid B, were also identified from rat serum after treatment. Overall, the results provided a novel, theoretical insight into understanding the bioactive components and the pharmacological mechanism of SQWMG. It was also known from the analysis of molecular docking results that the GA and AS played a major role in SQWMG so this study also provided a basis for reducing the prescription of SQWMG. More and further research could be conducted following the CTD network and the above analysis.

## Methods

### Experimental drugs and reagents

SQWMG was provided by Shanghai Liantang Pharmaceutical Co., Ltd (Lot No. 20200902). Acetonitrile and methanol for MS analysis were purchased from E. Merck Chemical Co. Ltd. (MS pure, Lot No. I1133829105 and I1139035113). Formic acid was purchased from CNW Technologies (MS pure, Lot No. Y6170039). The zebrafish embryos were anesthetized with tricaine (ethyl 3-aminobenzoate methane sulfonate, MS-222 (Sigma-Aldrich In., St. Louis, MO, USA) final concentration of 0.016%). 1-phenyl-2-thiourea (PTU) was purchased from Sigma-Aldrich Co (St. Louis, MO, USA). TRIzol reagent (10,296,028, Thermo Fisher Scientific, Waltham, MA, USA), Hifair II 1st Strand cDNA Synthesis SuperMix reagent Kit with gDNA digester (11123ES60, Yeasen, Shanghai, China), Hifair qPCR SYBR Green Master Mix (High Rox Plus) (11203ES03 Yeasen, Shanghai) was used for qPCR analysis.

### The clinical application of SQWMG

Data from the medical records of patients prescribed SQWMG in the outpatient ophthalmology clinic of Shanghai General Hospital in the previous six months (2022/6/1-2022/11/31) were retrospectively collected and analyzed in age, gender, disease, and re-prescription rate.

### Analysis of SQWMG components through UPLC-Triple-TOF/MS

#### Preparation of SQWMG sample

1.0 g of SQWMG was dissolved in 10 mL of 20% (v/v) methanol-water, sonicated (250 W, f = 40 kHz) for 30 minutes, and then cooled to room temperature. Next, 2 mL of cooled liquid was centrifuged (12000 RPM, 5 minutes) to obtain the final sample solution from the supernatant. For more specific analysis, collected 1 mL of each aqueous extract of Rehmanniae Radix Praeparata, Fructus Ligustri Lucidi, Angelicae Sinensis Radix, Alisma Orientale (Sam.) Juz., Carthami Flos, Radix Paeoniae Rubra, Paeoniae Radix Alba, and Plantaginis Herba, and diluted 10 times with 10 mL of 20% (v/v) methanol-water. Then the aqueous extracts were centrifuged (12000 RPM, 5 minutes) to obtain the final sample solution from the supernatant. The same process was repeated with 2 mL of Poria Cocos (Schw.) Wolf. aqueous extract. Finally, aqueous extract collected from Codonopsis Radix, Lycii Fructus, and Ganoderma Lucidum was diluted with 10 mL of 20% (v/v) methanol-water.

#### UPLC-Triple-TOF/MS conditions

Each sample was analyzed on a Sciex Triple TOF 4600 LC-MS equipped with an Agilent UPLC column (Agilent ZORBAX RRHD SB-Aq, 2.1×100 mm, 1.8 µm). The mobile phases consisted of (A) acetonitrile and (B) 0.1% formic acid aqueous solution. The gradient elution program was optimized as shown in Supplementary Table 7. The injection volume was 2 μL with a detection wavelength of 190~400 nm. Mass spectrometric data were obtained by Sciex Triple TOF 4600 LC-MS equipped with a Dual Agilent Jet Stream electrospray ionization (ESI) source. The ESI source was set both in positive and negative ionization modes. The parameters in the source were set as shown in Supplementary Table 7.

#### Component identification

Data were acquired by the software of Analyst TF 1.7.1 and analyzed on Peakview 1.2. The MS and MS/MS data were matched with the database of Natural Products HR-MS/MS Spectral Library 1.0. The database of Natural Products HR-MS/MS Spectral Library 1.0 recorded multistage mass spectrograms of standard substances including different acquisition modes, different adduct ions, different collision energies, etc. Then, the components were further screened according to the score of the chromatographic peak. Finally, the components were identified from relative information of MS and MS/MS. The components which could not be found in the database were identified from a fragmental pattern or relative literature.

### Identification of potential target of components

The potential targets of these components were obtained from the website of SwissTargetPrediction (http://www.swisstargetprediction.ch/)^48^. Species were set to “Homo sapiens”. Only genes with a high possibility (probability score > 0) were selected as potential targets of drug components. Then RVO disease-related targets were collected from three sources: GeneCards (http://www.genecards.org/, search date: October 10th, 2021), DisGeNET (https://www.disgenet.org/, search date: October 10th, 2021), and OMIM (https://omim.org, search date: October 10th, 2021)^49^. Only proteins from ‘Homo sapiens’ were selected and included all types of RVO. Then targets shared by drug and disease were retained using “VENNY 2.1” while the other targets were removed.

### Networks construction and analysis

Drug-Components-Target network, Component-target-disease (CTD) network, protein-protein interaction (PPI) network, and potential target gene-related pathway network were constructed by Cytoscape (ver.3.9.0). Nodes in the networks represent chemicals, diseases or targets, and edges indicate interactions between two nodes. For the topological properties, “degree” (D), “closeness centrality” (CC), and “betweenness centrality” (BC) were calculated to demonstrate the further analysis. Shared targets by drug and disease were performed by a PPI analysis by String database (https://string-db.org/). Kyoto encyclopedia of genes and genomes (KEGG) enrichment analysis and gene ontology biological process (GO-BP), cellular component (GO-CC), and molecular function (GO-MF) were carried out using the Database of Metascape (https://metascape.org/)^50^. The p-value is calculated by the hypergeometric test as the probability of obtaining overlapping genes of certain pathway members, forming a cumulative hypergeometric distribution.

### Molecular docking of key ingredients to key targets

AutoDock Vina (ver.1.1.2) was used for ingredient-target molecular docking^51,52^. The exhaustiveness of the global search was set to 20 to obtain a better result^53^. The maximum number of binding modes was 3. The maximum energy difference between modes was 3 kcal/mol. Each pair was measured five times in parallel and the highest value was averaged as the final score. The designed ligands in the protein PDB file are preferentially selected as the control group. The information on target proteins and ligands was shown in Supplementary Table 6. The scoring function weights and terms were set as default. The analysis of interaction was conducted on ProteinsPlus (https://proteins.plus).

### Animals

Larvae zebrafish were maintained, handled, and bred according to standard protocols from the Institutional Animal Care Committee of Shanghai Jiao Tong University. The transgenic zebrafish Tg (kdrl: GFP) are tagged with green fluorescent protein on vascular endothelial cells. Pathogen-free male SD rats (200 g) were provided by Shanghai Silaike experimental animal Co., Ltd. (Shanghai, China, approval number: 2021-0008). Rats were housed in an animal room (24 ± 2 °C, 60 ± 5% relative humidity) with the setting of a 12 h dark/light cycle. Animal studies were performed in accordance with the Guide for the Care and Use of Laboratory Animals, the guidelines of ARRIVE (Animal Research: Reporting of In Vivo Experiments), and relevant Chinese laws and regulations, and approved by the Institutional Animal Care and Use Committee of Shanghai Jiao Tong University (approval number A2018075).

### Zebrafish maintenance and embryo handling

After normal fertilization, embryos were collected and kept at 28.5 °C in E3 medium (0.17 mM KCl, 5 mM NaCl, 0.33 mM CaCl_2_, and 0.33 mM MgSO_4_) and staged by hours post-fertilization (hpf) and days post-fertilization (pdf). At 24 hpf, the chorion of embryos was removed by a protease (Sigma-Aldrich Inc., St. Louis, MO, USA). Then the embryos were randomly assigned to 24-well culture plates with fifteen embryos per well in E3 medium plus 0.003% PTU to prevent pigmentation. These embryos were divided into three groups: blank control group, positive control group (Pancreatic Kininogenase, 1mg/mL), and SQWMG group (1, 4μg/mL). The medium solution was changed every other day.

### Key targets analysis through qPCR

After drug treatment for 4dpf, total RNA was extracted from Zebrafish embryos with TRIzol reagent. cDNAs were synthesized from total RNA using the Hifair II 1st Strand cDNA Synthesis SuperMix reagent Kit with a gDNA digester. Hifair qPCR SYBR Green Master Mix (High Rox Plus) was used for qPCR analysis. The qPCR was done in an ABI HT-7900 machine (Thermo Fisher Scientific, Waltham, MA, USA). Results were calculated using the 2−ΔΔCT method with WT embryos as the control^54^. The primers for different target genes and the reference gene (β-actin) are listed in Supplementary Table 7.

### Key components analysis in rat serum

#### Drug administration

Before the experiment, rats were given water and fed standard laboratory food for acclimatization for a week. SQWMG 0.72 ml (dissolved in purified water, 0.5 g/ml) was orally administrated to the rats twice a day at the dose of 3.6 g/kg (dose volume/body weight) for seven days. The blood collection time is 0.5 h, 1.5 h, and 5 h after the last dosing.

#### Plasma collection and preparation

The blood samples were drawn into an Eppendorf tube and then centrifuged at 6500 rpm for 10 min at 4 °C. Then, the plasma samples were transferred to Eppendorf tubes and stored at − 80 °C for further analysis. 200 μL plasma was treated with 600 μL of methanol to precipitated protein, then vortex mixed for 5 min at 4 °C and centrifuged at 12000 rpm for 15 min at 4 °C. The supernatant was transferred to another tube for further analysis.

#### UPLC-Triple-TOF/MS conditions and component identification

The UPLC-Triple-TOF/MS conditions and the process of data analysis were the same as in the analysis of SQWMG components as shown in Supplementary Table 7.

### Statistical analysis

All the data have been expressed as mean ± SD. GraphPad Prism (version 8; GraphPad Software, La Jolla, CA) was used to analyze and illustrate the results. The statistical significance is described in the respective figure legends (*P < 0.05, **P < 0.01, ***P < 0.001, ****P < 0.0001, One-way ANOVA).

## Supplementary Information

Supplementary Information 1.

Supplementary Figure 1.

Supplementary Figure 2.

Supplementary Figure 3.

Supplementary Figure 4.

Supplementary Figure 5.

Supplementary Table 1.

Supplementary Table 2.

Supplementary Table 3.

Supplementary Table 4.

Supplementary Table 5.

Supplementary Table 6.

Supplementary Table 7.

## Data Availability

The datasets used and analyzed during the current study are available from the corresponding author upon reasonable request.
