# Supplementary material for: Exploring the components and mechanisms of Shen-qi-wang-mo granule in the treatment of retinal vein occlusion by UPLC-Triple TOF MS/MS and network pharmacology
Source: Sci Rep. 2023 Apr 1;13:5330. doi: 10.1038/s41598-023-32472-0 (PMC10066998; doi:10.1038/s41598-023-32472-0)
Supplement: Supplementary file 1 — Supplementary Information 1. [file 41598_2023_32472_MOESM1_ESM.docx]

# Supplementary Information

## Supplementary Figure

- Supplementary Figure 1

Statistics of outpatient prescriptions of SQWMG in Shanghai General Hospital in recent six months

- Supplementary Figure 2

MS spectrometry of all 63 compounds

- Supplementary Figure 3

Interaction analysis of bindings with high affinity

- Supplementary Figure 4

qPCR results

- Supplementary Figure 5

Base peak intensity chromatogram of serum

## Supplementary Table

- Supplementary Table 1

Targets information

- Supplementary Table 2

CTD network analysis

- Supplementary Table 3

PPI analysis

- Supplementary Table 4

Biological enrichment

- Supplementary Table 5

Docking results

- Supplementary Table 6

qPCR results

- Supplementary Table 7

Experiment parameters
