# Supplementary figures and images for: Exploring the components and mechanisms of Shen-qi-wang-mo granule in the treatment of retinal vein occlusion by UPLC-Triple TOF MS/MS and network pharmacology
Source: Sci Rep. 2023 Apr 1;13:5330. doi: 10.1038/s41598-023-32472-0 (PMC10066998; doi:10.1038/s41598-023-32472-0)

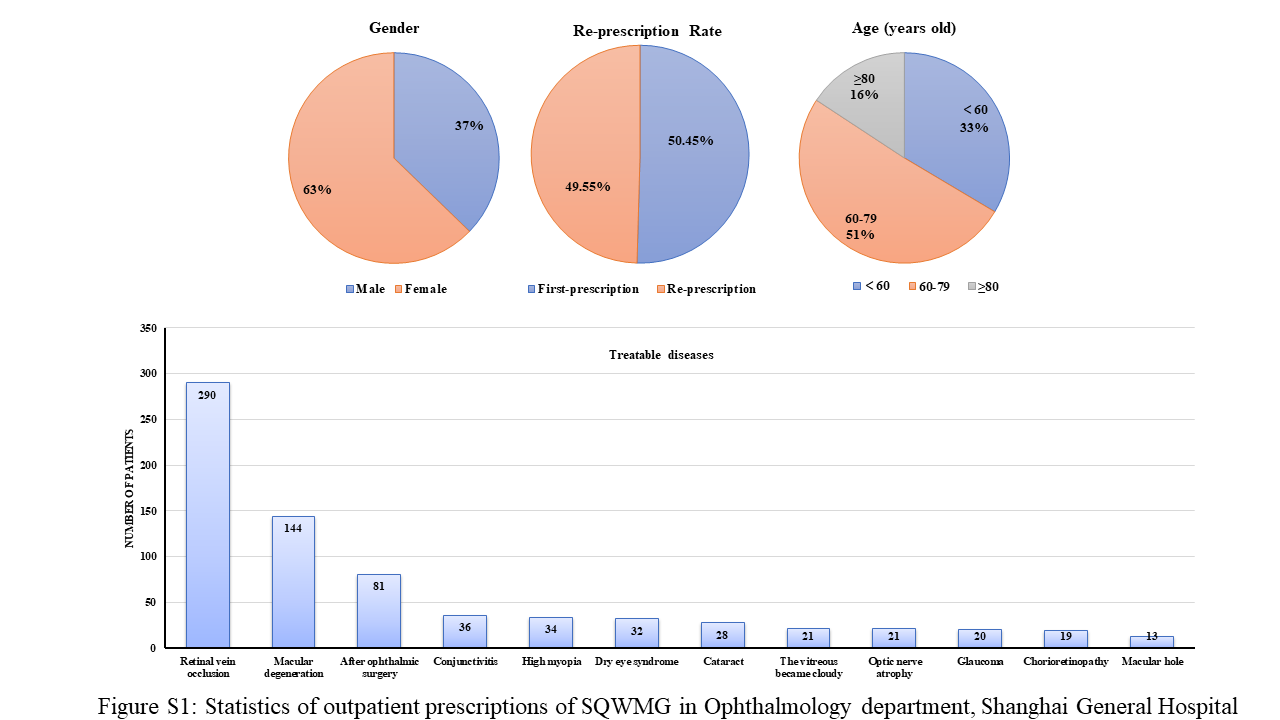

Supplement: Supplementary file 2 — Supplementary Figure 1. [file 41598_2023_32472_MOESM2_ESM.png]

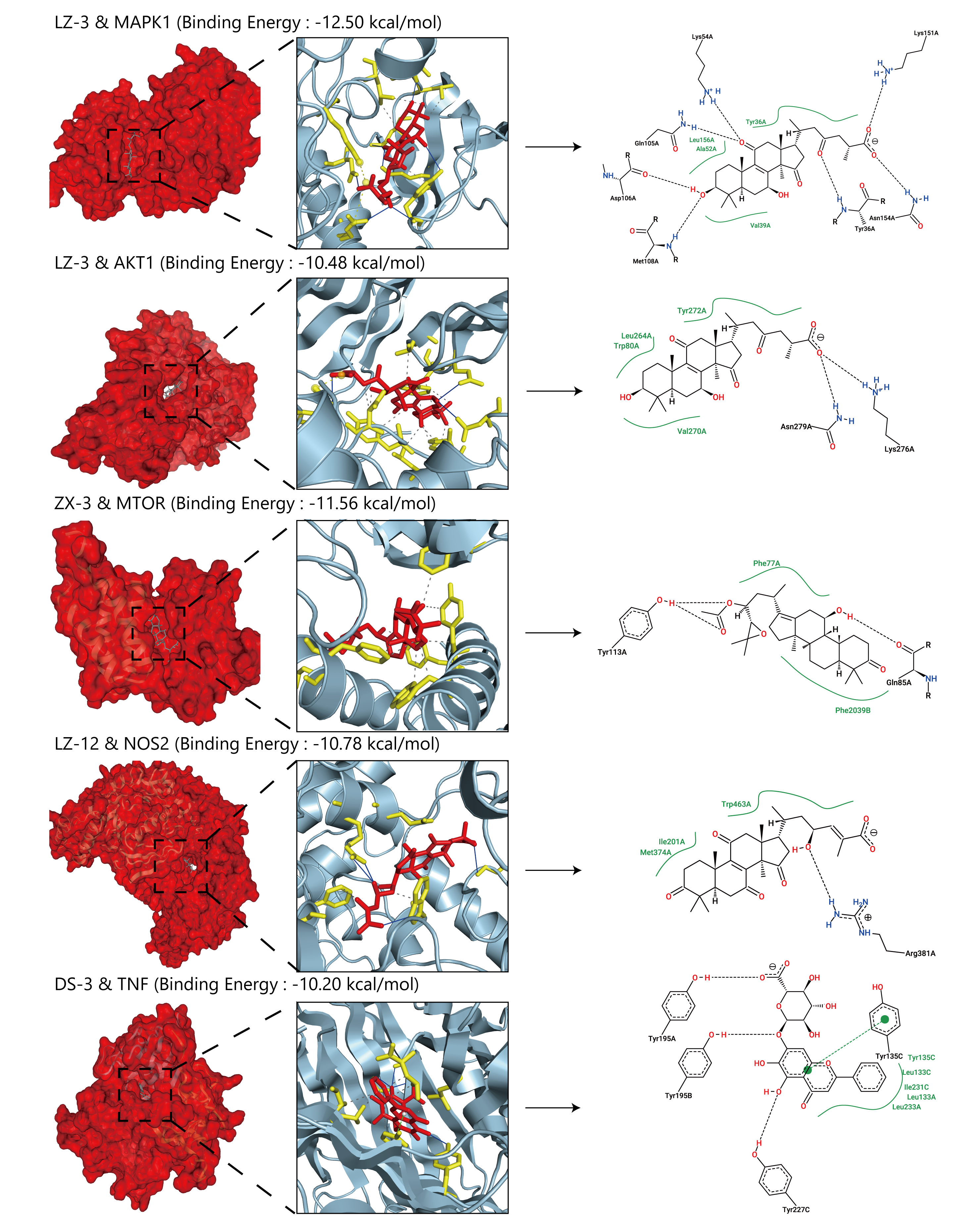

Supplement: Supplementary file 4 — Supplementary Figure 3. [file 41598_2023_32472_MOESM4_ESM.png]

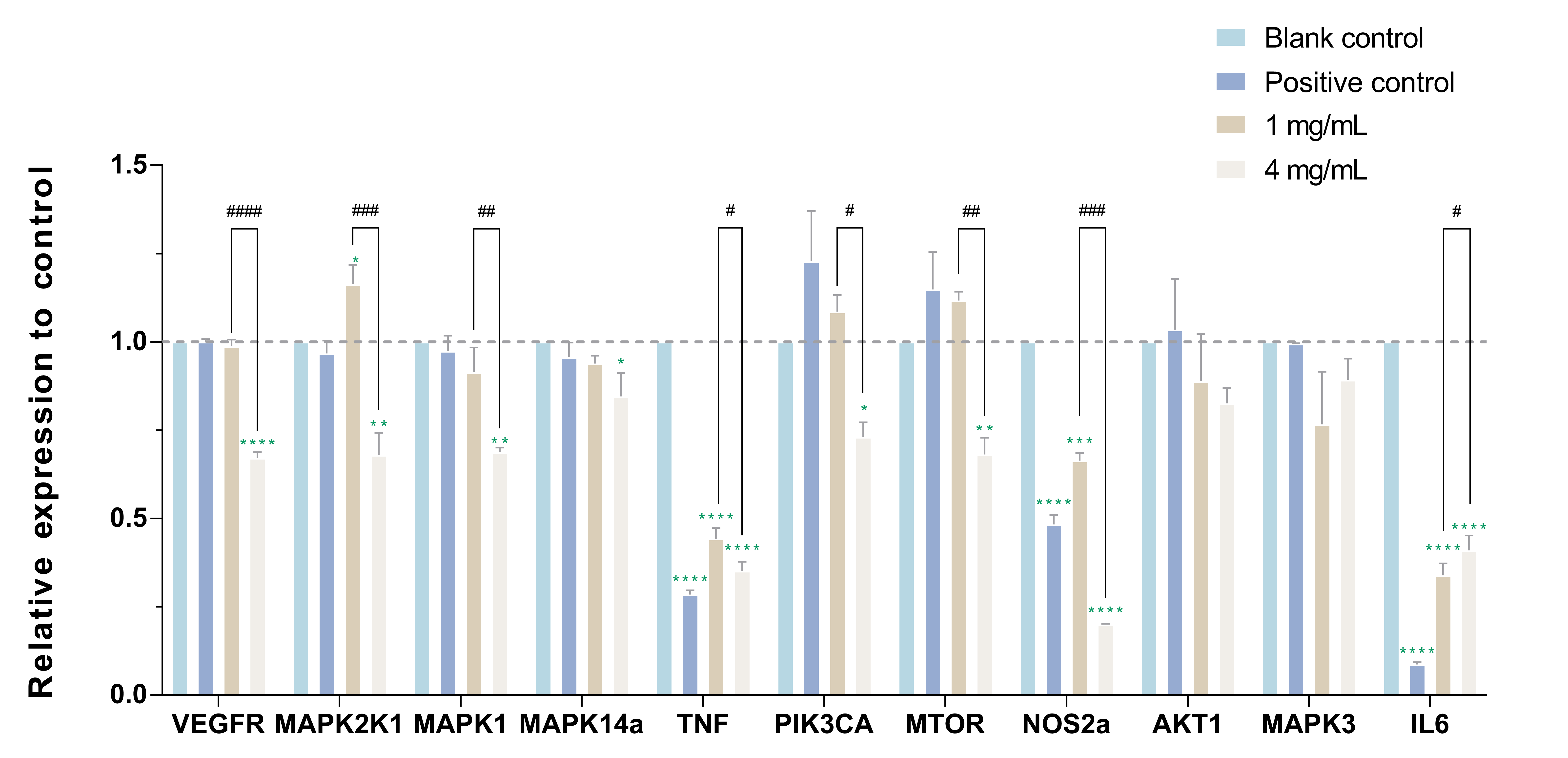

Supplement: Supplementary file 5 — Supplementary Figure 4. [file 41598_2023_32472_MOESM5_ESM.tif]

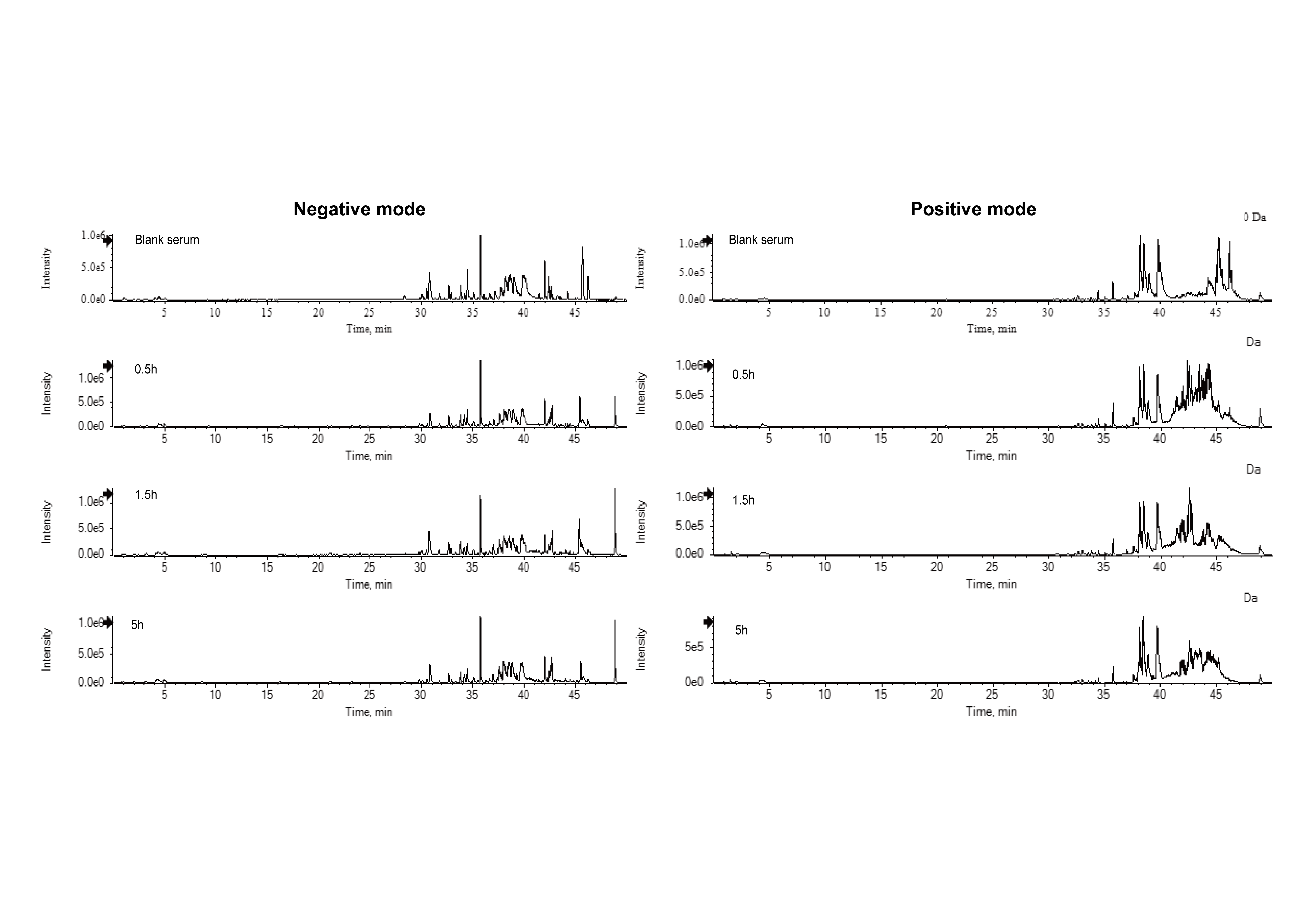

Supplement: Supplementary file 6 — Supplementary Figure 5. [file 41598_2023_32472_MOESM6_ESM.png]
