# Supplementary material for: Exploring the components and mechanisms of Shen-qi-wang-mo granule in the treatment of retinal vein occlusion by UPLC-Triple TOF MS/MS and network pharmacology
Source: Sci Rep. 2023 Apr 1;13:5330. doi: 10.1038/s41598-023-32472-0 (PMC10066998; doi:10.1038/s41598-023-32472-0)

- MS^1/MS^2 spectrum of No.1, negative

Spectrum from 20221014_TS22C148-SQWMKL_neg.w...iment 1, -TOF MS (50 - 1700) from 2.179 min

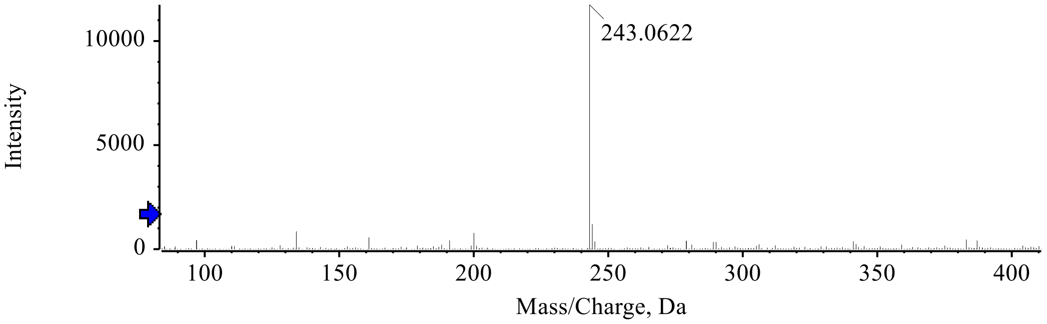

Spectrum from 20221014_TS22C148-SQWMKL_neg.w...ent 3, -TOF MS^2 (50 - 1250) from 2.132 min
Precursor: 243.1 Da

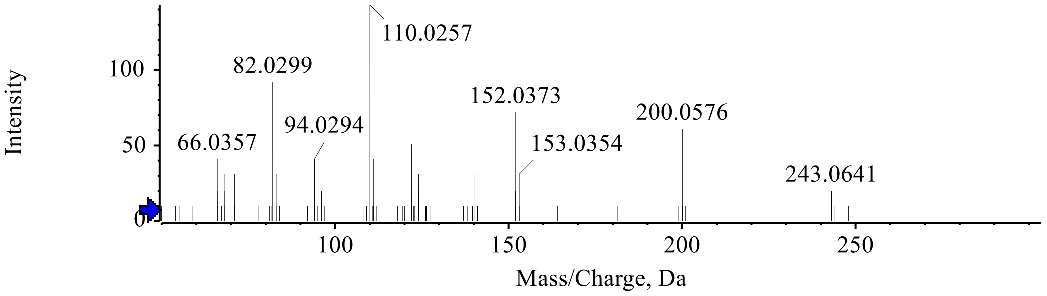

- MS^1/MS^2 spectrum of No.2, negative

Spectrum from 20221014_TS22C148-SQWMKL_neg.w...iment 1, -TOF MS (50 - 1700) from 2.280 min

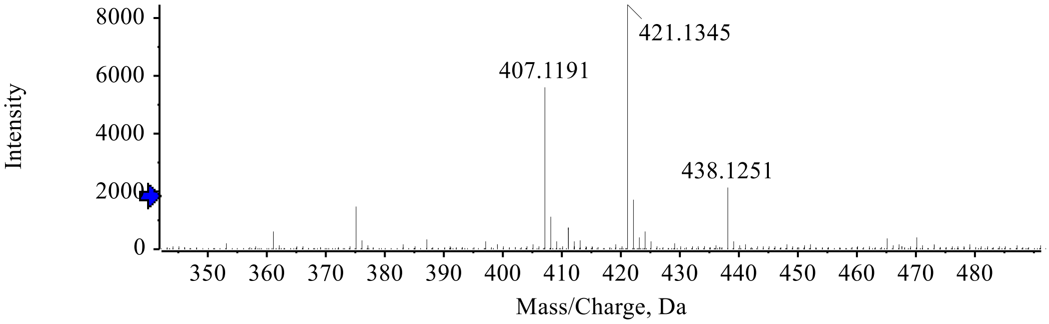

Spectrum from 20221014_TS22C148-SQWMKL_neg.w...ent 4, -TOF MS^2 (50 - 1250) from 2.261 min
Precursor: 407.1 Da

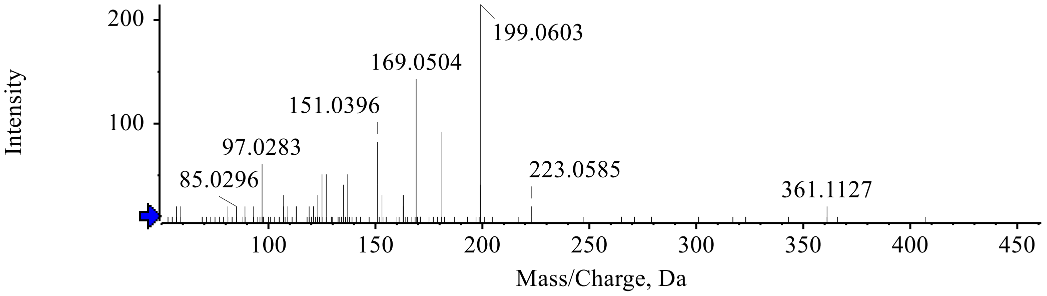

- MS^1/MS^2 spectrum of No.3, negative

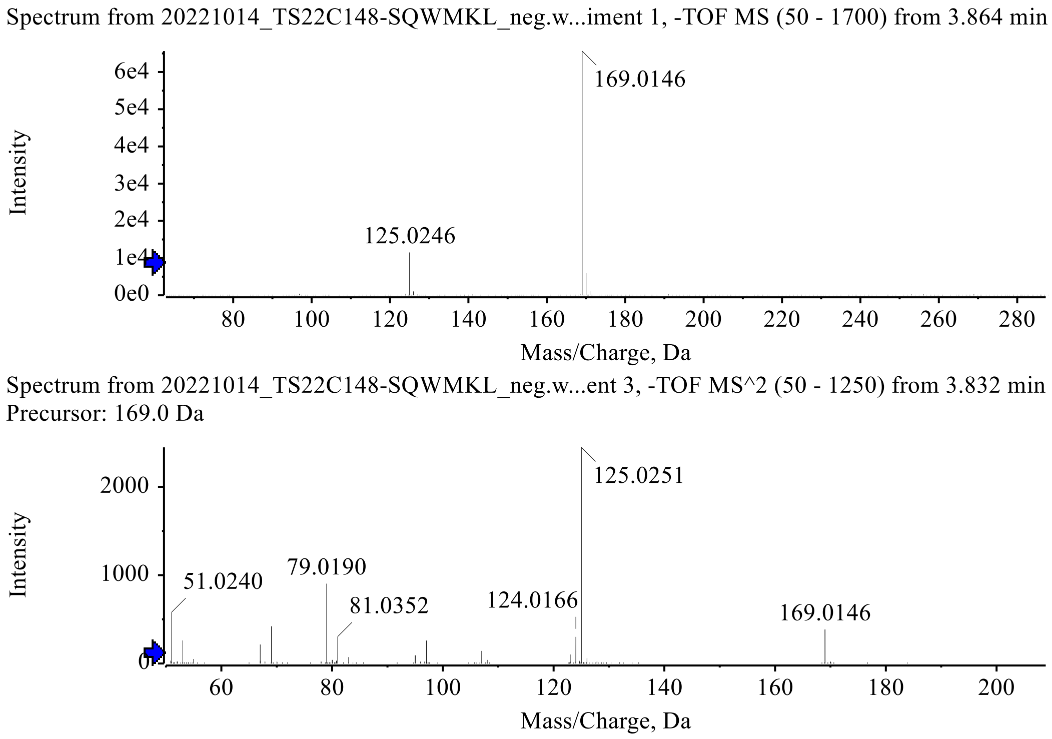

● MS^1/MS^2 spectrum of No.4, negative

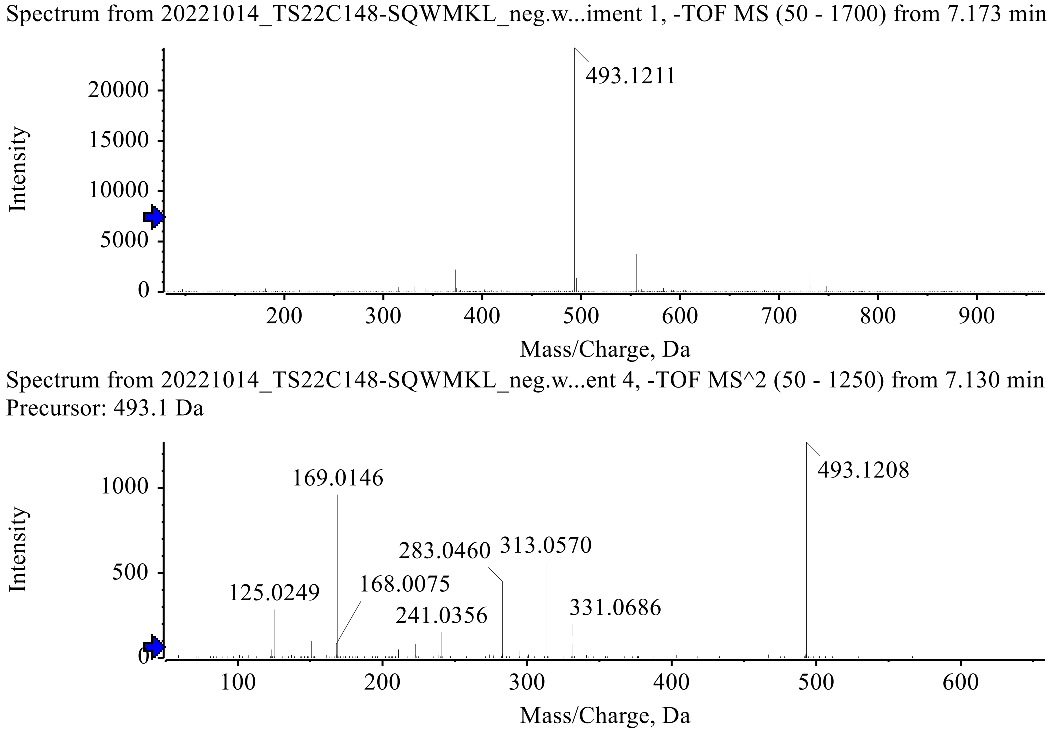

● MS^1/MS^2 spectrum of No.5, negative

Spectrum from 20221014_TS22C148-SQWMKL_neg.w...iment 1, -TOF MS (50 - 1700) from 7.249 min

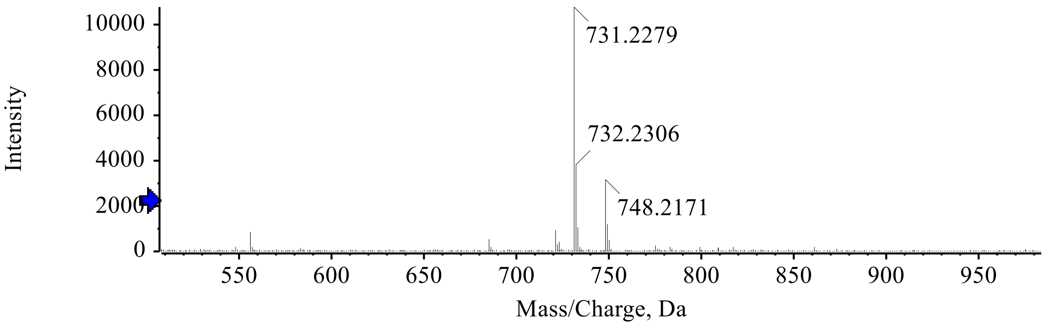

Spectrum from 20221014_TS22C148-SQWMKL_neg.w...ent 3, -TOF MS^2 (50 - 1250) from 7.182 min
Precursor: 731.2 Da

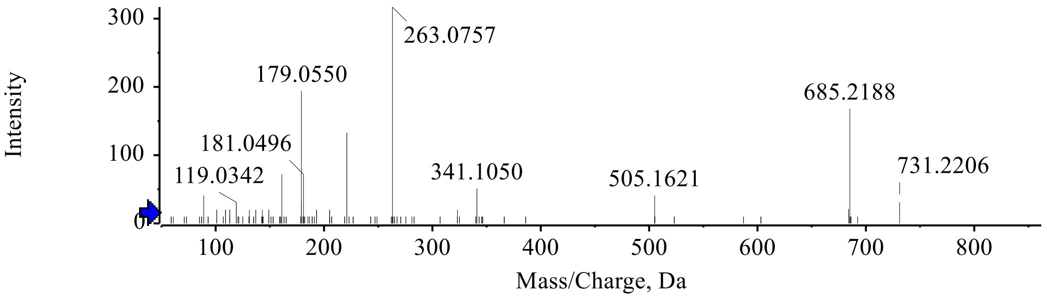

● MS^1/MS^2 spectrum of No.6, negative

Spectrum from 20221014_TS22C148-SQWMKL_neg.w...iment 1, -TOF MS (50 - 1700) from 9.318 min

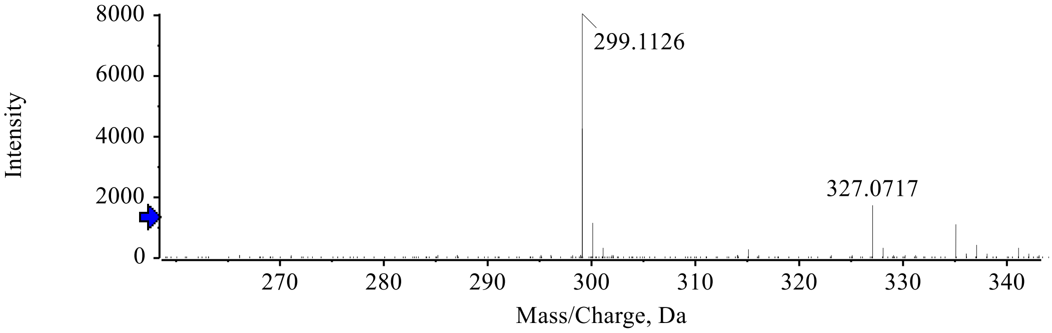

Spectrum from 20221014_TS22C148-SQWMKL_neg.w...ent 2, -TOF MS^2 (50 - 1250) from 9.279 min
Precursor: 299.1 Da

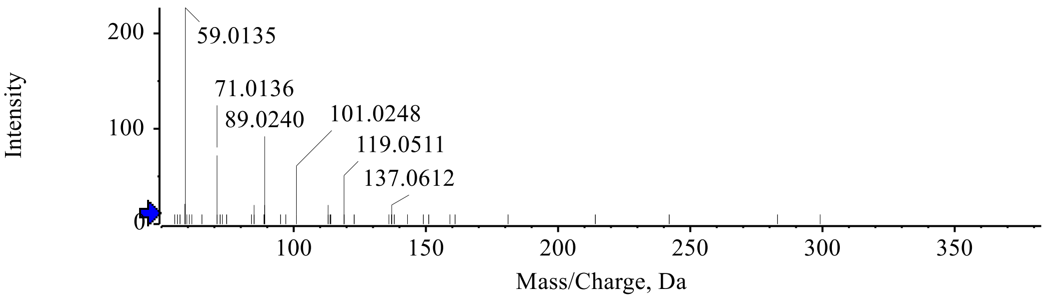

● MS^1/MS^2 spectrum of No.7, negative

Spectrum from 20221014_TS22C148-SQWMKL_neg.w...ment 1, -TOF MS (50 - 1700) from 10.196 min

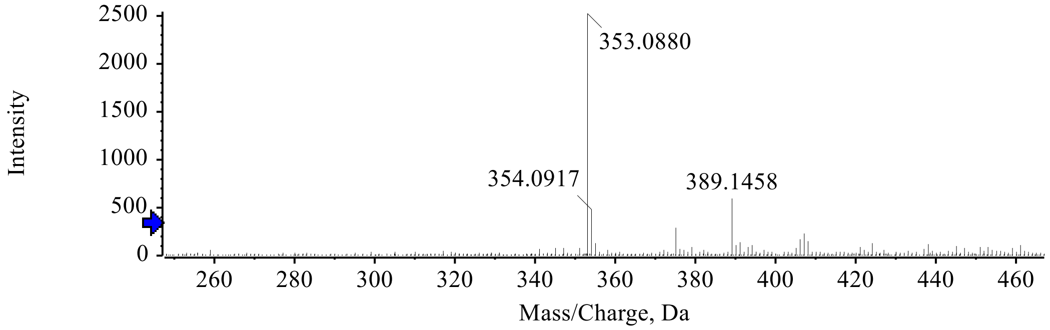

Spectrum from 20221014_TS22C148-SQWMKL_neg.w...nt 2, -TOF MS^2 (50 - 1250) from 10.149 min
Precursor: 353.1 Da

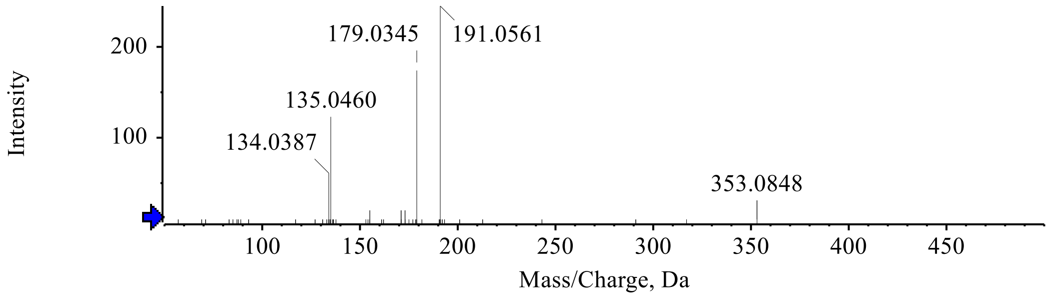

- MS[1]/MS[2] spectrum of No.8, negative

Spectrum from 20221014_TS22C148-SQWMKL_neg.w...ment 1, -TOF MS (50 - 1700) from 11.458 min

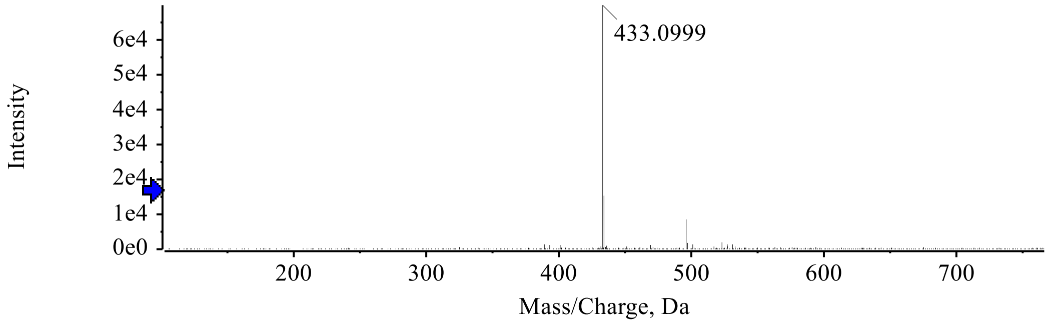

Spectrum from 20221014_TS22C148-SQWMKL_neg.w...nt 2, -TOF MS^2 (50 - 1250) from 11.413 min
Precursor: 433.1 Da

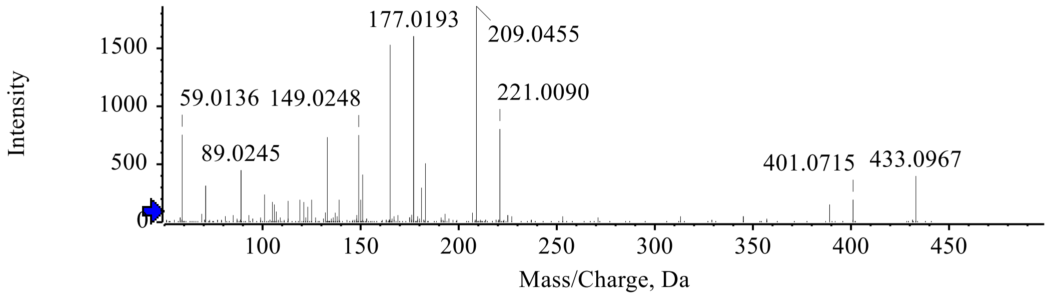

- MS[1]/MS[2] spectrum of No.9, negative

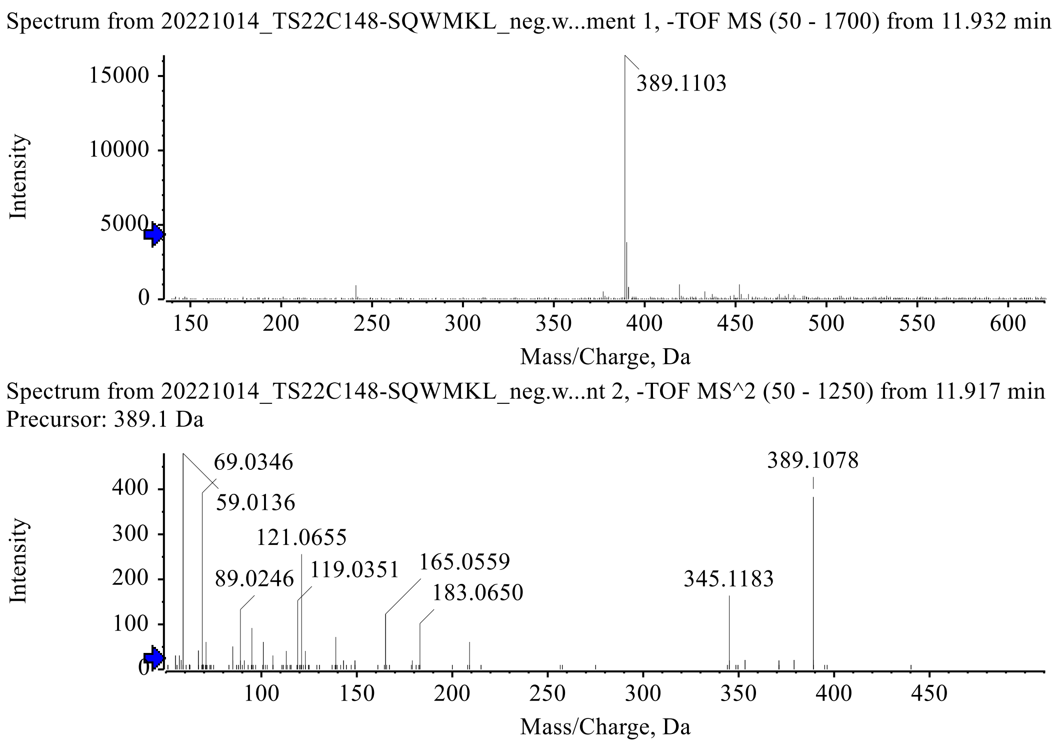

● MS[1]/MS[2] spectrum of No.10, negative

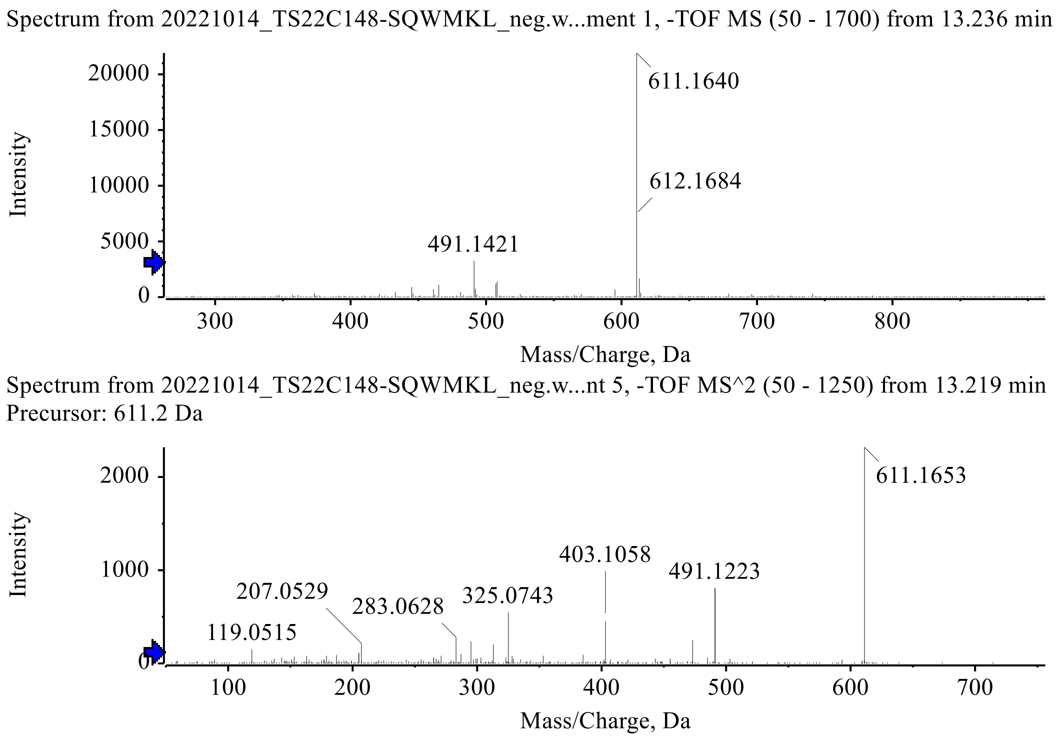

● MS[1]/MS[2] spectrum of No.11, positive

Spectrum from 20221014_TS22C148-SQWMKL_pos....ment 1, +TOF MS (50 - 1700) from 13.647 min

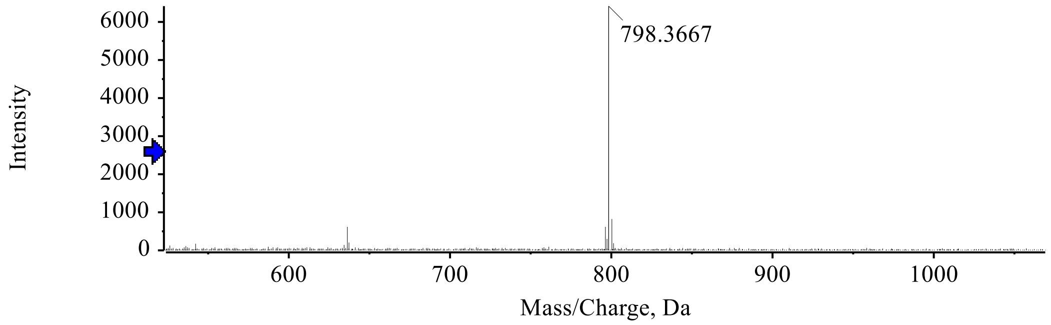

Spectrum from 20221014_TS22C148-SQWMKL_pos.w...nt 5, +TOF MS^2 (50 - 1250) from 13.604 min
Precursor: 798.4 Da, CE: 40.0

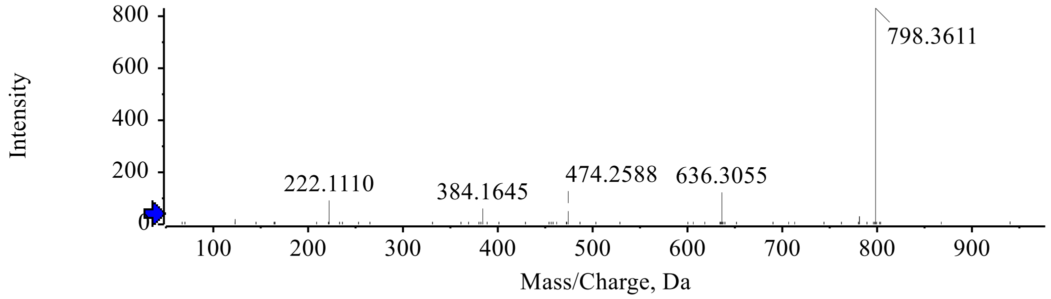

● MS^1/MS^2 spectrum of No.12, positive

Spectrum from 20221014_TS22C148-SQWMKL_pos....ment 1, +TOF MS (50 - 1700) from 14.153 min

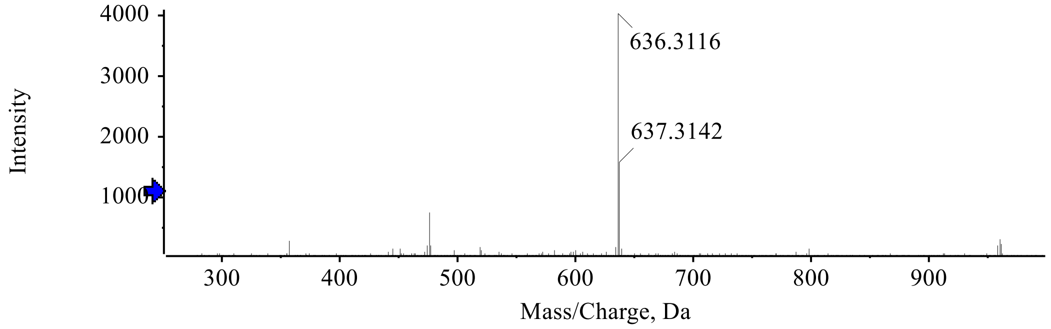

Spectrum from 20221014_TS22C148-SQWMKL_pos.w...nt 5, +TOF MS^2 (50 - 1250) from 14.094 min
Precursor: 636.3 Da, CE: 40.0

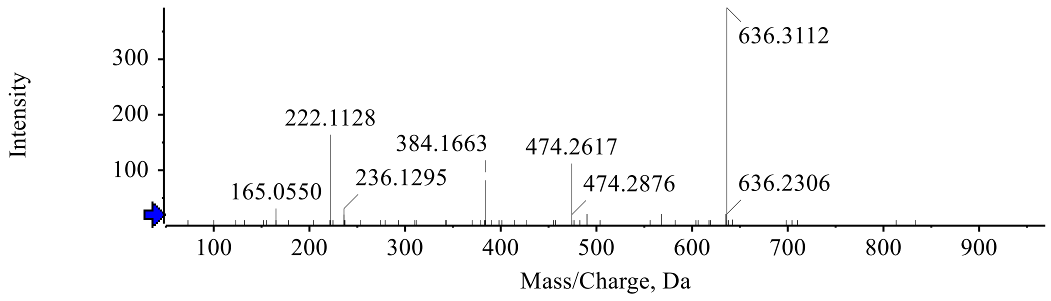

● MS^1/MS^2 spectrum of No.13, negative

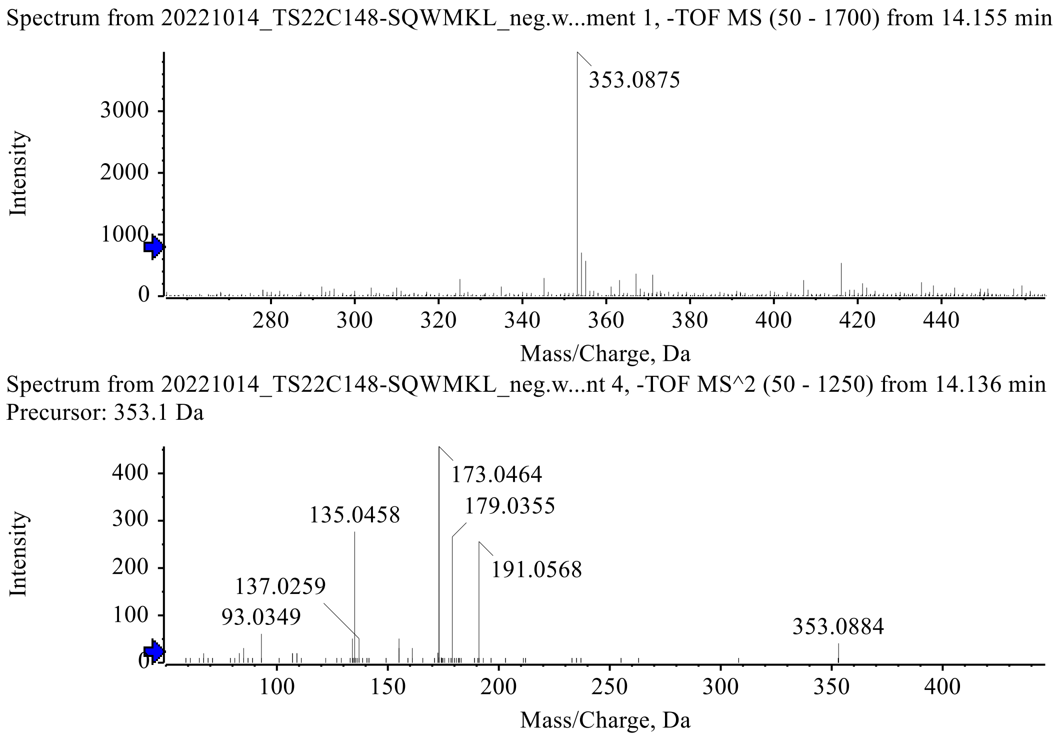

- MS^1/MS^2 spectrum of No.14, negative

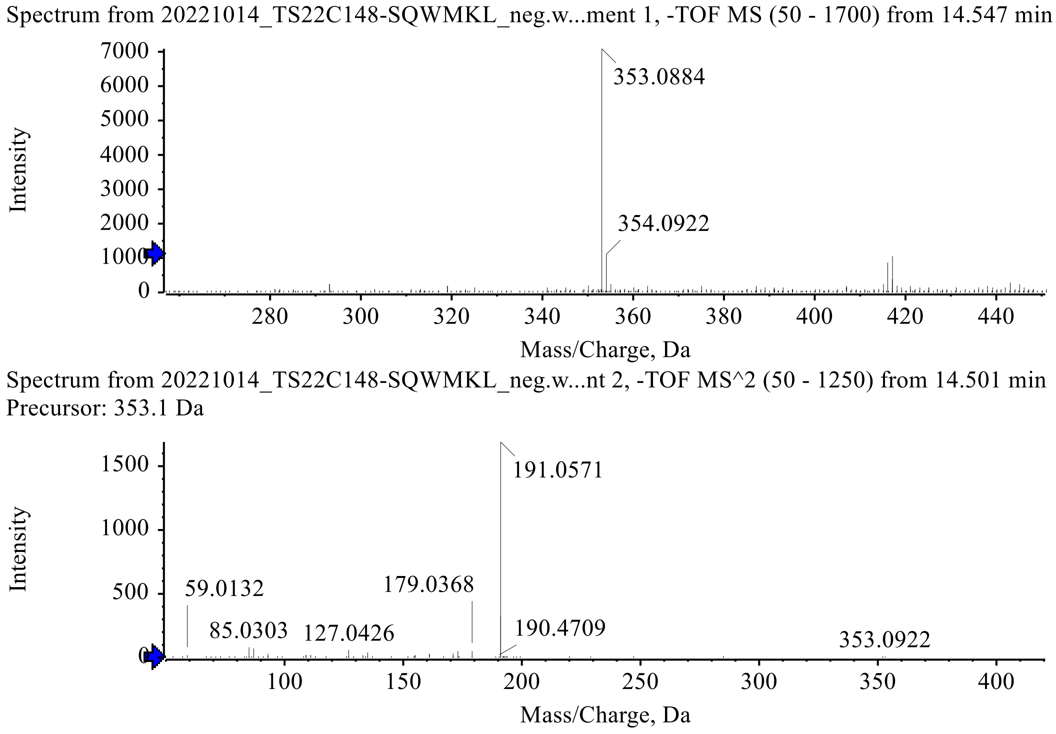

- MS^1/MS^2 spectrum of No.15, negative

Spectrum from 20221014_TS22C148-SQWMKL_neg.w...ment 1, -TOF MS (50 - 1700) from 15.098 min

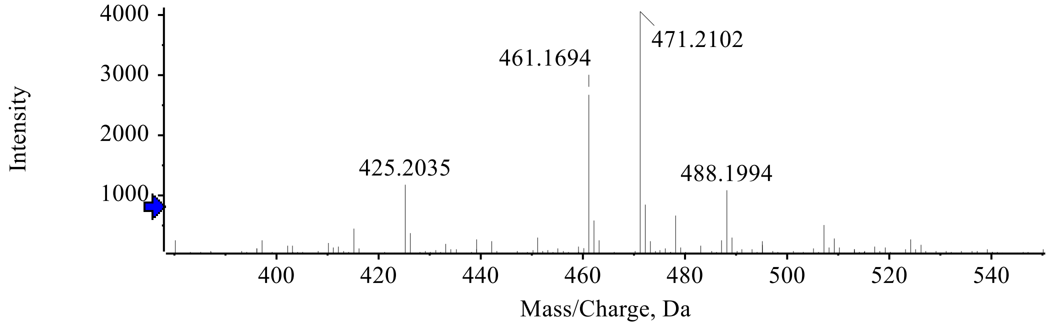

Spectrum from 20221014_TS22C148-SQWMKL_neg.w...nt 3, -TOF MS^2 (50 - 1250) from 15.040 min
Precursor: 471.2 Da

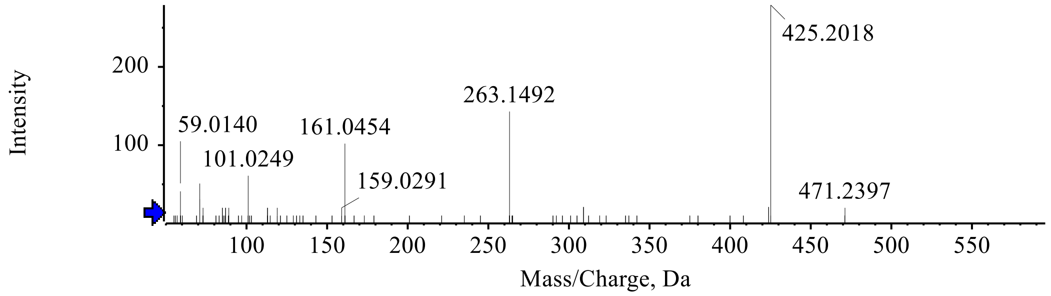

- MS[1]/MS[2] spectrum of No.16, positive

Spectrum from 20221014_TS22C148-SQWMKL_pos....ment 1, +TOF MS (50 - 1700) from 15.106 min

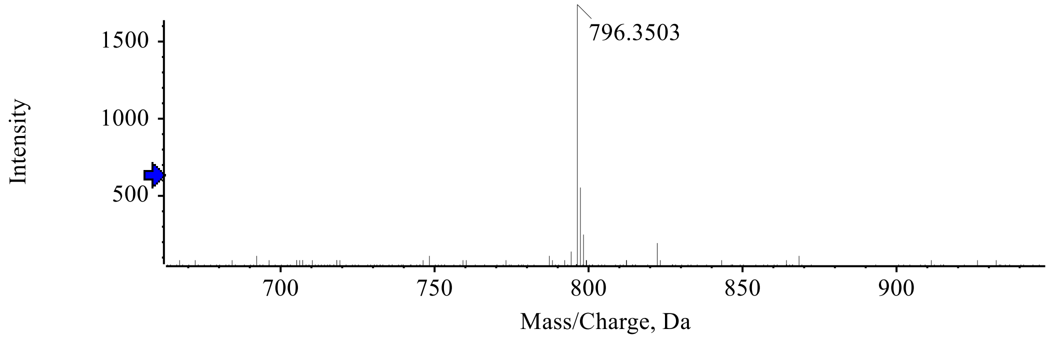

Spectrum from 20221014_TS22C148-SQWMKL_pos.w...nt 3, +TOF MS^2 (50 - 1250) from 15.077 min
Precursor: 796.4 Da, CE: 40.0

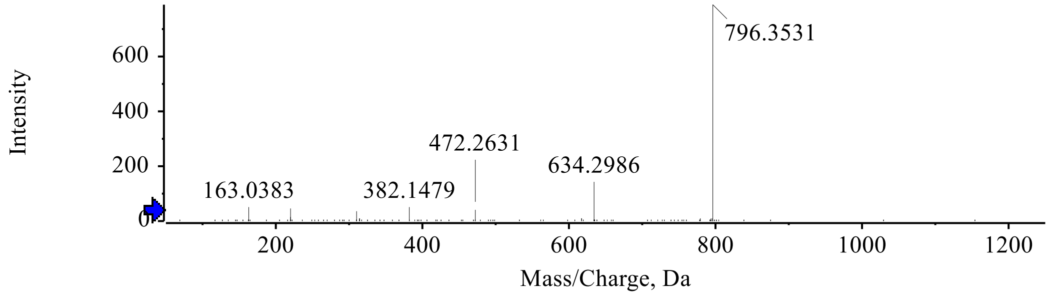

- MS[1]/MS[2] spectrum of No.17, negative

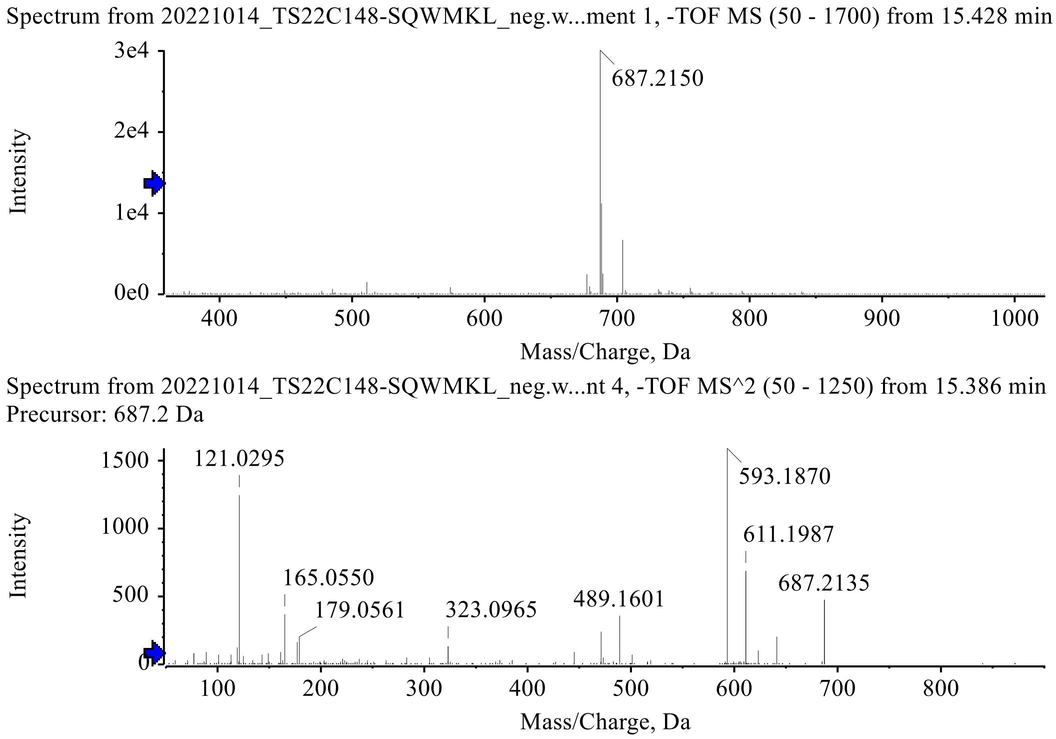

● MS^1/MS^2 spectrum of No.18, negative

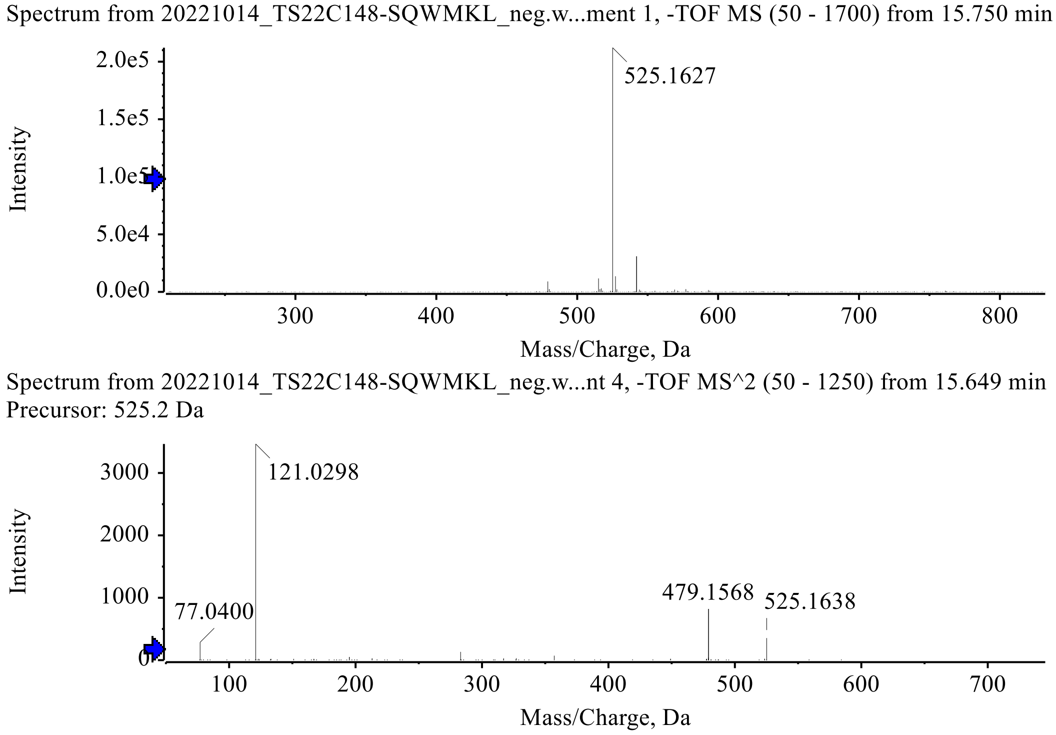

● MS^1/MS^2 spectrum of No.19, negative

Spectrum from 20221014_TS22C148-SQWMKL_neg.w...ment 1, -TOF MS (50 - 1700) from 16.517 min

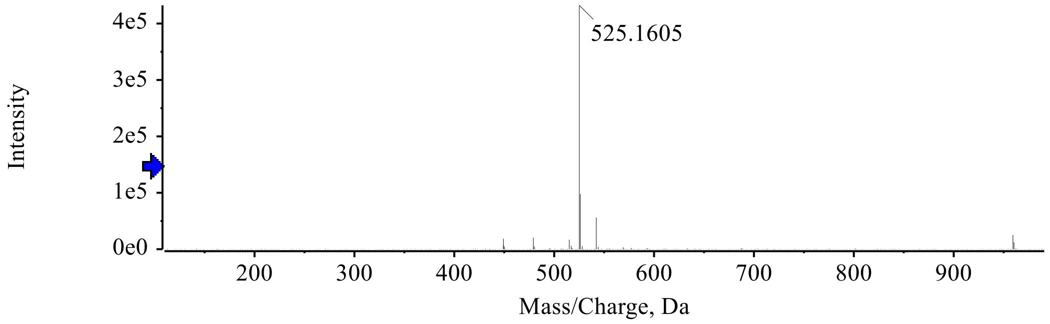

Spectrum from 20221014_TS22C148-SQWMKL_neg.w...nt 4, -TOF MS^2 (50 - 1250) from 16.428 min
Precursor: 525.2 Da

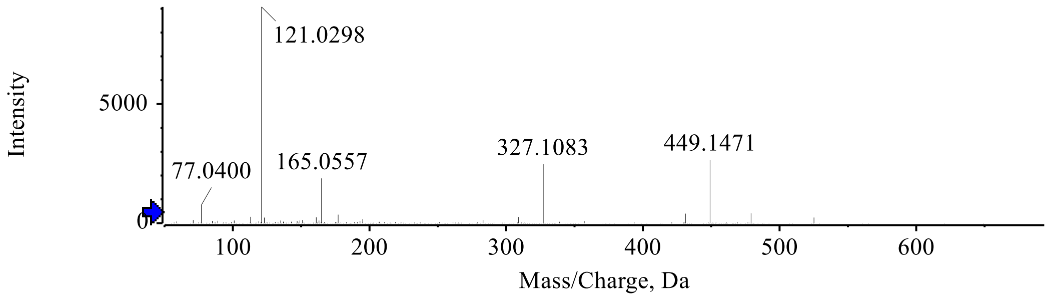

- MS^1/MS^2 spectrum of No.20, positive

Spectrum from 20221014_TS22C148-SQWMKL_pos....ment 1, +TOF MS (50 - 1700) from 16.838 min

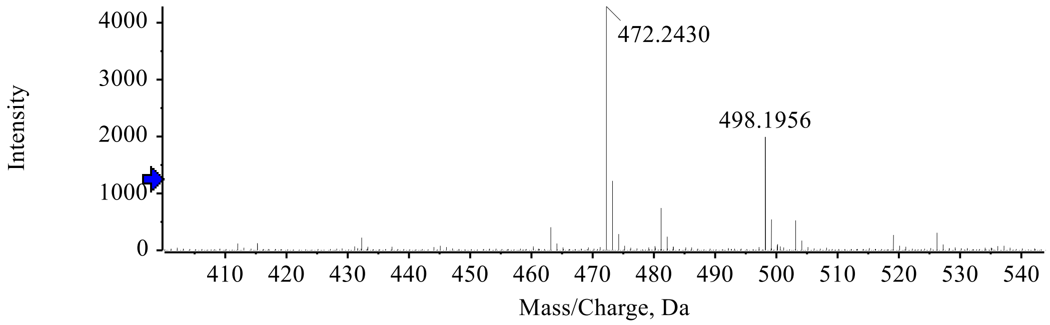

Spectrum from 20221014_TS22C148-SQWMKL_pos.w...nt 2, +TOF MS^2 (50 - 1250) from 16.783 min
Precursor: 472.2 Da, CE: 40.0

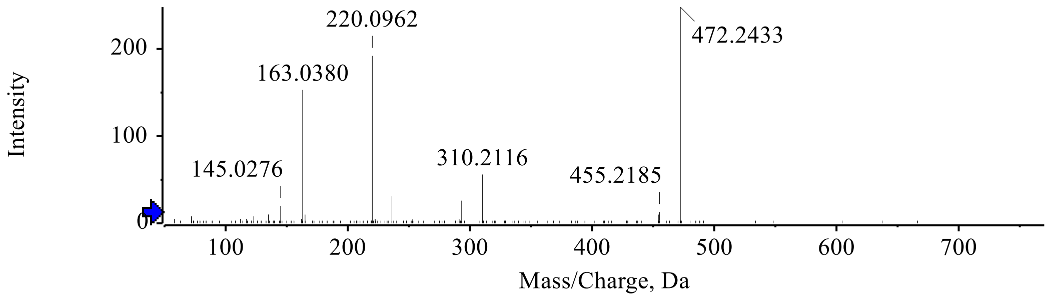

- MS^1/MS^2 spectrum of No.21, negative

Spectrum from 20221014_TS22C148-SQWMKL_neg.w...ment 1, -TOF MS (50 - 1700) from 17.578 min

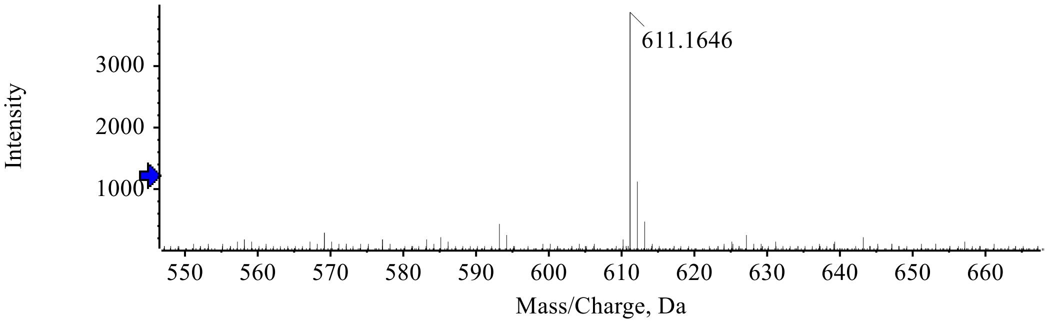

Spectrum from 20221014_TS22C148-SQWMKL_neg.w...nt 5, -TOF MS^2 (50 - 1250) from 17.477 min
Precursor: 611.2 Da

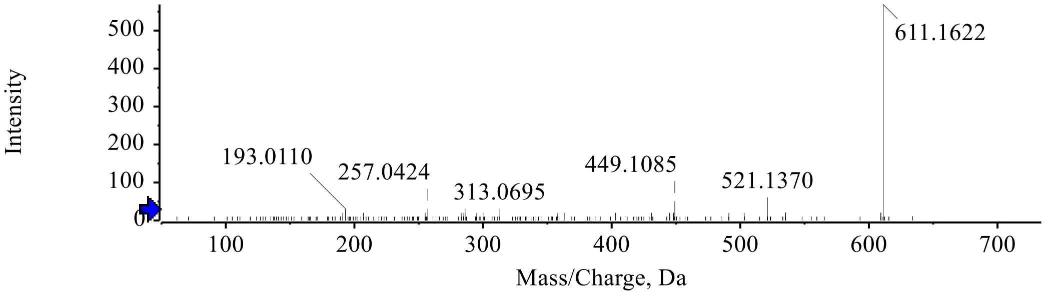

● MS^1/MS^2 spectrum of No.22, negative

Spectrum from 20221014_TS22C148-SQWMKL_neg.w...ment 1, -TOF MS (50 - 1700) from 18.760 min

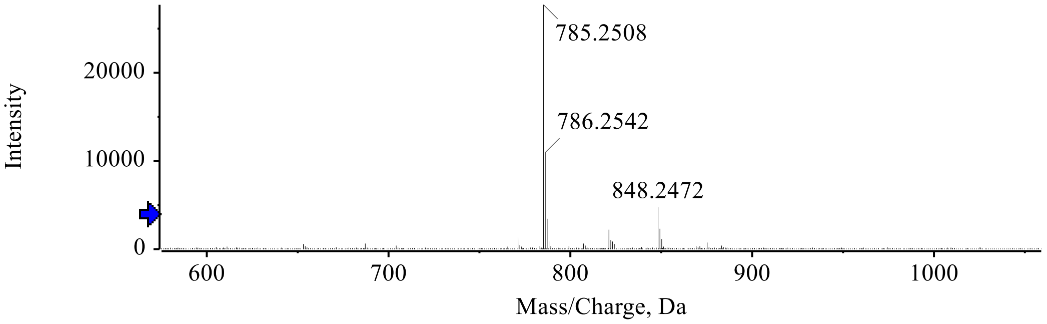

Spectrum from 20221014_TS22C148-SQWMKL_neg.w...nt 5, -TOF MS^2 (50 - 1250) from 18.706 min
Precursor: 785.3 Da

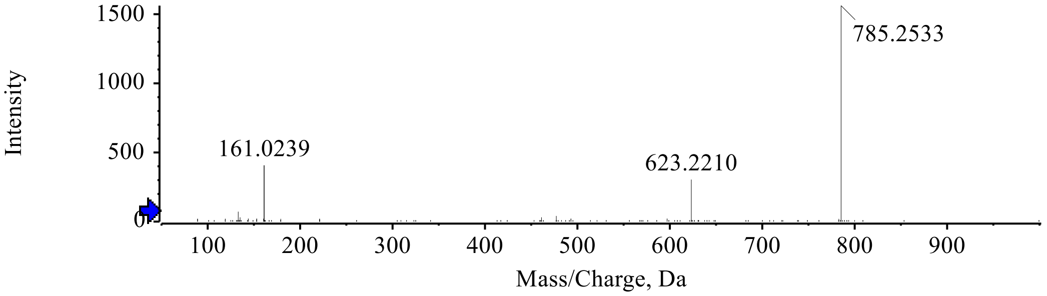

● MS^1/MS^2 spectrum of No.23, negative

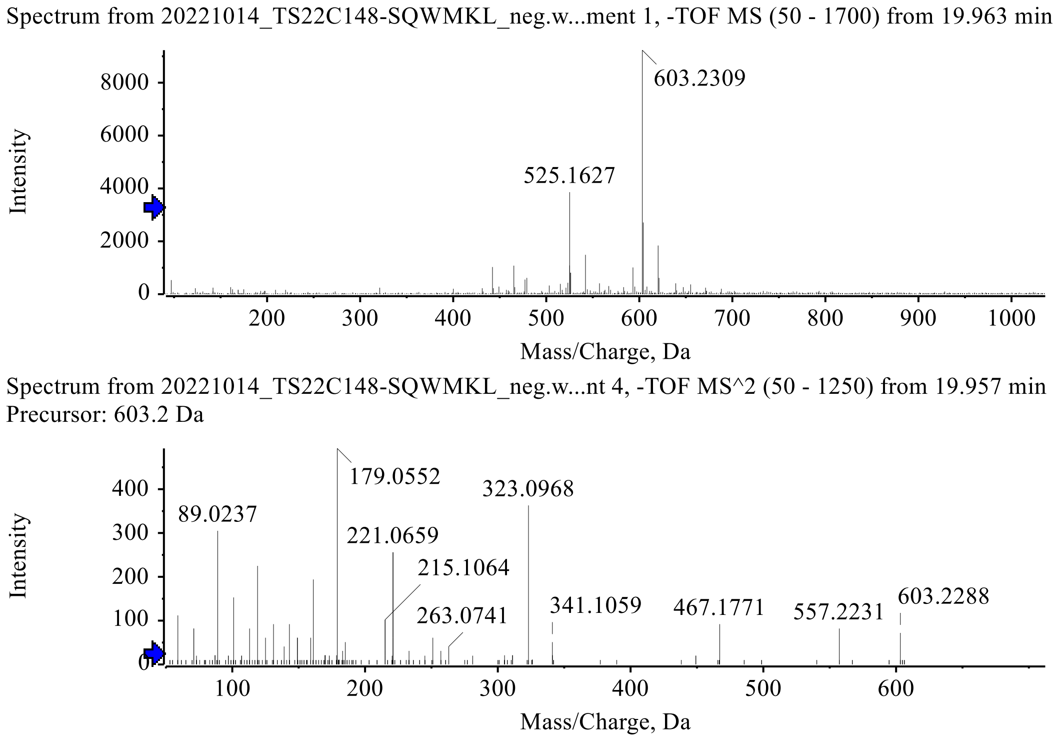

● MS¹/MS² spectrum of No.24, negative

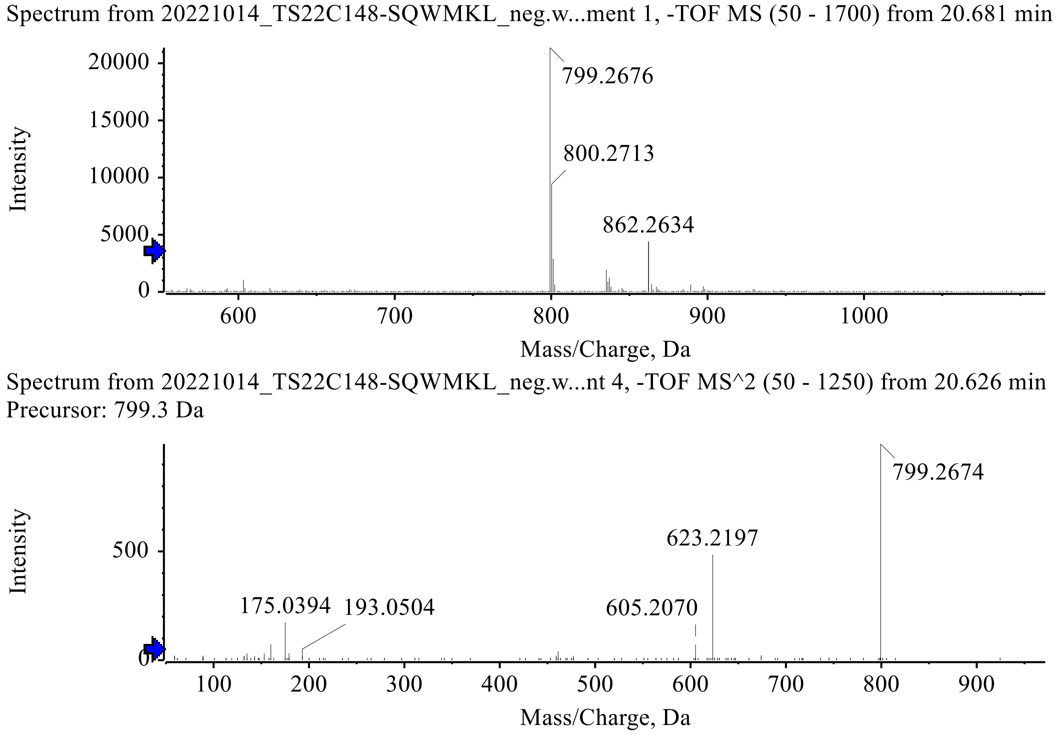

● MS¹/MS² spectrum of No.25, negative

Spectrum from 20221014_TS22C148-SQWMKL_neg.w...ment 1, -TOF MS (50 - 1700) from 20.861 min

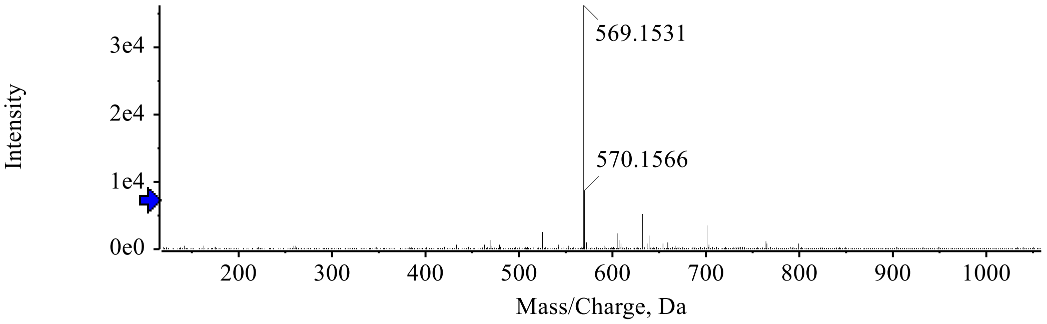

Spectrum from 20221014_TS22C148-SQWMKL_neg.w...nt 3, -TOF MS^2 (50 - 1250) from 20.817 min
Precursor: 569.2 Da

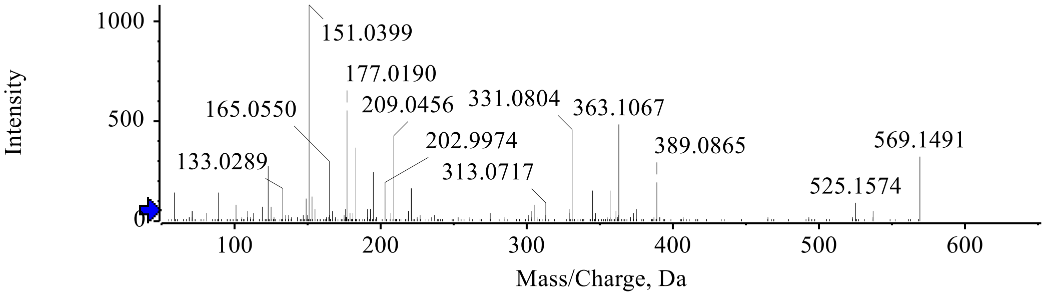

● MS¹/MS² spectrum of No.26, negative

Spectrum from 20221014_TS22C148-SQWMKL_neg.w...ment 1, -TOF MS (50 - 1700) from 20.954 min

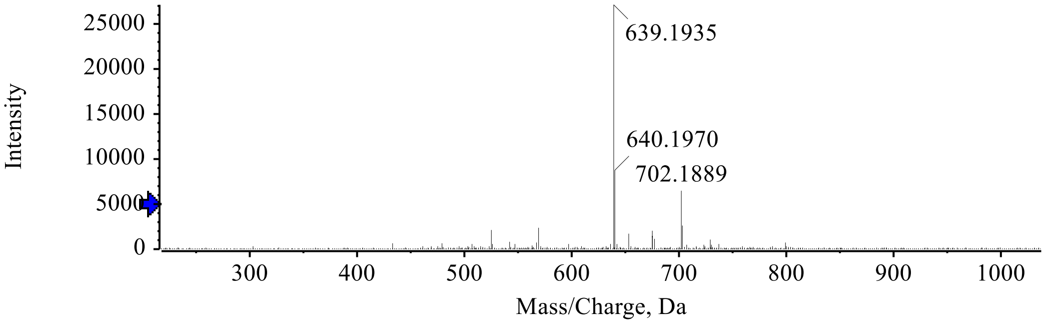

Spectrum from 20221014_TS22C148-SQWMKL_neg.w...nt 2, -TOF MS^2 (50 - 1250) from 20.933 min
Precursor: 639.2 Da

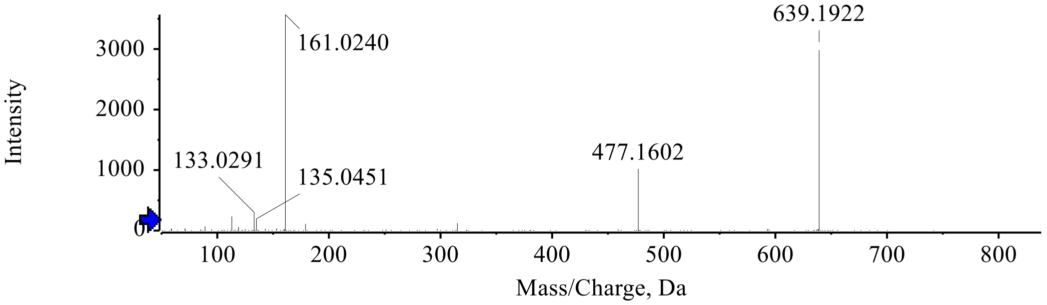

● MS¹/MS² spectrum of No.27, negative

Spectrum from 20221014_TS22C148-SQWMKL_neg.w...ment 1, -TOF MS (50 - 1700) from 21.750 min

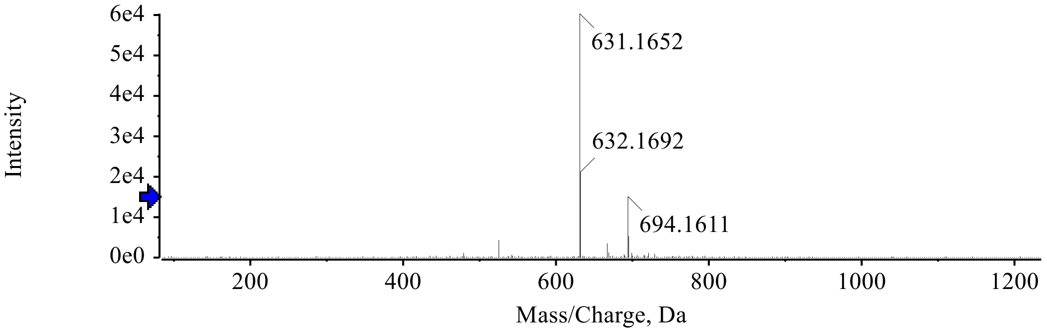

Spectrum from 20221014_TS22C148-SQWMKL_neg.w...nt 3, -TOF MS^2 (50 - 1250) from 21.754 min
Precursor: 631.2 Da

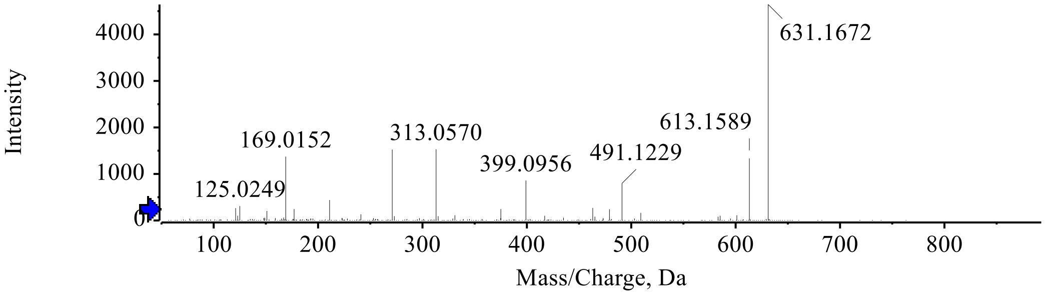

- MS^1/MS^2 spectrum of No.28, positive

Spectrum from 20221014_TS22C148-SQWMKL_pos....ment 1, +TOF MS (50 - 1700) from 22.109 min

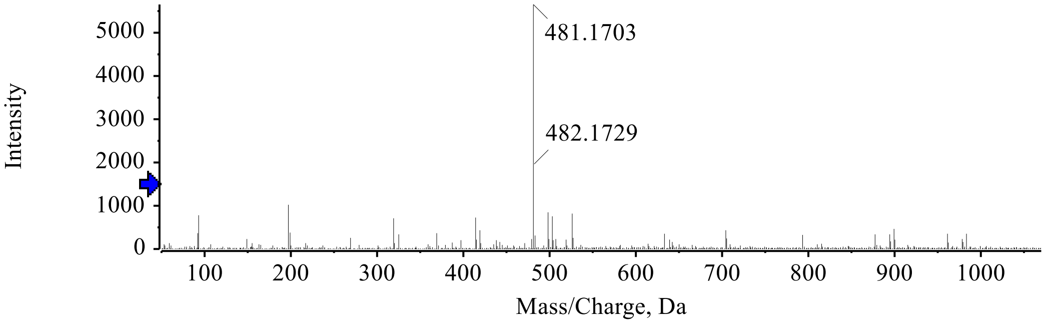

Spectrum from 20221014_TS22C148-SQWMKL_pos.w...nt 4, +TOF MS^2 (50 - 1250) from 22.054 min
Precursor: 482.2 Da, CE: 40.0

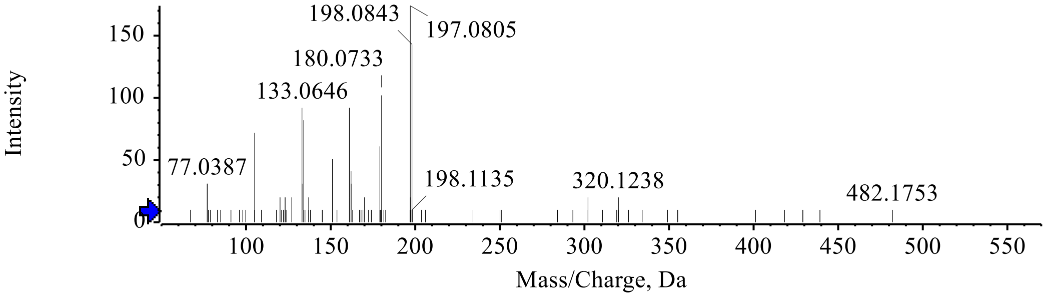

- MS^1/MS^2 spectrum of No.29, negative

Spectrum from 20221014_TS22C148-SQWMKL_neg.w...ment 1, -TOF MS (50 - 1700) from 22.143 min

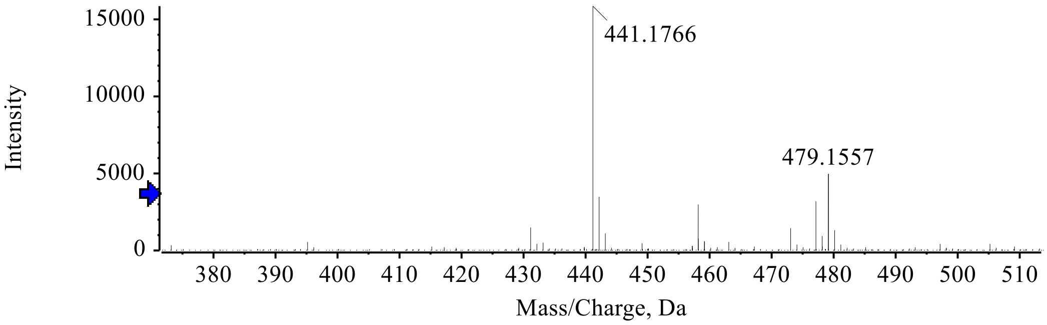

Spectrum from 20221014_TS22C148-SQWMKL_neg.w...nt 2, -TOF MS^2 (50 - 1250) from 22.111 min
Precursor: 441.2 Da

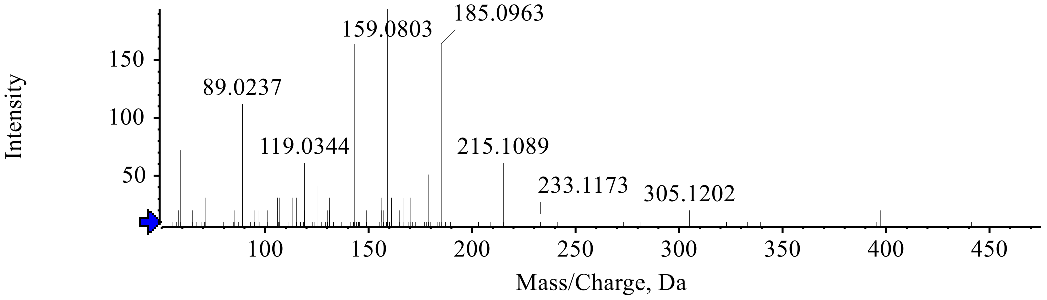

- MS[1]/MS[2] spectrum of No.30, negative

Spectrum from 20221014_TS22C148-SQWMKL_neg.w...ment 1, -TOF MS (50 - 1700) from 22.251 min

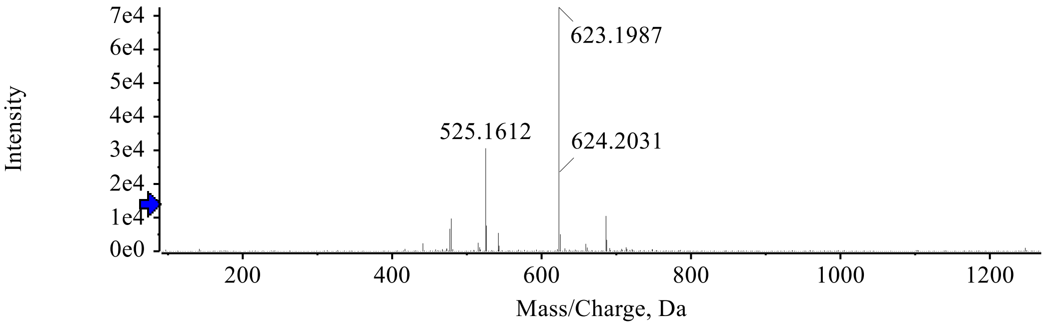

Spectrum from 20221014_TS22C148-SQWMKL_neg.w...nt 4, -TOF MS^2 (50 - 1250) from 22.185 min
Precursor: 623.2 Da

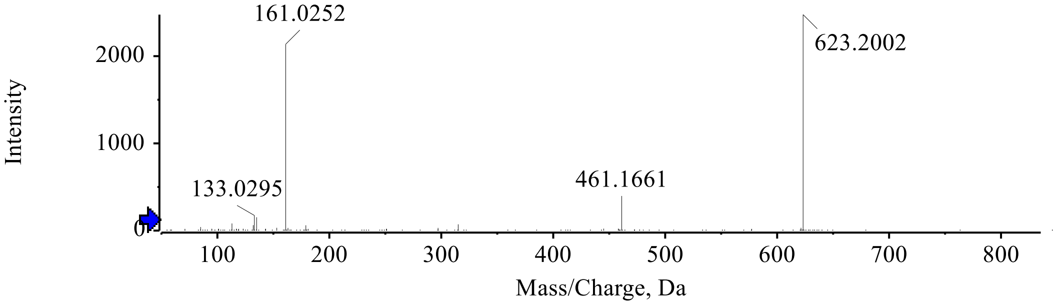

- MS[1]/MS[2] spectrum of No.31, negative

Spectrum from 20221014_TS22C148-SQWMKL_neg.w...ment 1, -TOF MS (50 - 1700) from 22.530 min

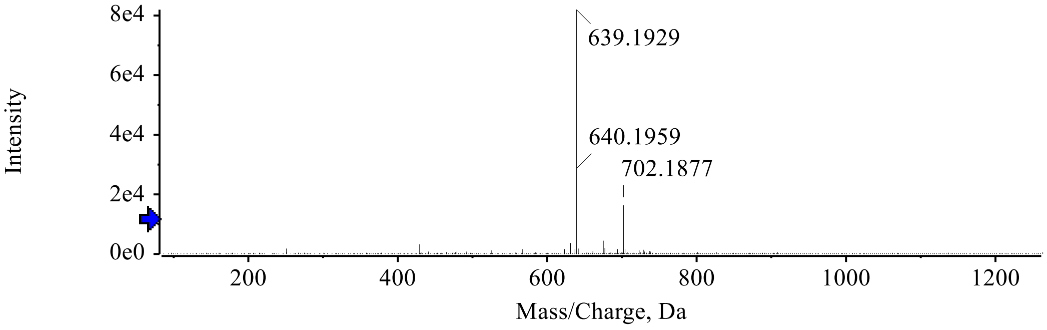

Spectrum from 20221014_TS22C148-SQWMKL_neg.w...nt 5, -TOF MS^2 (50 - 1250) from 22.478 min
Precursor: 639.2 Da

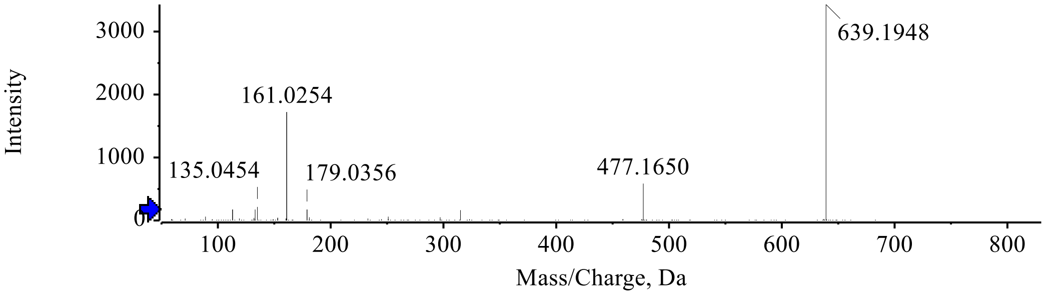

● MS^1/MS^2 spectrum of No.32, negative

Spectrum from 20221014_TS22C148-SQWMKL_neg.w...ment 1, -TOF MS (50 - 1700) from 22.821 min

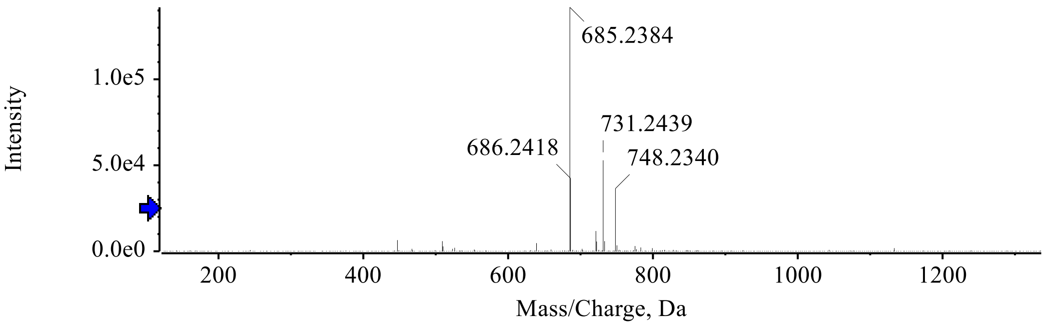

Spectrum from 20221014_TS22C148-SQWMKL_neg.w...nt 2, -TOF MS^2 (50 - 1250) from 22.752 min
Precursor: 685.2 Da

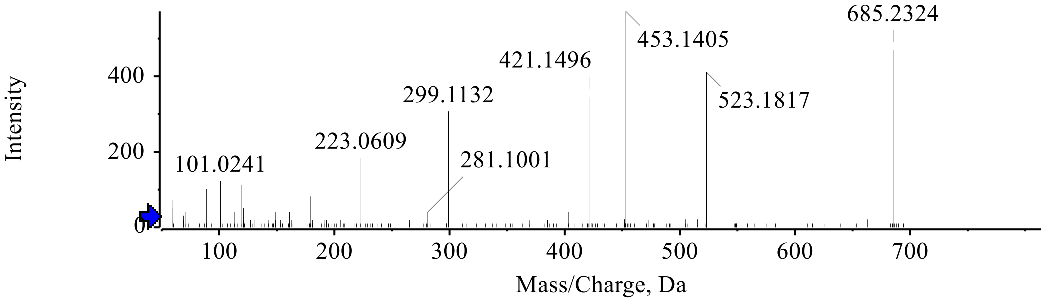

● MS^1/MS^2 spectrum of No.33, negative

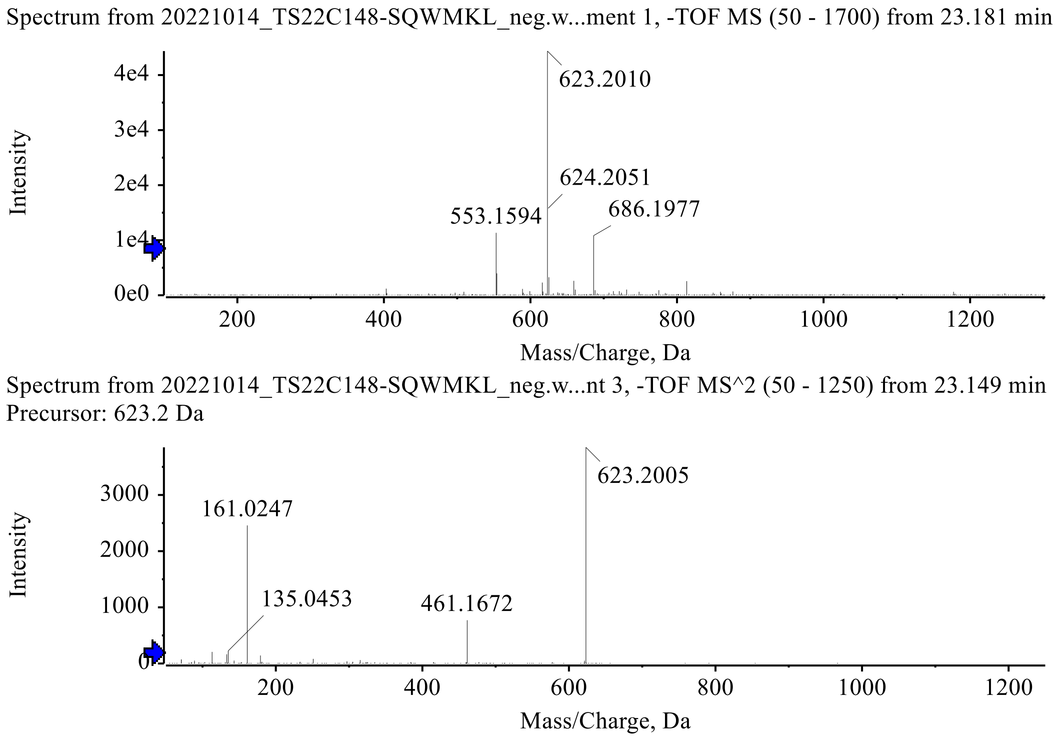

● MS[1]/MS[2] spectrum of No.34, negative

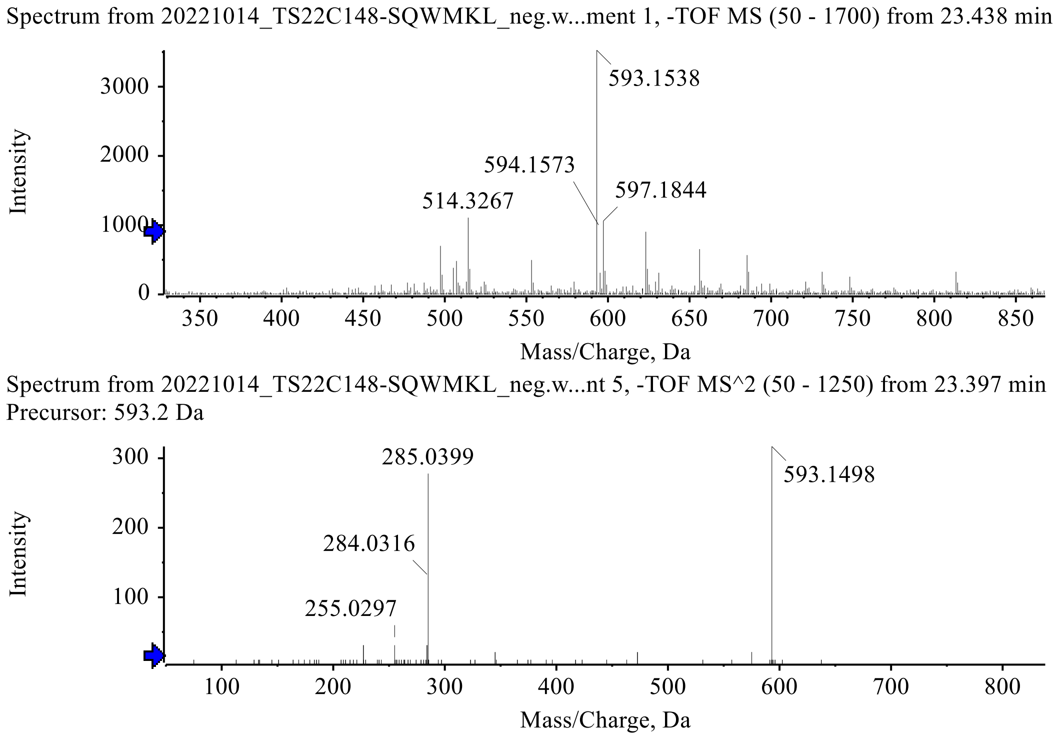

● MS[1]/MS[2] spectrum of No.35, negative

Spectrum from 20221014_TS22C148-SQWMKL_neg.w...ment 1, -TOF MS (50 - 1700) from 23.763 min

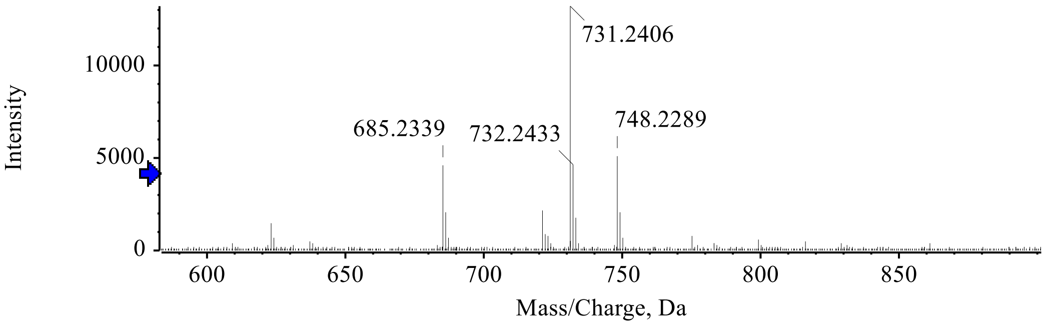

Spectrum from 20221014_TS22C148-SQWMKL_neg.w...nt 5, -TOF MS^2 (50 - 1250) from 23.746 min
Precursor: 731.2 Da

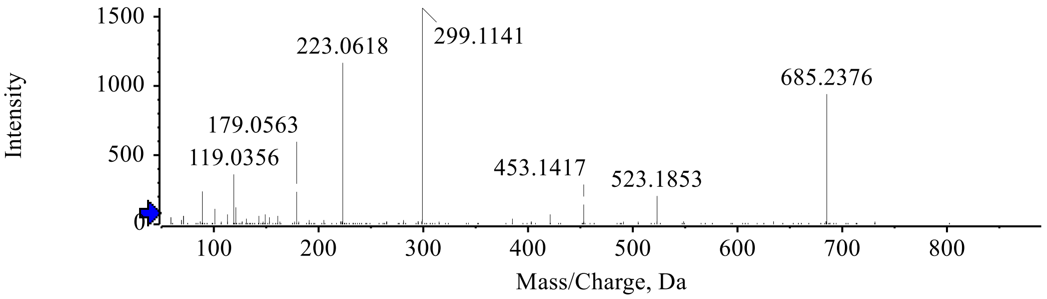

● MS¹/MS² spectrum of No.36, negative

Spectrum from 20221014_TS22C148-SQWMKL_neg.w...ment 1, -TOF MS (50 - 1700) from 24.196 min

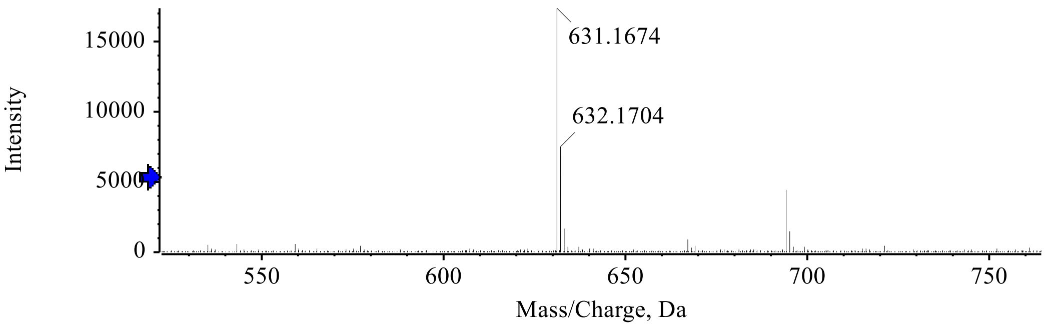

Spectrum from 20221014_TS22C148-SQWMKL_neg.w...nt 4, -TOF MS^2 (50 - 1250) from 24.153 min
Precursor: 631.2 Da

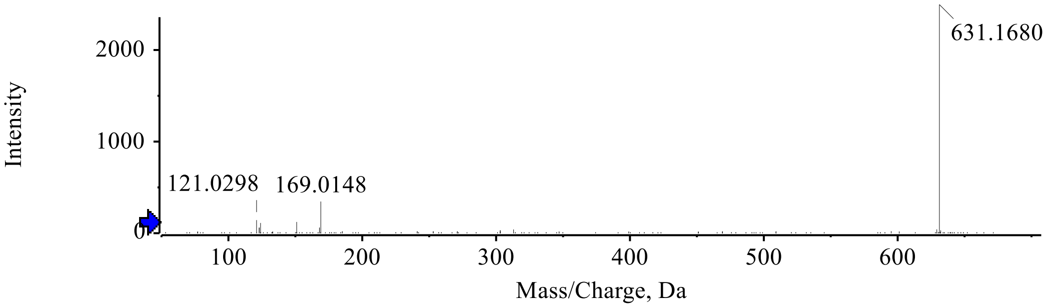

● MS¹/MS² spectrum of No.37, negative

Spectrum from 20221014_TS22C148-SQWMKL_neg.w...ment 1, -TOF MS (50 - 1700) from 24.623 min

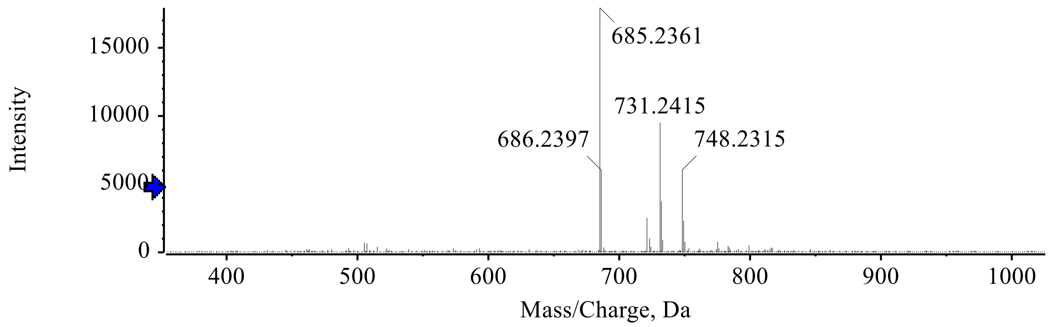

Spectrum from 20221014_TS22C148-SQWMKL_neg.w...nt 3, -TOF MS^2 (50 - 1250) from 24.603 min
Precursor: 685.2 Da

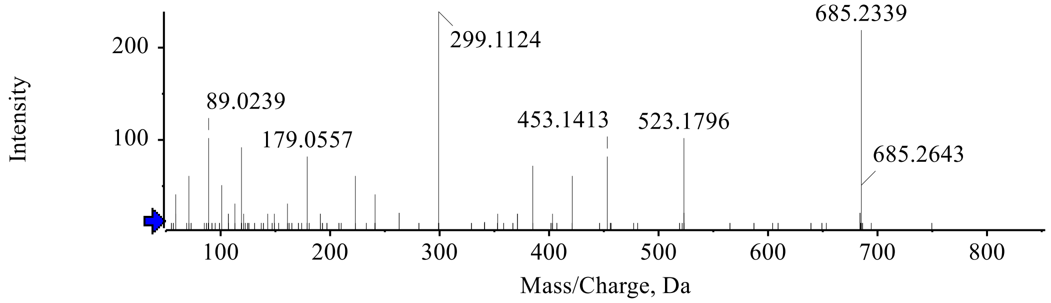

● MS^1/MS^2 spectrum of No.38, negative

Spectrum from 20221014_TS22C148-SQWMKL_neg.w...ment 1, -TOF MS (50 - 1700) from 25.415 min

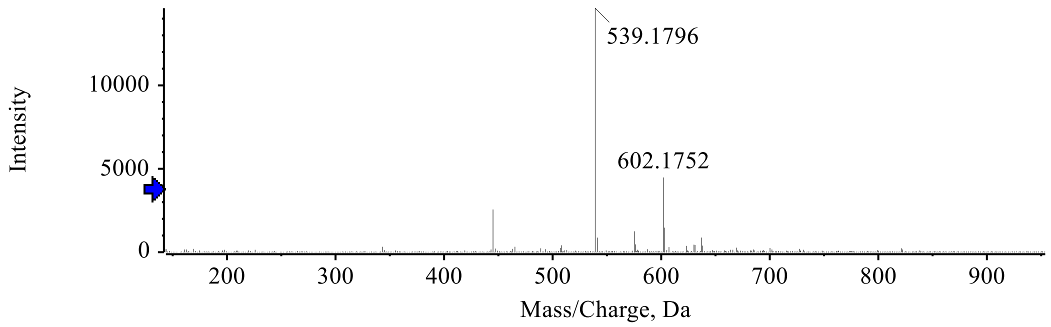

Spectrum from 20221014_TS22C148-SQWMKL_neg.w...nt 3, -TOF MS^2 (50 - 1250) from 25.347 min
Precursor: 539.2 Da

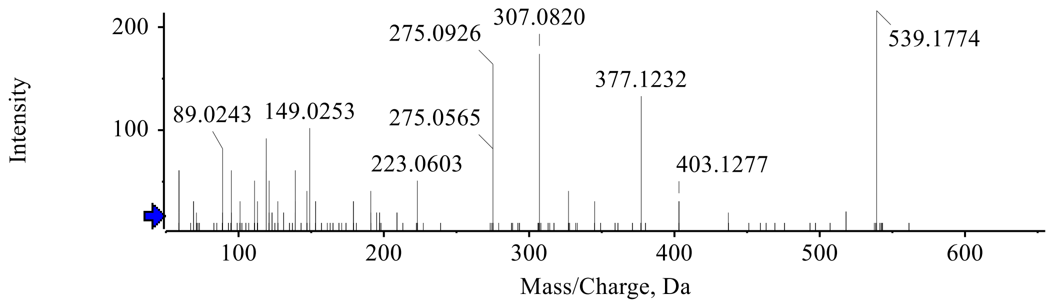

● MS^1/MS^2 spectrum of No.39, negative

Spectrum from 20221014_TS22C148-SQWMKL_neg.w...ment 1, -TOF MS (50 - 1700) from 25.681 min

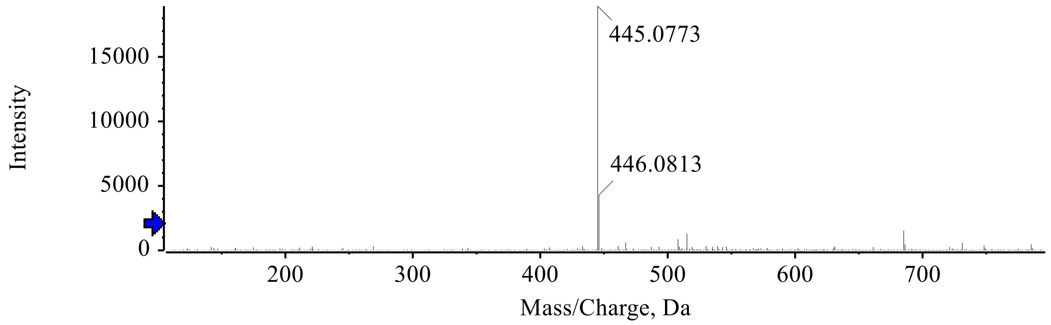

Spectrum from 20221014_TS22C148-SQWMKL_neg.w...nt 2, -TOF MS^2 (50 - 1250) from 25.660 min
Precursor: 445.1 Da

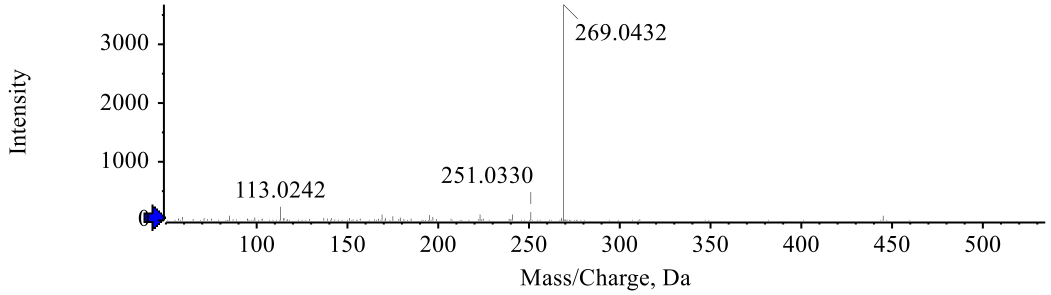

● MS^1/MS^2 spectrum of No.40, negative

Spectrum from 20221014_TS22C148-SQWMKL_neg.w...ment 1, -TOF MS (50 - 1700) from 27.742 min

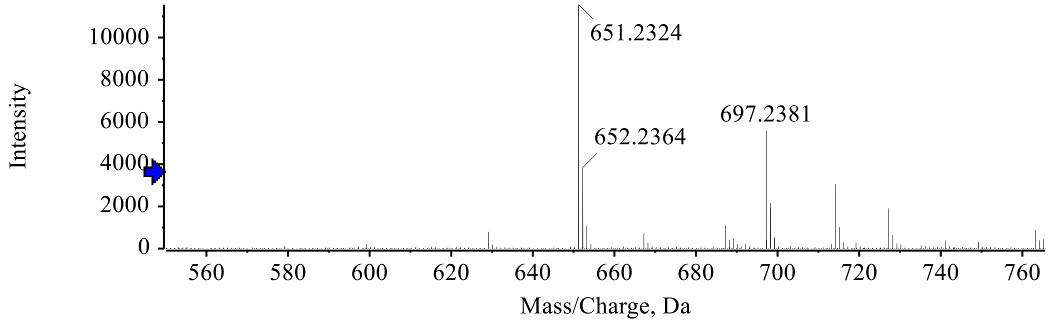

Spectrum from 20221014_TS22C148-SQWMKL_neg.w...nt 4, -TOF MS^2 (50 - 1250) from 27.699 min
Precursor: 651.2 Da

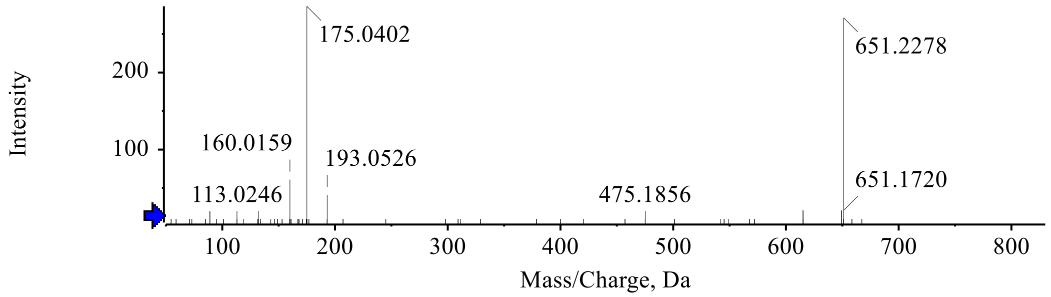

● MS^1/MS^2 spectrum of No.41, negative

Spectrum from 20221014_TS22C148-SQWMKL_neg.w...ment 1, -TOF MS (50 - 1700) from 28.100 min

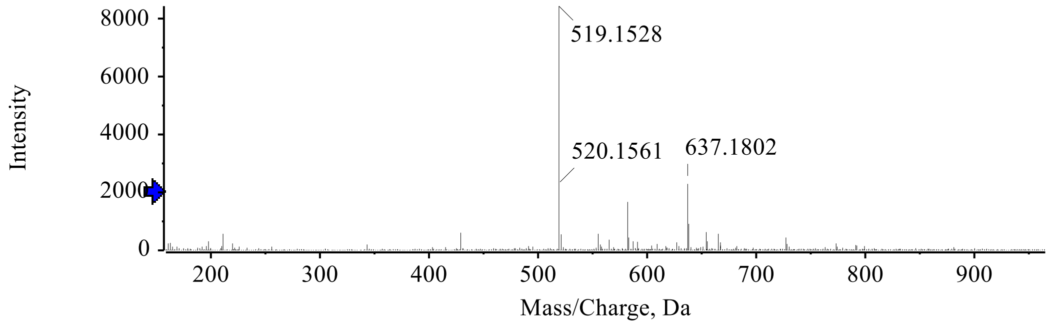

Spectrum from 20221014_TS22C148-SQWMKL_neg.w...nt 3, -TOF MS^2 (50 - 1250) from 28.080 min
Precursor: 519.2 Da

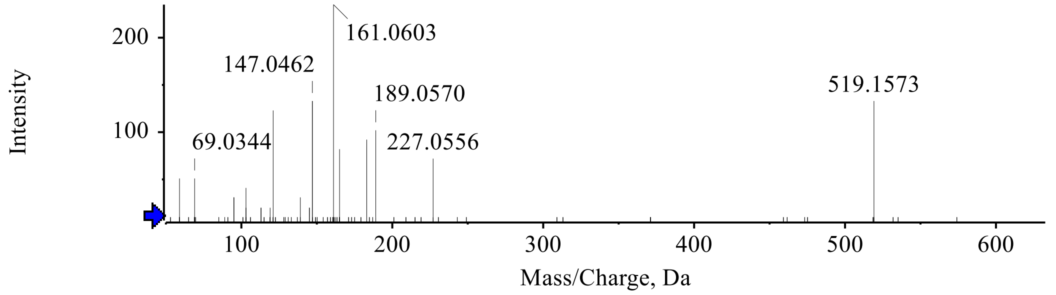

● MS[1]/MS[2] spectrum of No.42, negative

Spectrum from 20221014_TS22C148-SQWMKL_neg.w...ment 1, -TOF MS (50 - 1700) from 28.892 min

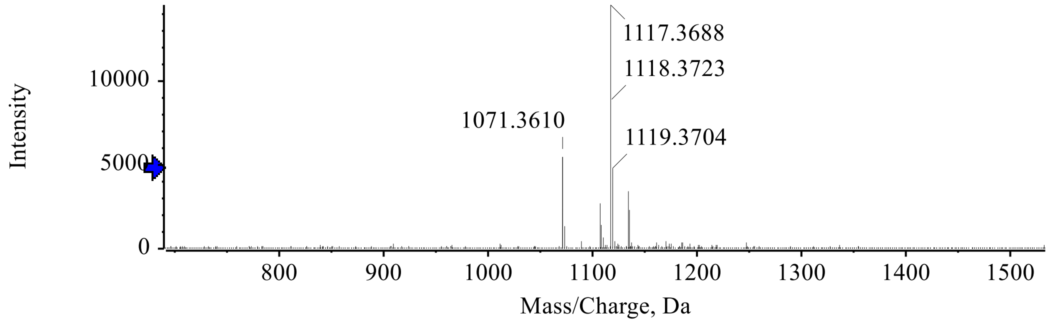

Spectrum from 20221014_TS22C148-SQWMKL_neg.w...nt 4, -TOF MS^2 (50 - 1250) from 28.897 min
Precursor: 1117.4 Da

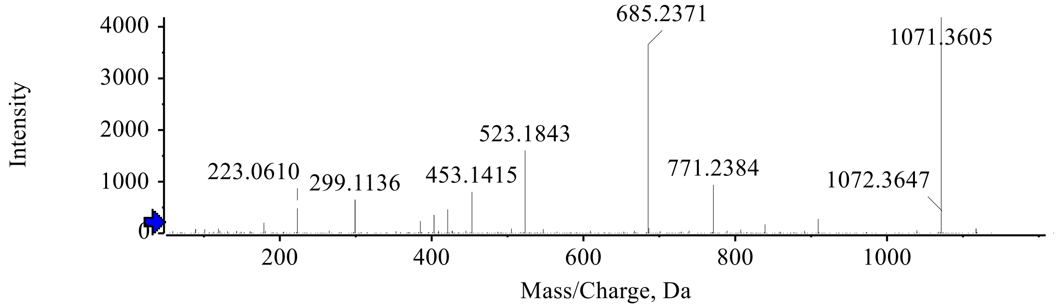

● MS[1]/MS[2] spectrum of No.43, negative

Spectrum from 20221014_TS22C148-SQWMKL_neg.w...ment 1, -TOF MS (50 - 1700) from 30.797 min

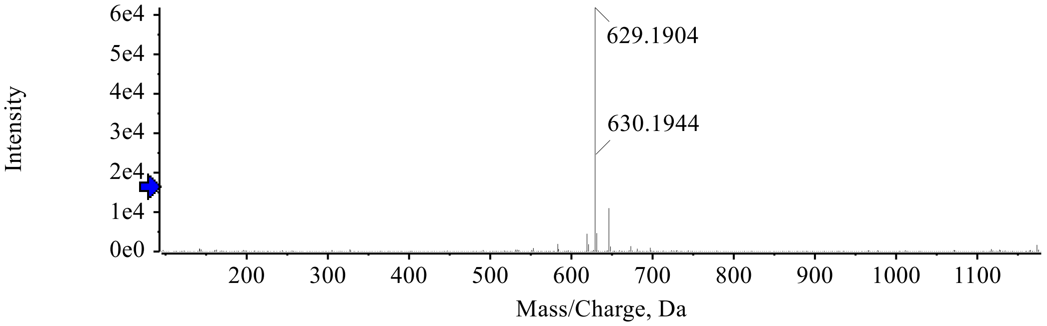

Spectrum from 20221014_TS22C148-SQWMKL_neg.w...nt 4, -TOF MS^2 (50 - 1250) from 30.714 min
Precursor: 629.2 Da

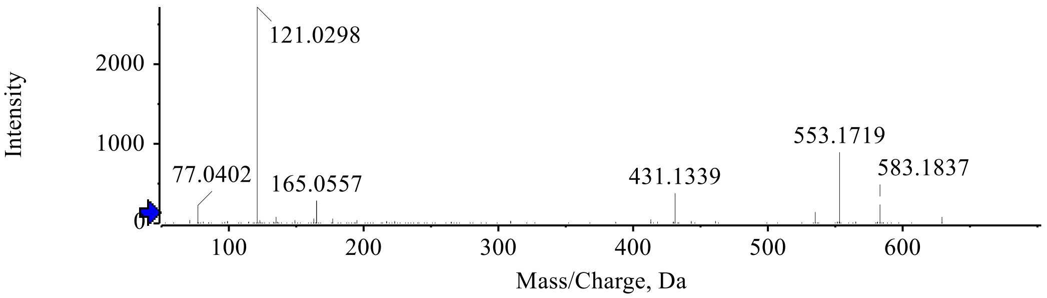

● MS[1]/MS[2] spectrum of No.44, negative

Spectrum from 20221014_TS22C148-SQWMKL_neg.w...ment 1, -TOF MS (50 - 1700) from 32.664 min

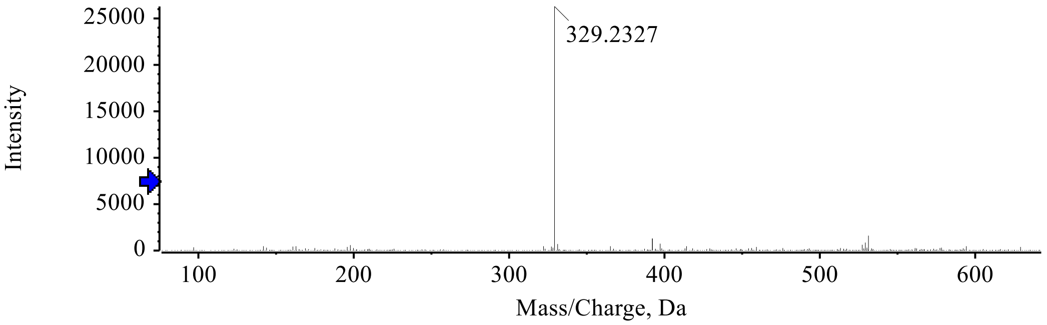

Spectrum from 20221014_TS22C148-SQWMKL_neg.w...nt 3, -TOF MS^2 (50 - 1250) from 32.596 min
Precursor: 329.2 Da

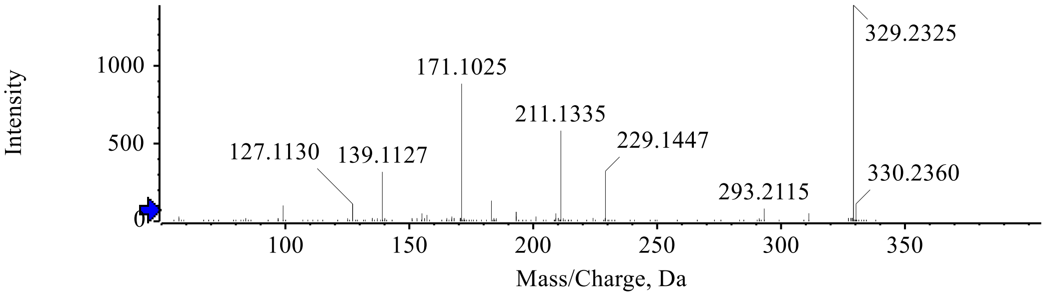

● MS[1]/MS[2] spectrum of No.45, negative

Spectrum from 20221014_TS22C148-SQWMKL_neg.w...ment 1, -TOF MS (50 - 1700) from 33.119 min

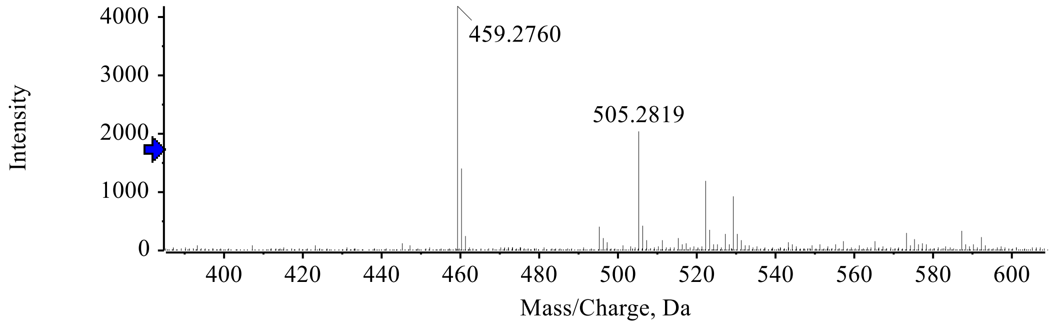

Spectrum from 20221014_TS22C148-SQWMKL_neg.w...nt 2, -TOF MS^2 (50 - 1250) from 33.097 min
Precursor: 505.3 Da

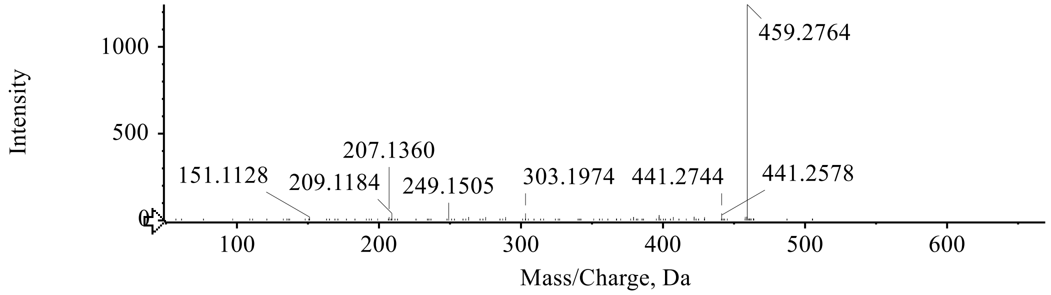

● MS[1]/MS[2] spectrum of No.46, positive

Spectrum from 20221014_TS22C148-SQWMKL_pos....ment 1, +TOF MS (50 - 1700) from 33.447 min

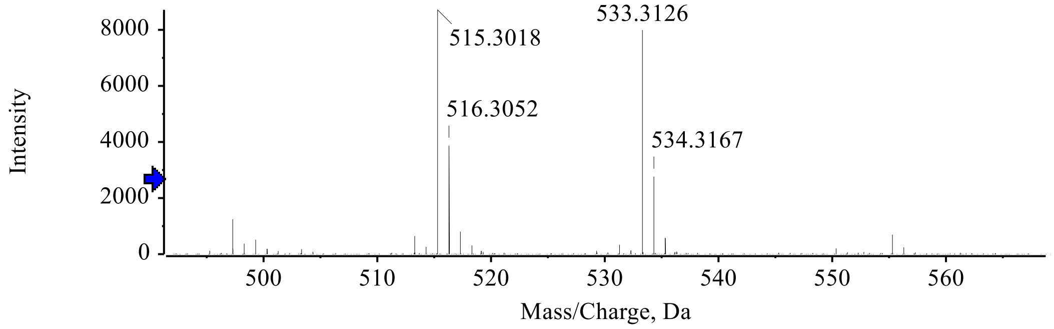

Spectrum from 20221014_TS22C148-SQWMKL_pos.w...nt 3, +TOF MS^2 (50 - 1250) from 33.427 min
Precursor: 533.3 Da, CE: 40.0

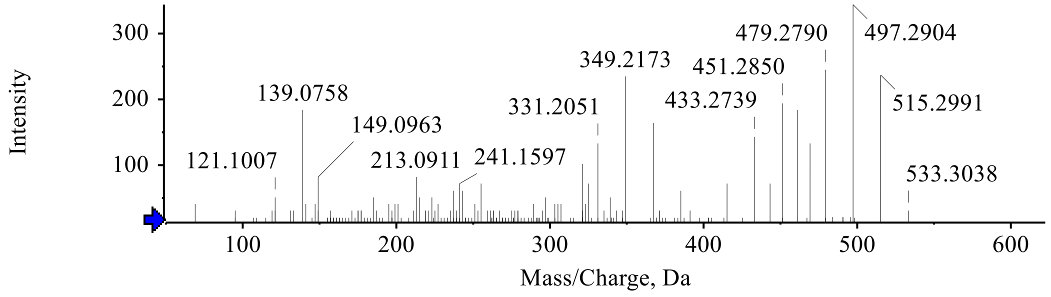

● MS[1]/MS[2] spectrum of No.47, positive

Spectrum from 20221014_TS22C148-SQWMKL_pos....ment 1, +TOF MS (50 - 1700) from 33.724 min

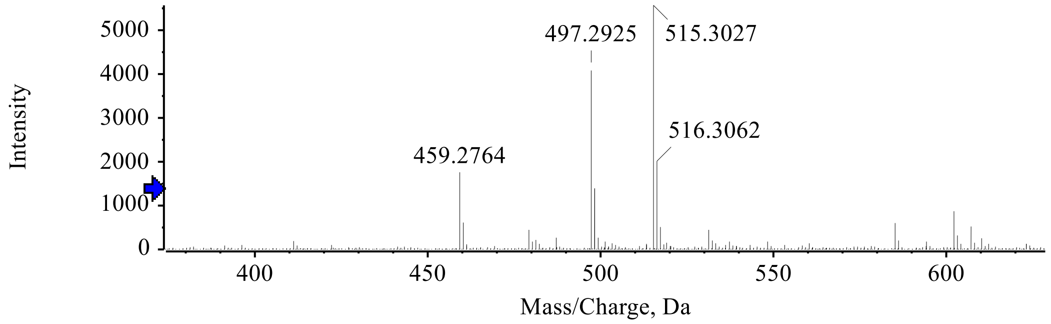

Spectrum from 20221014_TS22C148-SQWMKL_pos.w...nt 4, +TOF MS^2 (50 - 1250) from 33.657 min
Precursor: 515.3 Da, CE: 40.0

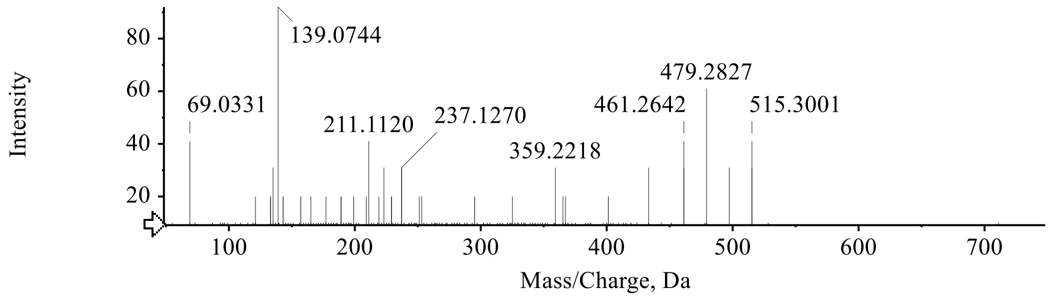

- MS[1]/MS[2] spectrum of No.48, positive

Spectrum from 20221014_TS22C148-SQWMKL_pos....ment 1, +TOF MS (50 - 1700) from 34.037 min

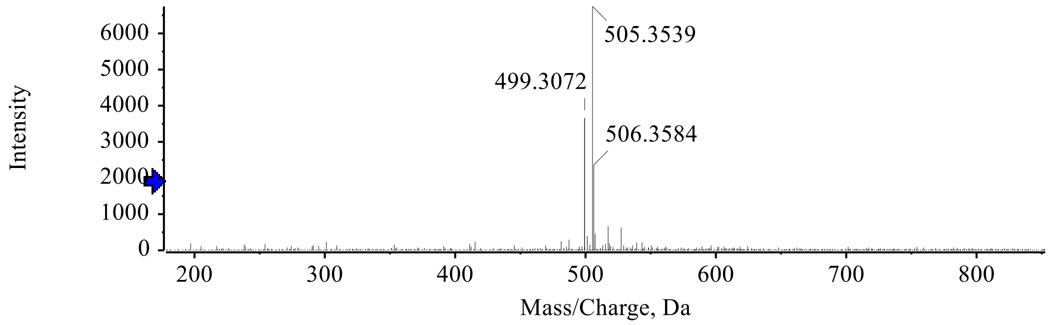

Spectrum from 20221014_TS22C148-SQWMKL_pos.w...nt 4, +TOF MS^2 (50 - 1250) from 34.013 min
Precursor: 505.4 Da, CE: 40.0

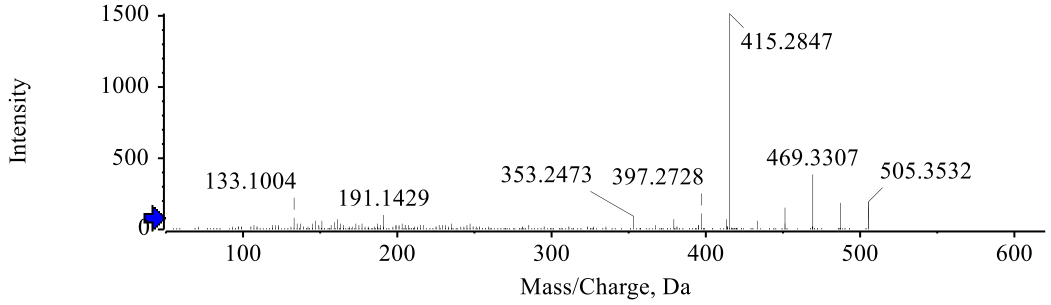

- MS[1]/MS[2] spectrum of No.49, positive

Spectrum from 20221014_TS22C148-SQWMKL_pos....ment 1, +TOF MS (50 - 1700) from 34.100 min

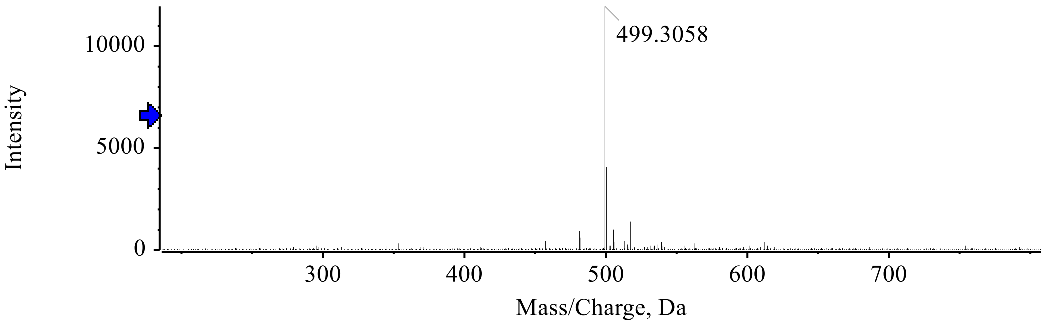

Spectrum from 20221014_TS22C148-SQWMKL_pos.w...nt 2, +TOF MS^2 (50 - 1250) from 34.022 min
Precursor: 499.3 Da, CE: 40.0

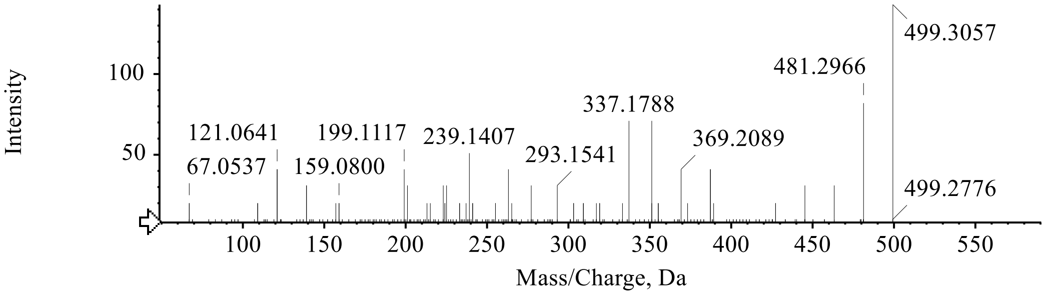

● MS[1]/MS[2] spectrum of No.50, negative

Spectrum from 20221014_TS22C148-SQWMKL_neg.w...ment 1, -TOF MS (50 - 1700) from 34.939 min

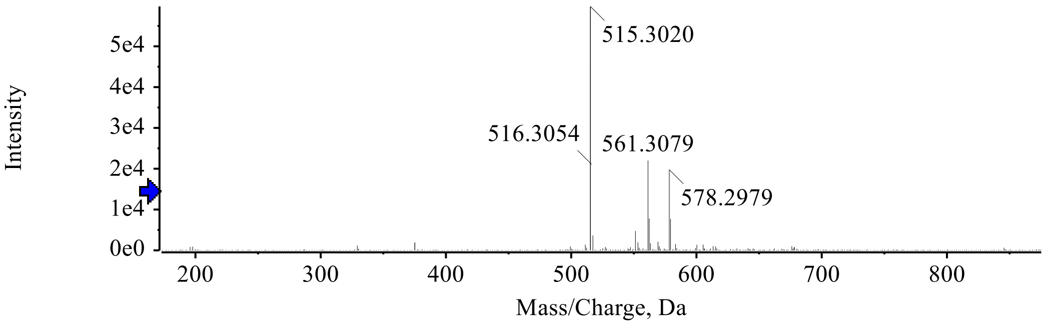

Spectrum from 20221014_TS22C148-SQWMKL_neg.w...nt 3, -TOF MS^2 (50 - 1250) from 34.902 min
Precursor: 515.3 Da

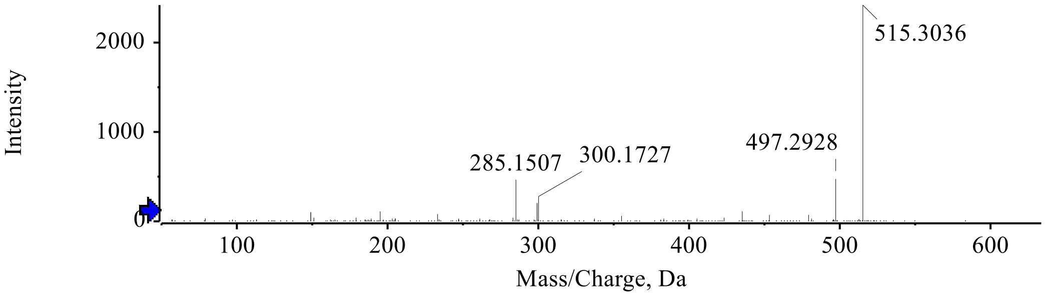

● MS[1]/MS[2] spectrum of No.51, negative

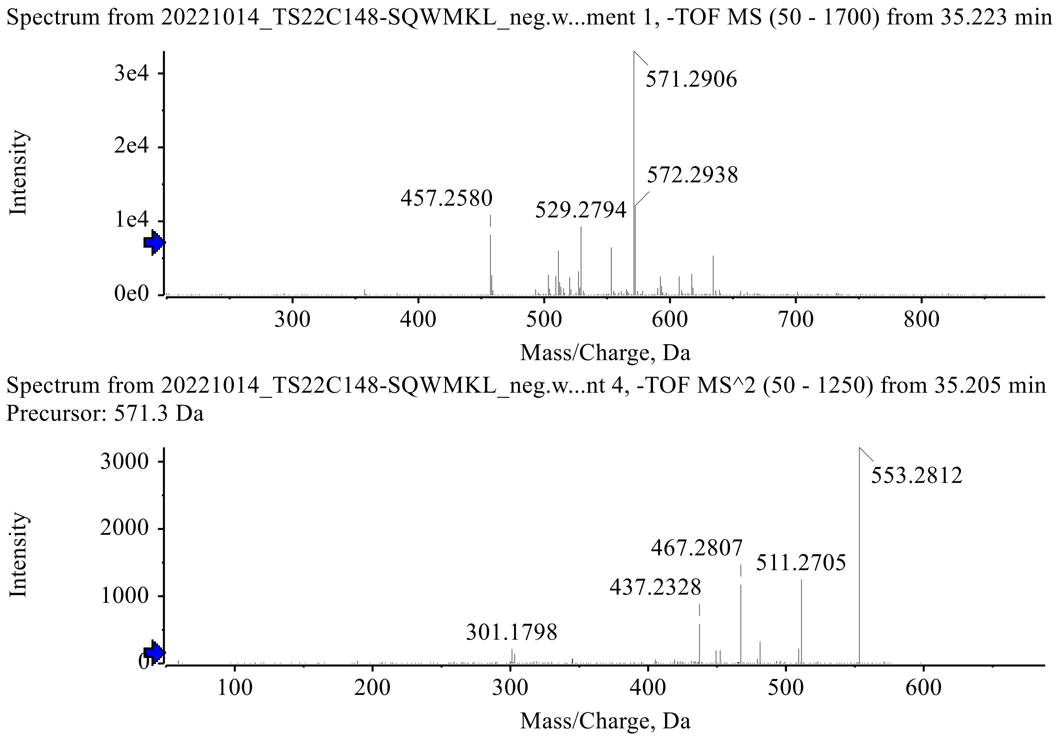

- MS^1/MS^2 spectrum of No.52, negative

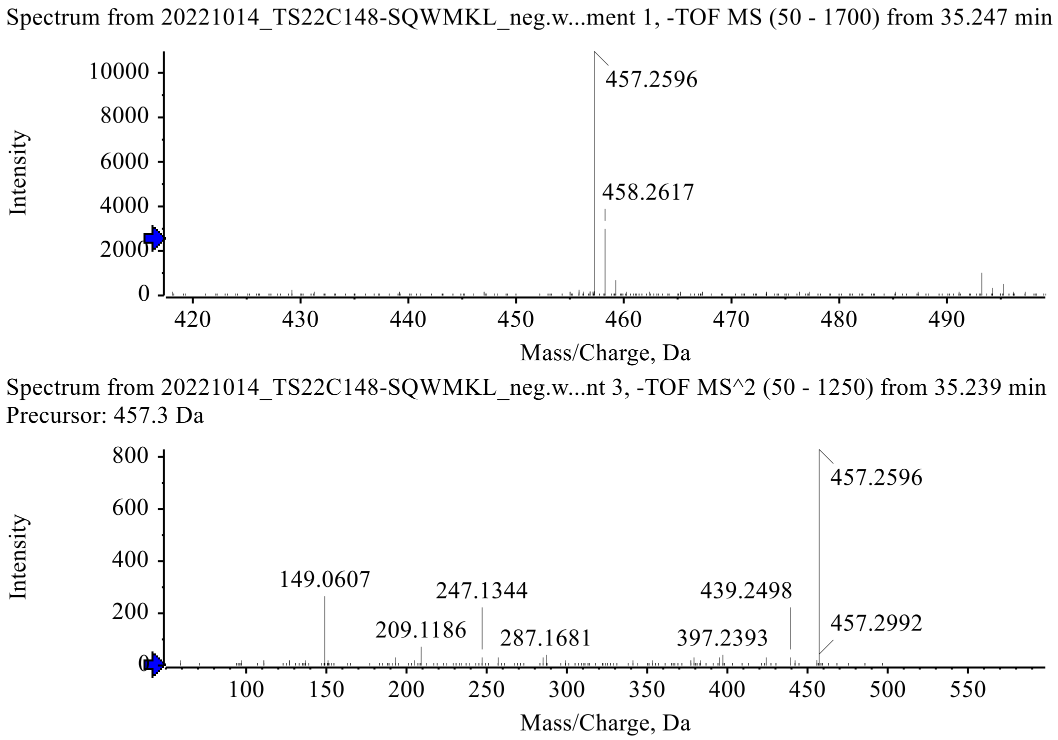

- MS^1/MS^2 spectrum of No.53, negative

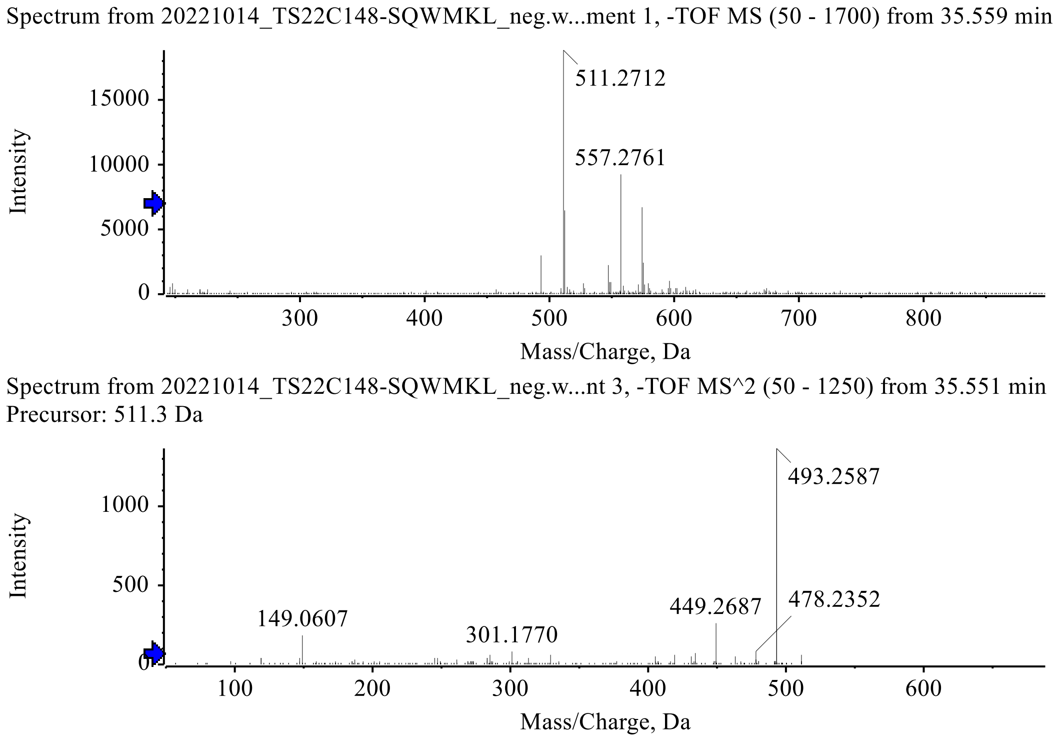

● MS[1]/MS[2] spectrum of No.54, negative

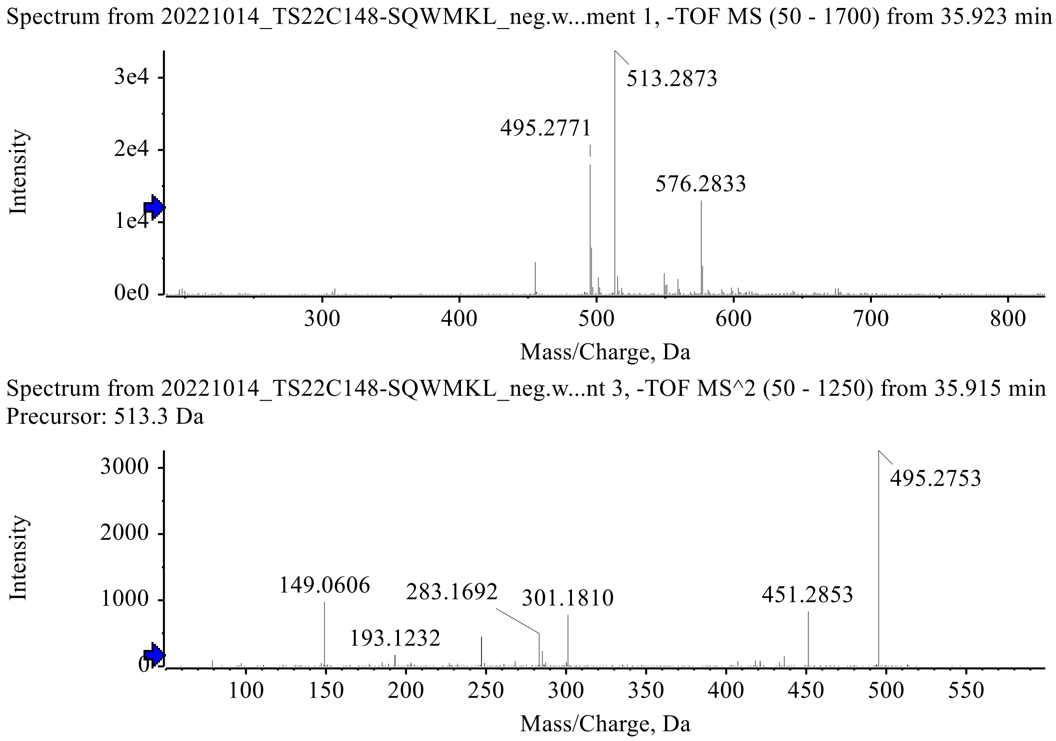

● MS[1]/MS[2] spectrum of No.55, negative

Spectrum from 20221014_TS22C148-SQWMKL_neg.w...ment 1, -TOF MS (50 - 1700) from 36.423 min

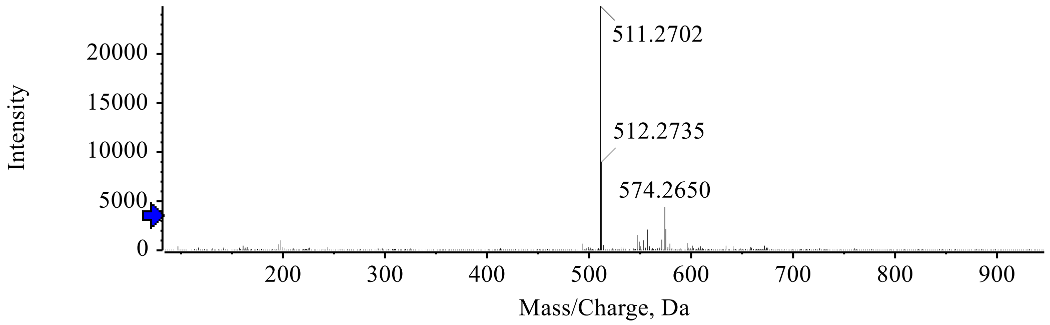

Spectrum from 20221014_TS22C148-SQWMKL_neg.w...nt 3, -TOF MS^2 (50 - 1250) from 36.368 min
Precursor: 511.3 Da

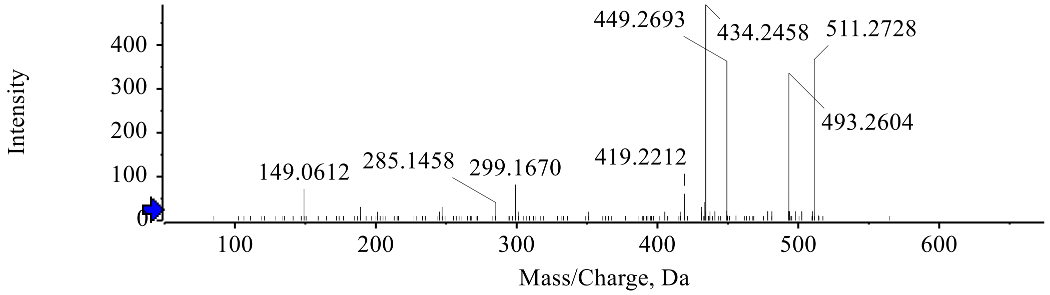

- MS[1]/MS[2] spectrum of No.56, positive

Spectrum from 20221014_TS22C148-SQWMKL_pos....ment 1, +TOF MS (50 - 1700) from 36.492 min

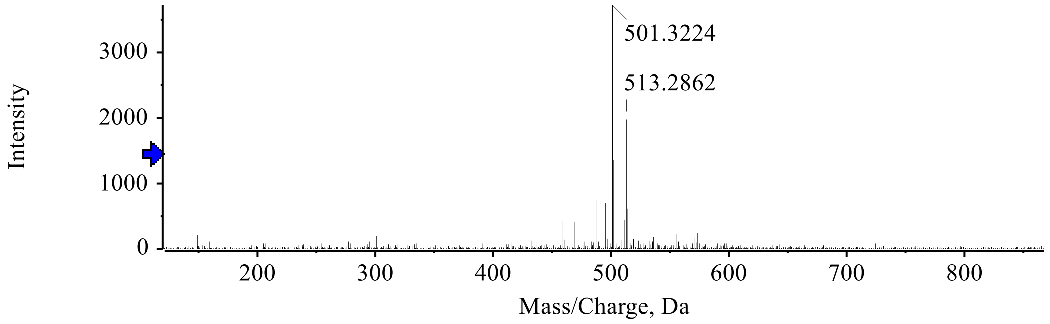

Spectrum from 20221014_TS22C148-SQWMKL_pos.w...nt 4, +TOF MS^2 (50 - 1250) from 36.497 min
Precursor: 501.3 Da, CE: 40.0

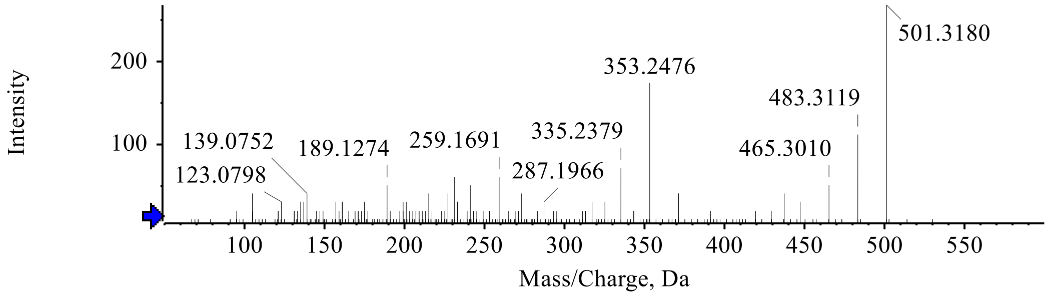

- MS[1]/MS[2] spectrum of No.57, negative

Spectrum from 20221014_TS22C148-SQWMKL_neg.w...ment 1, -TOF MS (50 - 1700) from 36.970 min

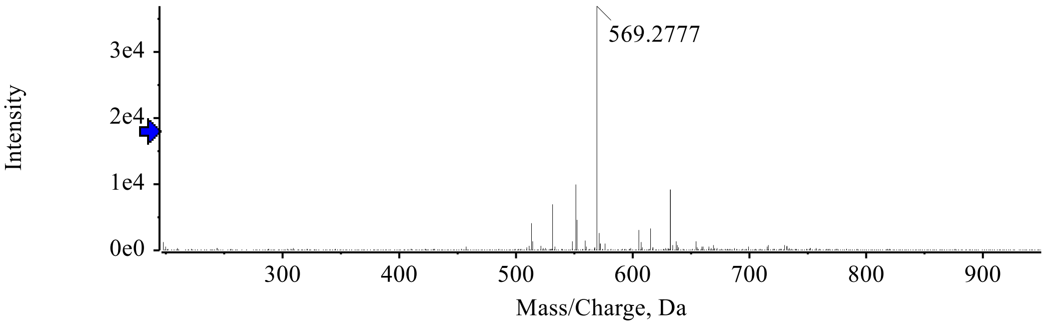

Spectrum from 20221014_TS22C148-SQWMKL_neg.w...nt 4, -TOF MS^2 (50 - 1250) from 36.963 min
Precursor: 569.3 Da

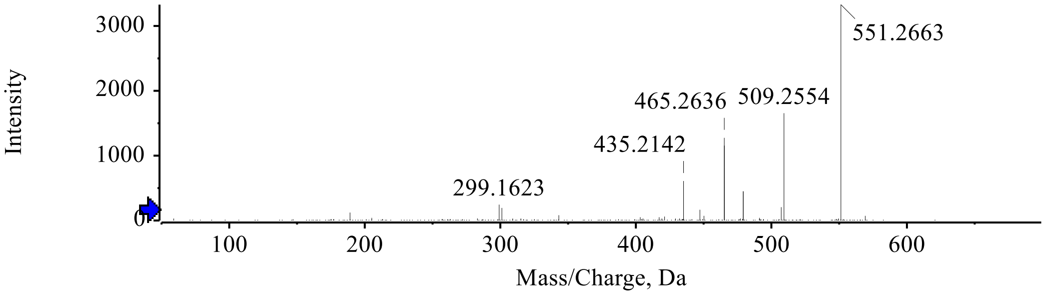

- MS¹/MS² spectrum of No.58, positive

Spectrum from 20221014_TS22C148-SQWMKL_pos....ment 1, +TOF MS (50 - 1700) from 36.977 min

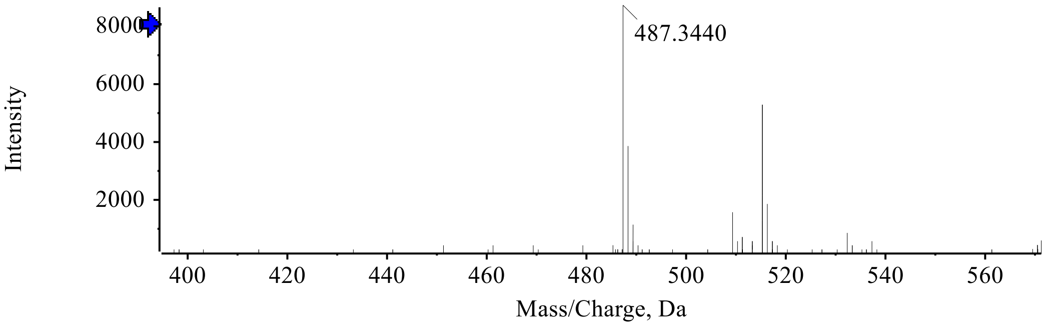

Spectrum from 20221014_TS22C148-SQWMKL_pos.w...nt 2, +TOF MS^2 (50 - 1250) from 36.920 min
Precursor: 487.3 Da, CE: 40.0

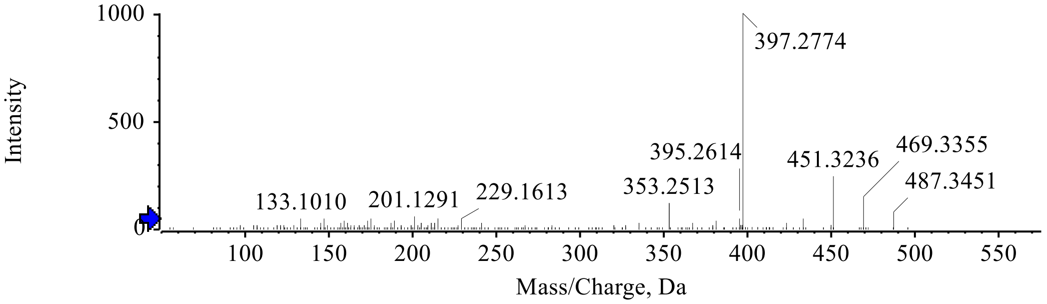

- MS¹/MS² spectrum of No.59, positive

Spectrum from 20221014_TS22C148-SQWMKL_pos....ment 1, +TOF MS (50 - 1700) from 38.642 min

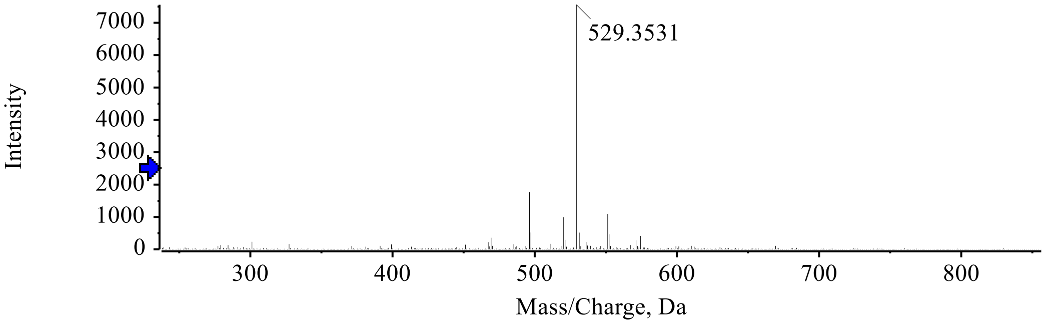

Spectrum from 20221014_TS22C148-SQWMKL_pos.w...nt 3, +TOF MS^2 (50 - 1250) from 38.634 min
Precursor: 529.4 Da, CE: 40.0

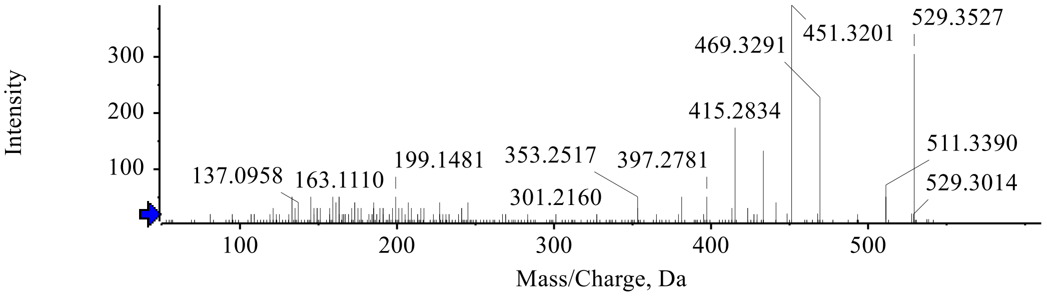

- MS[1]/MS[2] spectrum of No.60, negative

Spectrum from 20221014_TS22C148-SQWMKL_neg.w...ment 1, -TOF MS (50 - 1700) from 38.835 min

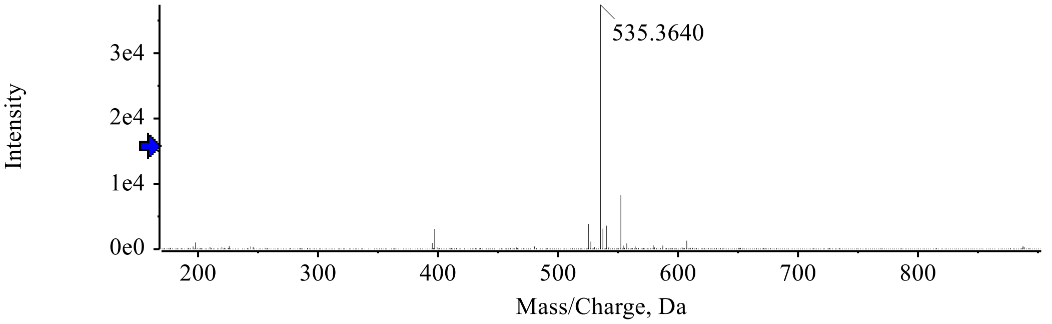

Spectrum from 20221014_TS22C148-SQWMKL_neg.w...nt 4, -TOF MS^2 (50 - 1250) from 38.793 min
Precursor: 535.4 Da

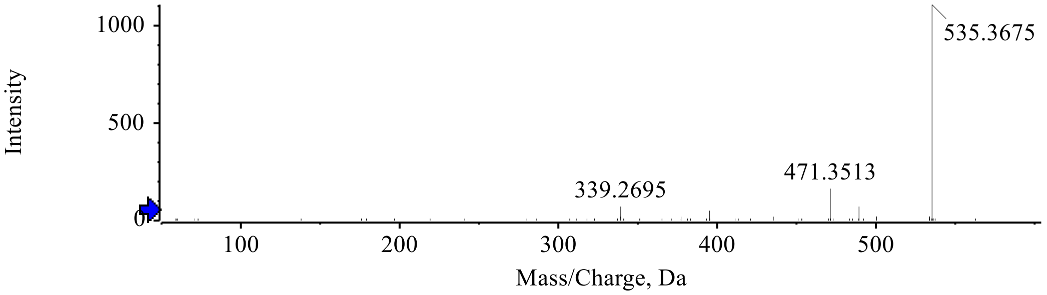

- MS[1]/MS[2] spectrum of No.61, positive

Spectrum from 20221014_TS22C148-SQWMKL_pos....ment 1, +TOF MS (50 - 1700) from 39.259 min

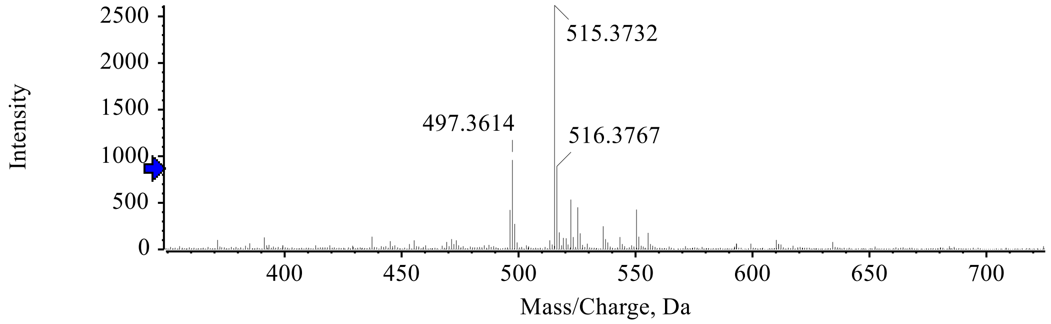

Spectrum from 20221014_TS22C148-SQWMKL_pos.w...nt 3, +TOF MS^2 (50 - 1250) from 39.238 min
Precursor: 515.4 Da, CE: 40.0

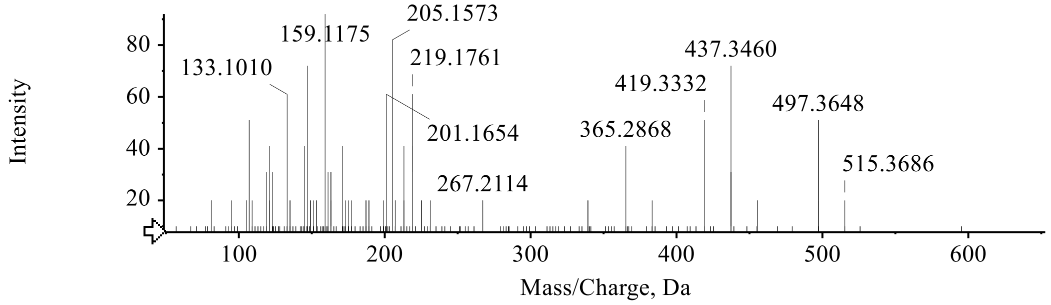

- MS[1]/MS[2] spectrum of No.62, positive

Spectrum from 20221014_TS22C148-SQWMKL_pos....ment 1, +TOF MS (50 - 1700) from 39.995 min

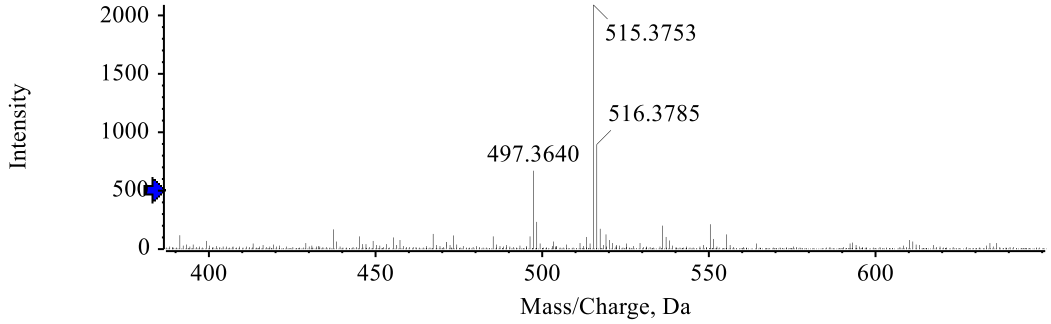

Spectrum from 20221014_TS22C148-SQWMKL_pos.w...nt 2, +TOF MS^2 (50 - 1250) from 39.974 min
Precursor: 515.4 Da, CE: 40.0

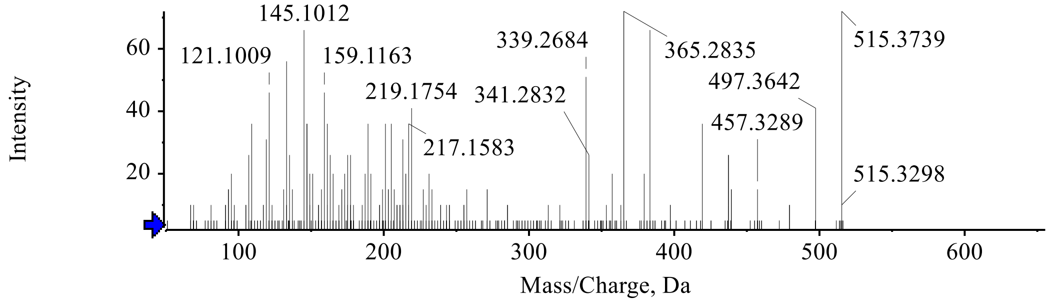

- MS[1]/MS[2] spectrum of No.63, positive

Spectrum from 20221014_TS22C148-SQWMKL_pos....ment 1, +TOF MS (50 - 1700) from 41.964 min

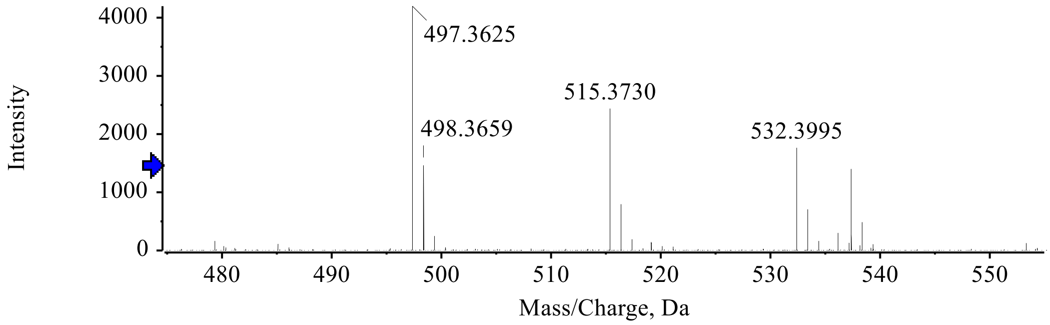

Spectrum from 20221014_TS22C148-SQWMKL_pos.w...nt 3, +TOF MS^2 (50 - 1250) from 41.968 min
Precursor: 515.4 Da, CE: 40.0

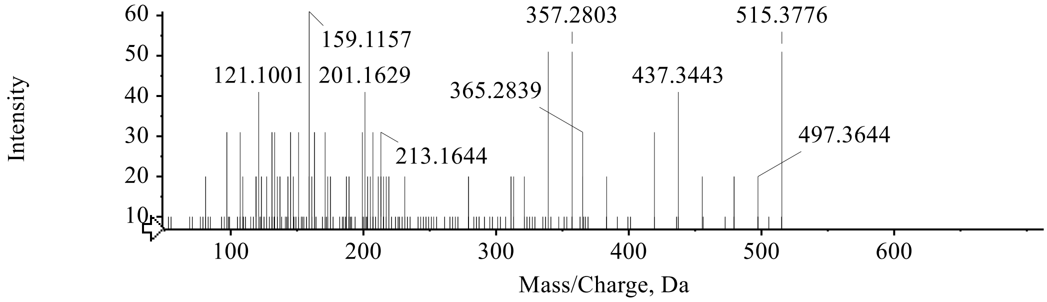

Supplement: Supplementary file 3 — Supplementary Figure 2. [file 41598_2023_32472_MOESM3_ESM.pdf]
