# Supplementary material for: Exploring the components and mechanisms of Shen-qi-wang-mo granule in the treatment of retinal vein occlusion by UPLC-Triple TOF MS/MS and network pharmacology
Source: Sci Rep. 2023 Apr 1;13:5330. doi: 10.1038/s41598-023-32472-0 (PMC10066998; doi:10.1038/s41598-023-32472-0)
Supplement: Supplementary file 13 — Supplementary Table 7. [file 41598_2023_32472_MOESM13_ESM.docx]

| ***qPCR Primer*** | | | | | | | |
| --- | --- | --- | --- | --- | --- | --- | --- |
| **Gene** | **Forward primer (5′ to 3′ )** | | | | **Reverse primer (3′to 5′)** | | |
| VFGFR | CGCAAAGGAGACGCTAGACT | | | | TGTAAGCCAGGGTAAGGGGA | | |
| MAPK1 | TGAGGCTCCGTTCAAGTTCG | | | | AATCCAGACCAACGCCTGTG | | |
| MAP2K1 | GAGACCCATCAGCCAGTGAC | | | | GGTAGCTTTGGAGGTGGTTCA | | |
| MAPK14a | GTGCATACGGATCTGTTTGCTC | | | | TTGGCATGGATGATGGACTGAA | | |
| MAPK3 | AGATCAAAATCCTCCTGCGGTT | | | | TCTCCATCAGGTCCTGTACGAT | | |
| PIK3CA | CAACCAGAGGATTGGGCACT | | | | TGTCTGCTCAGGTGCTTCAG | | |
| AKT1 | CACACCACCTGGACAAGATGA | | | | CGCTGGCCGAGTATGAGAAT | | |
| NOS2a | CATTGGCAGGATCCAGTGGT | | | | CCTCCGTTGGTGGCATACTT | | |
| IL-6 | ACTCACCTCTTCAGAACGAATTG | | | | CCATCTTTGGAAGGTTCAGGTTG | | |
| TNFa | AAGTCGGGTGTATGGAGGGT | | | | GATTGCCCTGGGTCTTATGGA | | |
| MTOR | GCTACATTGGATGGTGCCCT | | | | CGTCCGAGGAGATGTTACCG | | |
| ***Eluting*** ***program / Column temperature: 30℃.*** | | | | | | | |
| **Time (min)** | | | **A%** | | | **B%** | |
| 0～3 | | | 0 | | | 100 | |
| 3～7 | | | 0～5 | | | 100～95 | |
| 7～12 | | | 5～10 | | | 95～90 | |
| 12～22 | | | 10～20 | | | 90～80 | |
| 22～30 | | | 20～30 | | | 80～70 | |
| 30～35 | | | 30～45 | | | 70～55 | |
| 35～40 | | | 45～70 | | | 55～30 | |
| 40～45 | | | 70～95 | | | 30～5 | |
| 45～47 | | | 95 | | | 5 | |
| 47～47.1 | | | 95～0 | | | 5～100 | |
| 47.1～50 | | | 0 | | | 100 | |
| ***Mass parameters*** | | | | | | | |
| **MS** | | **Parameters** | | **MS/MS** | | | **Parameters** |
| TOF mass range | | 50～1700 | | MS/MS mass range | | | 50～1250 |
| Ion Source Gas 1（psi） | | 50 | | Declustering Potential（V） | | | 100 |
| Ion Source Gas 2（psi） | | 50 | | Collision Energy（eV） | | | ±40 |
| Curtain Gas（psi） | | 35 | | Collision Energy Spread（eV） | | | 20 |
| Ion Spray Voltage Floating (V) | | -4500/5000 | | Ion Release Delay（ms） | | | 30 |
| Ion Source Temperature (°C) | | 500 | | Ion Release Width（ms） | | | 15 |
| Declustering Potential（V） | | 100 | |  | | |  |
| Collision Energy（eV） | | 10 | |  | | |  |
